# Supplementary material for: Which public health interventions are effective in reducing morbidity, mortality and health inequalities from infectious diseases amongst children in low- and middle-income countries (LMICs): An umbrella review
Source: PLoS One. 2021 Jun 10;16(6):e0251905. doi: 10.1371/journal.pone.0251905 (PMC8191901; doi:10.1371/journal.pone.0251905)
Supplement: S1 Appendix — (DOCX) [file pone.0251905.s001.docx]

# S1 Appendix: Types of public health interventions targeting infectious diseases or associated risk factors in children identified in previous research

The table below presents broad types of interventions for which systematic reviews reporting population health impacts have been identified in the Campbell Collaboration and UNICEF Office of Research- Innocenti evidence mega-map on child welfare(Campbell Collaboration & UNICEF Office of Research- Innocenti, 2018) and/or that have been identified by professionals and researchers contacted by the review team. These interventions have been categorized according to WHO EURO’s definitions of health promotion, protection and prevention.(WHO EURO, 2015)

|  | Health promotion  *Intersectoral and interdisciplinary operations enabling people to stay healthy or improve their health and its determinants.* | Health protection  *Use of legal, regulatory or enforcement mechanisms to safeguard public health* | Disease prevention  *Public health services within the health system that target individuals or populations at risk of developing a disease.* |
| --- | --- | --- | --- |
| Structural level | - Welfare system | - Environmental health standards and safety (e.g. water and air quality) | - Health systems resources allocated to prevention - Health system’s scope and coverage (as applied to preventive services) |
| Public policy level | - Universal Health coverage policies (as applied to children and their families) - Family policy - Financial assistance/Public assistance (e.g. conditional cash transfer) - Food policy - Access to health services policies | - Environmental health standard application and enforcement | - Water, sanitation and waste management infrastructure. - Child immunization policies and programs. |
| Social or community level | - Health and nutrition education - Food fortification and supplements | - Pollution and chemical exposure reduction interventions (e.g. cookstove distribution) | - Hygiene promotion - Parenting programs - Community outreach - Community health services - Vector control campaign (mosquito nets distribution, insecticide) - Deworming campaigns |
| Individual or household level | - Interventions promoting/changing nutrition practice (e.g. breastfeeding support, nutrition therapy, supplementation) | NA (see definition of health protection) | - Preventive health services (immunization, preventive treatment for HIV) - Psychosocial support - Counselling - Water, sanitation and hygiene (WAS) interventions |
