# Supplementary material for: Which public health interventions are effective in reducing morbidity, mortality and health inequalities from infectious diseases amongst children in low- and middle-income countries (LMICs): An umbrella review
Source: PLoS One. 2021 Jun 10;16(6):e0251905. doi: 10.1371/journal.pone.0251905 (PMC8191901; doi:10.1371/journal.pone.0251905)
Supplement: S4 Appendix — (DOCX) [file pone.0251905.s004.docx]

**S4 Appendix: Pilot search strings**

**Pilot search strings**

**Keywords**

*Pilot 1*

[**Population]**

*Title, Abstract, Key words=*

“developing countries” OR ( developing OR less developed OR under developed OR underdeveloped OR middle income OR low income OR lower income OR underserved OR under served OR deprived OR poor (*adj*) countr*)OR lmic OR lmics OR “third world” OR “lami countries” OR “global south” OR transition* OR

Africa OR Asia OR Caribbean OR “West Indies” OR “South America” OR “Latin America” OR “Middle East” OR “Central America”

AND

infant OR newborn OR neonate OR baby OR toddler OR pre-school OR pediatric OR child* OR perinatal OR kindergarten OR parent* (or narrowed search by age group)

[**Intervention**]

AND

*Title, Abstract, Keywords*=

health intervention OR public health OR health promotion OR prevention OR protection OR preventive health services OR preventive OR control

OR child health OR child welfare OR child care

OR policy OR program OR project

OR Communicable disease* (*Keyword*) OR infectious disease* (*Keyword*)

OR water OR sanitation OR latrine OR toilets OR “waste disposal” OR sewage OR hygiene OR hand wash* OR nutrition policy OR food policy OR breastfeeding OR nutrition therapy OR supplement OR parenting program OR counseling OR “health education” OR health knowledge/attitude/practice (Keyword) OR instruction OR immuni$ation OR vaccine OR immune* OR inoculation OR (prevent* (*adj*) transmission) OR insecticides OR mosquito control OR bed/mosquito nets OR Parasitic Diseases [prevention , control] (*Keywords*) OR vector control OR Prevention of mother-to-child transmission / PMTCT OR deworming OR community health OR outreach OR (psychosocial (*adj*) support) OR Psychosocial Support Systems (*keyword*) OR (social (*adj*) support) OR financing/organized (*keyword*) OR (finance* OR cash OR money (adj) transfer) OR saving* OR voucher OR microfinance OR microcredit OR Welfare OR public assistance OR social security OR insurance OR “family policy” OR environment* [Prevention, control]

AND

[**Type of study**]

*Title, Abstract, Keywords*=

(systematic review OR “evidence synthesis” OR Meta-Analysis (or narrowed by type)

NOT Editorial or Letter or Comment or Erratum or Conference paper or Survey or Note)

**Limit**: published since 2000

*Pilot 2*

**Pilot 1** AND

[**Outcome**]

*Title, Abstract, Keywords* =

effective* OR impact OR effect OR outcome assessment

OR (reported (*adj*) cases) OR (cases (*adj*) averted) OR morbidity OR mortality OR death* OR disease* OR illness OR coverage

OR (population health OR global health OR outcome assessment)

**Search string in Medline**

*Pilot 1*

**Number of hits:** 3,091 (as of 22/05/2019)

**Search string**:

| Type of study | 1 | exp Systematic Review/ or exp Meta Analysis/ or exp Meta-Analysis as Topic/ or exp Meta-Analysis/ or exp Review Literature as Topic/ |
| --- | --- | --- |
|  | 2 | (meta analy$ or metaanaly$ or (systematic adj (review$1 or overview$1))).tw. |
|  | 3 | (cochrane or embase or (psychlit or psyclit) or (cinahl or cinhal) or (psychinfo or psycinfo) or science citation index or bids or cancerlit).ab. |
|  | 4 | (reference list$ or bibliograph$ or hand-search$ or relevant journals or manual search$).ab. |
|  | 5 | (selection criteria or data extraction).ab. |
|  | 6 | evidence synthes$.tw. |
|  | 7 | ((systematic adj2 (review* or overview*)) or (umbrella adj2 review) or "review of reviews" or (systematic adj (review$1 or overview$1))).tw. |
|  | 8 | 1 or 2 or 3 or 4 or 5 or 6 or 7 |
|  | 9 | (Comment or Letter or Editorial or animal or Published Erratum).hw. or conference paper.tw. or (Surveys and Questionnaires).hw. or note.pt. |
|  | 10 | 8 not 9 |
| Population | 11 | (Africa or Asia or Caribbean or West Indies or South America or Middle East or Latin America or Central America).ab,hw,kf,kw,ti. |
|  | 12 | (developing countries or lmic or lmics or third world or lami countries or global south or transition*).ab,hw,kf,kw,ti. |
|  | 13 | ((developing or less developed or under developed or underdeveloped or middle income or low income or lower income or underserved or under served or deprived or poor) adj countr*).ab,hw,kf,kw,ti. |
|  | 14 | (low* adj (gdp or gnp or gross domestic or gross national)).ab,hw,kf,kw,ti. |
|  | 15 | 11 or 12 or 13 or 14 |
|  | 16 | exp Infant, Newborn/ or exp Child/ or exp Infant/ or exp Parents/ or exp Child, Preschool/ |
|  | 17 | (infant or newborn or neonate or baby or babies or toddler or pre-school or pediatric or child* or perinatal or kindergarten or parent*).ab,hw,kf,kw,ti. |
|  | 18 | 16 or 17 |
|  | 19 | 15 and 18 |
| Intervention | 20 | exp Child Care/ or child welfare/ or exp Child Health/ |
|  | 21 | exp Communicable Disease Control/ |
|  | 22 | exp Communicable Diseases/ or exp Public Health/ or exp Health Promotion/ or exp Public Health/ or exp PRIMARY PREVENTION/ or exp Preventive Health Services/ or exp "DELIVERY OF HEALTH CARE"/ |
|  | 23 | (health intervention or public health or health promotion or prevention or protection or preventive health services or preventive or control or child health or child welfare or child care or policy or program or project).ab,hw,kf,kw,ti. |
|  | 24 | (health adj8 (intervention$ or prevention or policy or policies or program$ or project$)).tw. |
|  | 25 | exp Public Policy/ or exp Organizational Policy/ or exp Fiscal Policy/ or exp Policy/ or exp Health Policy/ or exp Programs/ or exp Sewage/ or exp Bathroom Equipment/ or exp Water/ or exp Hygiene/ or exp Waste Management/ or exp Nutrition Therapy/ or exp Nutrition Policy/ or exp Health Education/ or exp Counseling/ or exp Health Knowledge, Attitudes, Practice/ or exp immunization/ or exp Immunization Programs/ or exp Community Health Aides/ or exp Social Support/ or exp Financing, Organized/ or exp maternal welfare/ or exp public policy/ or exp social welfare/ or exp social security/ or exp Environmental Health/ or exp Environmental Policy/ |
|  | 26 | (Communicable disease* or infectious disease* or water or sanitation or hygiene or health knowledge or Health attitude or practice or immunization or Parasitic Diseases or Psychosocial Support Systems or water or sanitation or latrine or toilets or waste disposal or sewage or hygiene or hand wash* or nutrition policy or food policy or breastfeeding or nutrition therapy or supplement or parenting program or counseling or health education or instruction or immuni#ation or vaccine or immune* or inoculation or insecticides or mosquito control or bed nets or mosquito nets or vector control or Prevention of mother-to-child transmission or PMTCT or deworming or community health or outreach or saving* or voucher or microfinance or microcredit or Welfare or public assistance or social security or insurance or family policy or environment*).tw. |
|  | 27 | ((psychosocial adj3 support) or (social adj3 support) or (prevent* adj3 transmission) or ((finance* or cash or money) adj3 transfer) or (environment adj3 (prevent* or control*))).tw. |
|  | 28 | 20 or 21 or 22 or 23 or 24 |
|  | 29 | 25 or 26 or 27 |
|  | 30 | 28 or 29 |
| P+I+S | 31 | 10 and 19 and 30 |
| Year | 32 | limit 31 to yr="2000 -Current" |

*Pilot 2*

**Number of hits** (as of 22/05/2019): 2,404

**Search string**:

| Type of study | 1 | exp Systematic Review/ or exp Meta Analysis/ or exp Meta-Analysis as Topic/ or exp Meta-Analysis/ or exp Review Literature as Topic/ |
| --- | --- | --- |
|  | 2 | (meta analy$ or metaanaly$ or (systematic adj (review$1 or overview$1))).tw. |
|  | 3 | (cochrane or embase or (psychlit or psyclit) or (cinahl or cinhal) or (psychinfo or psycinfo) or science citation index or bids or cancerlit).ab. |
|  | 4 | (reference list$ or bibliograph$ or hand-search$ or relevant journals or manual search$).ab. |
|  | 5 | (selection criteria or data extraction).ab. |
|  | 6 | evidence synthes$.tw. |
|  | 7 | ((systematic adj2 (review* or overview*)) or (umbrella adj2 review) or "review of reviews" or (systematic adj (review$1 or overview$1))).tw. |
|  | 8 | 1 or 2 or 3 or 4 or 5 or 6 or 7 |
|  | 9 | (Comment or Letter or Editorial or animal or Published Erratum).hw. or conference paper.tw. or (Surveys and Questionnaires).hw. or note.pt. |
|  | 10 | 8 not 9 |
| Population | 11 | (Africa or Asia or Caribbean or West Indies or South America or Middle East or Latin America or Central America).ab,hw,kf,kw,ti. |
|  | 12 | (developing countries or lmic or lmics or third world or lami countries or global south or transition*).ab,hw,kf,kw,ti. |
|  | 13 | ((developing or less developed or under developed or underdeveloped or middle income or low income or lower income or underserved or under served or deprived or poor) adj countr*).ab,hw,kf,kw,ti. |
|  | 14 | (low* adj (gdp or gnp or gross domestic or gross national)).ab,hw,kf,kw,ti. |
|  | 15 | 11 or 12 or 13 or 14 |
|  | 16 | exp Infant, Newborn/ or exp Child/ or exp Infant/ or exp Parents/ or exp Child, Preschool/ |
|  | 17 | (infant or newborn or neonate or baby or babies or toddler or pre-school or pediatric or child* or perinatal or kindergarten or parent*).ab,hw,kf,kw,ti. |
|  | 18 | 16 or 17 |
|  | 19 | 15 and 18 |
| Intervention | 20 | exp Child Care/ or child welfare/ or exp Child Health/ |
|  | 21 | exp Communicable Disease Control/ |
|  | 22 | exp Communicable Diseases/ or exp Public Health/ or exp Health Promotion/ or exp Public Health/ or exp PRIMARY PREVENTION/ or exp Preventive Health Services/ or exp "DELIVERY OF HEALTH CARE"/ |
|  | 23 | (health intervention or public health or health promotion or prevention or protection or preventive health services or preventive or control or child health or child welfare or child care or policy or program or project).ab,hw,kf,kw,ti. |
|  | 24 | (health adj8 (intervention$ or prevention or policy or policies or program$ or project$)).tw. |
|  | 25 | exp Public Policy/ or exp Organizational Policy/ or exp Fiscal Policy/ or exp Policy/ or exp Health Policy/ or exp Programs/ or exp Sewage/ or exp Bathroom Equipment/ or exp Water/ or exp Hygiene/ or exp Waste Management/ or exp Nutrition Therapy/ or exp Nutrition Policy/ or exp Health Education/ or exp Counseling/ or exp Health Knowledge, Attitudes, Practice/ or exp immunization/ or exp Immunization Programs/ or exp Community Health Aides/ or exp Social Support/ or exp Financing, Organized/ or exp maternal welfare/ or exp public policy/ or exp social welfare/ or exp social security/ or exp Environmental Health/ or exp Environmental Policy/ |
|  | 26 | (Communicable disease* or infectious disease* or water or sanitation or hygiene or health knowledge or Health attitude or practice or immunization or Parasitic Diseases or Psychosocial Support Systems or water or sanitation or latrine or toilets or waste disposal or sewage or hygiene or hand wash* or nutrition policy or food policy or breastfeeding or nutrition therapy or supplement or parenting program or counseling or health education or instruction or immuni#ation or vaccine or immune* or inoculation or insecticides or mosquito control or bed nets or mosquito nets or vector control or Prevention of mother-to-child transmission or PMTCT or deworming or community health or outreach or saving* or voucher or microfinance or microcredit or Welfare or public assistance or social security or insurance or family policy or environment*).tw. |
|  | 27 | ((psychosocial adj3 support) or (social adj3 support) or (prevent* adj3 transmission) or ((finance* or cash or money) adj3 transfer) or (environment adj3 (prevent* or control*))).tw. |
|  | 28 | 20 or 21 or 22 or 23 or 24 |
|  | 29 | 25 or 26 or 27 |
|  | 30 | 28 or 29 |
| P+I+S | 31 | 10 and 19 and 30 |
| Outcomes | 32 | exp Population Health/ or exp Global Health/ or exp "Outcome Assessment (Health Care)"/ |
|  | 33 | (population health or global health or outcome assessment).ab,hw,kf,kw,ti. |
|  | 34 | 32 or 33 |
|  | 35 | (effective* or impact or effect or outcome assessment or morbidity or mortality or death* or disease* or illness or coverage or (reported adj3 cases) or (cases adj3 averted)).tw. |
|  | 36 | 34 or 35 |
| P+I+O+S | 37 | 31 and 36 |
| Year | 38 | limit 37 to yr="2000 -Current" |

**Search string in Scopus**

*Pilot 1*

**Number of hits** (as of 22/05/2019): 4,148

**Search string**:

( ( ( ( TITLE-ABS-KEY ( low* W/2 gdp ) OR TITLE-ABS-KEY ( low* W/2 gnp ) OR TITLE-ABS-KEY ( low* W/2 "gross domestic" ) OR TITLE-ABS-KEY ( low* W/2 "gross national" ) ) ) OR ( ( TITLE-ABS-KEY ( africa OR asia OR caribbean OR "West Indies" OR "South America" OR "Middle East" OR "Latin America" OR "Central America" ) ) OR ( TITLE-ABS-KEY ( "developing countries" OR lmic OR lmics OR "third world" OR "lami countries" OR "global south" OR transition* ) ) OR ( ( TITLE-ABS-KEY ( developing W/2 countr* ) OR TITLE-ABS-KEY ( "less developed" W/2 countr* ) OR TITLE-ABS-KEY ( "under developed" W/2 countr* ) OR TITLE-ABS-KEY ( underdeveloped W/2 countr* ) OR TITLE-ABS-KEY ( "middle income" W/2 countr* ) OR TITLE-ABS-KEY ( "low income" W/2 countr* ) OR TITLE-ABS-KEY ( "lower income" W/2 countr* ) OR TITLE-ABS-KEY ( underserved W/2 countr* ) OR TITLE-ABS-KEY ( "under served" W/2 countr* ) OR TITLE-ABS-KEY ( deprived W/2 countr* ) OR TITLE-ABS-KEY ( poor W/2 countr* ) ) ) ) )

AND ( TITLE-ABS-KEY ( infant OR newborn OR neonate OR baby OR babies OR toddler OR ( pre PRE/0 school ) OR pediatric OR child* OR perinatal OR kindergarten OR parent* ) ) )

AND ( ( ( TITLE-ABS-KEY ( health W/3 intervention* ) OR TITLE-ABS-KEY ( health W/3 prevention ) OR TITLE-ABS-KEY ( health W/3 policy ) OR TITLE-ABS-KEY ( health W/3 policies ) OR TITLE-ABS-KEY ( health W/3 program* ) OR TITLE-ABS-KEY ( health W/3 project* ) ) ) OR ( KEY ( "Public Policy" ) OR KEY ( "Organizational Policy" ) OR KEY ( "Fiscal Policy" ) OR KEY ( "Policy" ) OR KEY ( "Health Policy" ) OR KEY ( "Programs" ) OR KEY ( "Sewage" ) OR KEY ( "Bathroom Equipment" ) OR KEY ( "Water" ) OR KEY ( "Hygiene" ) OR KEY ( "Waste Management" ) OR KEY ( "Nutrition Therapy" ) OR KEY ( "Nutrition Policy" ) OR KEY ( "Health Education" ) OR KEY ( "Counseling" ) OR KEY ( "Health Knowledge, Attitudes, Practice" ) OR KEY ( "immunization" ) OR KEY ( "Immunization Programs" ) OR KEY ( "Community Health Aides" ) OR KEY ( "Social Support" ) OR KEY ( "Financing, Organized" ) OR KEY ( "maternal welfare" ) OR KEY ( "public policy" ) OR KEY ( "social welfare" ) OR KEY ( "social security" ) OR KEY ( "Environmental Health" ) OR KEY ( "Environmental Policy" ) ) OR ( ( ( TITLE-ABS-KEY ( prevent* W/3 transmission ) OR TITLE-ABS-KEY ( psychosocial W/3 support ) OR TITLE-ABS-KEY ( social W/3 support ) OR TITLE-ABS-KEY ( finance* W/3 transfer ) OR TITLE-ABS-KEY ( cash W/3 transfer ) OR TITLE-ABS-KEY ( money W/3 transfer ) OR TITLE-ABS-KEY ( environment* W/3 prevent* ) OR TITLE-ABS-KEY ( environment* W/3 control* ) ) ) OR ( TITLE-ABS-KEY ( "communicable diseases" OR "infectious diseases" OR "health knowledge" OR "health attitude" OR "health practice" OR water OR sanitation OR waste OR hygiene OR latrine OR toilets OR "waste disposal" OR sewage OR "hand washing" OR "nutrition policy" OR "food policy" OR breastfeeding OR "nutrition therapy" OR supplement OR "parenting program" OR counseling OR "health education" OR instruction OR immunisation OR immunization OR vaccine OR immun* OR inoculation OR insecticides OR "mosquito control" OR "bed nets" OR "mosquito nets" OR "vector control" OR "Prevention of mother-to-child transmission" OR pmtct OR deworming OR "community health" OR outreach OR saving OR voucher OR microfinance OR microcredit OR welfare OR "public assistance" OR "social security" OR insurance OR "family policy" OR "family policies" ) ) OR ( ( TITLE-ABS-KEY ( "health intervention" OR "public health" OR "health promotion" OR prevention OR protection OR "preventive health services" OR preventive OR control OR "child health" OR "child welfare" OR "child care" OR policy OR program OR project ) OR TITLE-ABS-KEY ( "Parasitic Diseases" OR finance* OR welfare OR environment* ) OR TITLE-ABS-KEY ( "Psychosocial Support System" ) ) ) ) )

AND ( ( ( ( KEY ( "Systematic Review" ) OR KEY ( "Meta Analysis" ) OR KEY ( "Meta-Analysis as Topic" ) OR KEY ( "Meta-Analysis" ) OR KEY ( "Review Literature as Topic" ) ) ) OR ( ( TITLE-ABS-KEY ( "meta analysis" OR "metaanalysis" OR "Meta-Analysis" OR "systematic review" OR overview* OR "evidence synthesis" OR "review of reviews" ) OR TITLE-ABS-KEY ( systematic W/2 review* ) OR TITLE-ABS-KEY ( systematic W/2 overview* ) OR TITLE-ABS-KEY ( umbrella W/2 review ) ) ) OR ( ( ABS ( cochrane OR embase OR psychlit OR psyclit ) OR ABS ( cinahl OR cinhal ) OR ABS ( psychinfo OR psycinfo ) OR ABS ( "science citation index" OR bids OR cancerlit ) ) ) OR ( ( ABS ( "reference list" OR "reference lists" OR bibliography* OR hand-search* OR "relevant journals" OR "manual search" OR "manual searches" ) OR ABS ( "selection criteria" OR "data extraction" ) ) ) ) AND NOT ( TITLE-ABS-KEY ( comment OR letter OR editorial OR animal ) ) )

AND ( PUBYEAR > 2000 )

AND ( EXCLUDE ( DOCTYPE , "sh" ) OR EXCLUDE ( DOCTYPE , "no" ) OR EXCLUDE ( DOCTYPE , "er" ) OR EXCLUDE ( DOCTYPE , "ed" ) OR EXCLUDE ( DOCTYPE , "cp" ) OR EXCLUDE ( DOCTYPE , "cr" ) )

*Pilot 2*

**Number of hits** (as of 22/05/2019): 3,572

**Search string**:

( ( ( ( TITLE-ABS-KEY ( low* W/2 gdp ) OR TITLE-ABS-KEY ( low* W/2 gnp ) OR TITLE-ABS-KEY ( low* W/2 "gross domestic" ) OR TITLE-ABS-KEY ( low* W/2 "gross national" ) ) ) OR ( ( TITLE-ABS-KEY ( africa OR asia OR caribbean OR "West Indies" OR "South America" OR "Middle East" OR "Latin America" OR "Central America" ) ) OR ( TITLE-ABS-KEY ( "developing countries" OR lmic OR lmics OR "third world" OR "lami countries" OR "global south" OR transition* ) ) OR ( ( TITLE-ABS-KEY ( developing W/2 countr* ) OR TITLE-ABS-KEY ( "less developed" W/2 countr* ) OR TITLE-ABS-KEY ( "under developed" W/2 countr* ) OR TITLE-ABS-KEY ( underdeveloped W/2 countr* ) OR TITLE-ABS-KEY ( "middle income" W/2 countr* ) OR TITLE-ABS-KEY ( "low income" W/2 countr* ) OR TITLE-ABS-KEY ( "lower income" W/2 countr* ) OR TITLE-ABS-KEY ( underserved W/2 countr* ) OR TITLE-ABS-KEY ( "under served" W/2 countr* ) OR TITLE-ABS-KEY ( deprived W/2 countr* ) OR TITLE-ABS-KEY ( poor W/2 countr* ) ) ) ) )

AND ( TITLE-ABS-KEY ( infant OR newborn OR neonate OR baby OR babies OR toddler OR ( pre PRE/0 school ) OR pediatric OR child* OR perinatal OR kindergarten OR parent* ) ) )

AND ( ( ( TITLE-ABS-KEY ( health W/3 intervention* ) OR TITLE-ABS-KEY ( health W/3 prevention ) OR TITLE-ABS-KEY ( health W/3 policy ) OR TITLE-ABS-KEY ( health W/3 policies ) OR TITLE-ABS-KEY ( health W/3 program* ) OR TITLE-ABS-KEY ( health W/3 project* ) ) ) OR ( KEY ( "Public Policy" ) OR KEY ( "Organizational Policy" ) OR KEY ( "Fiscal Policy" ) OR KEY ( "Policy" ) OR KEY ( "Health Policy" ) OR KEY ( "Programs" ) OR KEY ( "Sewage" ) OR KEY ( "Bathroom Equipment" ) OR KEY ( "Water" ) OR KEY ( "Hygiene" ) OR KEY ( "Waste Management" ) OR KEY ( "Nutrition Therapy" ) OR KEY ( "Nutrition Policy" ) OR KEY ( "Health Education" ) OR KEY ( "Counseling" ) OR KEY ( "Health Knowledge, Attitudes, Practice" ) OR KEY ( "immunization" ) OR KEY ( "Immunization Programs" ) OR KEY ( "Community Health Aides" ) OR KEY ( "Social Support" ) OR KEY ( "Financing, Organized" ) OR KEY ( "maternal welfare" ) OR KEY ( "public policy" ) OR KEY ( "social welfare" ) OR KEY ( "social security" ) OR KEY ( "Environmental Health" ) OR KEY ( "Environmental Policy" ) ) OR ( ( ( TITLE-ABS-KEY ( prevent* W/3 transmission ) OR TITLE-ABS-KEY ( psychosocial W/3 support ) OR TITLE-ABS-KEY ( social W/3 support ) OR TITLE-ABS-KEY ( finance* W/3 transfer ) OR TITLE-ABS-KEY ( cash W/3 transfer ) OR TITLE-ABS-KEY ( money W/3 transfer ) OR TITLE-ABS-KEY ( environment* W/3 prevent* ) OR TITLE-ABS-KEY ( environment* W/3 control* ) ) ) OR ( TITLE-ABS-KEY ( "communicable diseases" OR "infectious diseases" OR "health knowledge" OR "health attitude" OR "health practice" OR water OR sanitation OR waste OR hygiene OR latrine OR toilets OR "waste disposal" OR sewage OR "hand washing" OR "nutrition policy" OR "food policy" OR breastfeeding OR "nutrition therapy" OR supplement OR "parenting program" OR counseling OR "health education" OR instruction OR immunisation OR immunization OR vaccine OR immun* OR inoculation OR insecticides OR "mosquito control" OR "bed nets" OR "mosquito nets" OR "vector control" OR "Prevention of mother-to-child transmission" OR pmtct OR deworming OR "community health" OR outreach OR saving OR voucher OR microfinance OR microcredit OR welfare OR "public assistance" OR "social security" OR insurance OR "family policy" OR "family policies" ) ) OR ( ( TITLE-ABS-KEY ( "health intervention" OR "public health" OR "health promotion" OR prevention OR protection OR "preventive health services" OR preventive OR control OR "child health" OR "child welfare" OR "child care" OR policy OR program OR project ) OR TITLE-ABS-KEY ( "Parasitic Diseases" OR finance* OR welfare OR environment* ) OR TITLE-ABS-KEY ( "Psychosocial Support System" ) ) ) ) )

AND ( ( ( ( KEY ( "Systematic Review" ) OR KEY ( "Meta Analysis" ) OR KEY ( "Meta-Analysis as Topic" ) OR KEY ( "Meta-Analysis" ) OR KEY ( "Review Literature as Topic" ) ) ) OR ( ( TITLE-ABS-KEY ( "meta analysis" OR "metaanalysis" OR "Meta-Analysis" OR "systematic review" OR overview* OR "evidence synthesis" OR "review of reviews" ) OR TITLE-ABS-KEY ( systematic W/2 review* ) OR TITLE-ABS-KEY ( systematic W/2 overview* ) OR TITLE-ABS-KEY ( umbrella W/2 review ) ) ) OR ( ( ABS ( cochrane OR embase OR psychlit OR psyclit ) OR ABS ( cinahl OR cinhal ) OR ABS ( psychinfo OR psycinfo ) OR ABS ( "science citation index" OR bids OR cancerlit ) ) ) OR ( ( ABS ( "reference list" OR "reference lists" OR bibliography* OR hand-search* OR "relevant journals" OR "manual search" OR "manual searches" ) OR ABS ( "selection criteria" OR "data extraction" ) ) ) ) AND NOT ( TITLE-ABS-KEY ( comment OR letter OR editorial OR animal ) ) )

AND ( ( TITLE-ABS-KEY ( "population health" OR "global health" OR "outcome assessment" ) OR TITLE-ABS-KEY ( effectiv* OR impact OR effect OR "outcome assessment" OR morbidity OR mortality OR death* OR disease* OR illness OR coverage ) OR TITLE-ABS-KEY ( reported W/3 cases ) OR TITLE-ABS-KEY ( cases W/3 averted ) ) )

AND ( PUBYEAR > 2000 )

AND ( EXCLUDE ( DOCTYPE , "sh" ) OR EXCLUDE ( DOCTYPE , "no" ) OR EXCLUDE ( DOCTYPE , "er" ) OR EXCLUDE ( DOCTYPE , "ed" ) OR EXCLUDE ( DOCTYPE , "cp" ) OR EXCLUDE ( DOCTYPE , "cr" ) )

**Pilot search results**

A selection of four tracer systematic reviews and evidence syntheses (namely (Crocker-Buque et al., 2017; Gilmore & McAuliffe, 2013; Owusu-Addo & Cross, 2014; Yuan et al., 2014)) was used to test whether the different search strings identified them. The choice of the final search string was made based on the result of each stage

|  | Pilot 1 | | Pilot 2 | |
| --- | --- | --- | --- | --- |
| Database | Medline | Scopus | Medline | Scopus |
| Number of hits (22/05/2019) | 3,091 | 4,148 | 2,404 | 3,572 |
| (Yuan et al., 2014) |  |  |  |  |
| (Crocker-Buque et al., 2017) |  |  |  |  |
| (Owusu-Addo & Cross, 2014) |  |  |  |  |
| (Gilmore & McAuliffe, 2013) |  |  |  |  |
