# Supplementary material for: Which public health interventions are effective in reducing morbidity, mortality and health inequalities from infectious diseases amongst children in low- and middle-income countries (LMICs): An umbrella review
Source: PLoS One. 2021 Jun 10;16(6):e0251905. doi: 10.1371/journal.pone.0251905 (PMC8191901; doi:10.1371/journal.pone.0251905)
Supplement: S5 Appendix — (DOCX) [file pone.0251905.s005.docx]

**S5 APPENDIX: Full search strategy**

**Keywords**

[**Population]**

*Title, Abstract, Key words=*

“developing countries” OR ( developing OR less developed OR under developed OR underdeveloped OR middle income OR low income OR lower income OR underserved OR under served OR deprived OR poor (*adj*) countr*)OR lmic OR lmics OR “third world” OR “lami countries” OR “global south” OR transition* OR

Africa OR Asia OR Caribbean OR “West Indies” OR “South America” OR “Middle East” OR “Latin America” OR “Central America”

AND

infant OR newborn OR neonate OR baby OR toddler OR pre-school OR pediatric OR child* OR perinatal OR kindergarten OR parent* (or narrowed search by age group)

[**Intervention**]

AND

*Title, Abstract, Keywords*= health intervention OR public health OR health promotion OR prevention OR protection OR preventive health services OR preventive OR control

OR child health OR child welfare OR child care

OR policy OR program OR project

OR Communicable disease* (*Keyword*) OR infectious disease* (*Keyword*)

OR water OR sanitation OR latrine OR toilets OR “waste disposal” OR sewage OR hygiene OR hand wash* OR nutrition policy OR food policy OR breastfeeding OR nutrition therapy OR supplement OR parenting program OR counseling OR “health education” OR health knowledge/attitude/practice (Keyword) OR instruction OR immuni$ation OR vaccine OR immune* OR inoculation OR (prevent* (*adj*) transmission) OR insecticides OR mosquito control OR bed/mosquito nets OR Parasitic Diseases [prevention , control] (*Keywords*) OR vector control OR Prevention of mother-to-child transmission / PMTCT OR deworming OR community health OR outreach OR (psychosocial (*adj*) support) OR Psychosocial Support Systems (*keyword*) OR (social (*adj*) support) OR financing/organized (*keyword*) OR (finance* OR cash OR money (adj) transfer) OR saving* OR voucher OR microfinance OR microcredit OR Welfare OR public assistance OR social security OR insurance OR “family policy” OR environment* [Prevention, control]

AND

[**Type of study**] *Title, Abstract, Keywords*= (

systematic review OR “evidence synthesis” OR Meta-Analysis (or narrowed by type)

NOT Editorial or Letter or Comment or Erratum or Conference paper or Survey or Note)

AND

[**Outcome**]

*Title, Abstract, Keywords* =

effective* OR impact OR effect OR outcome assessment

OR (reported (*adj*) cases) OR (cases (*adj*) averted) OR morbidity OR mortality OR death* OR disease* OR illness OR coverage

OR (population health OR global health OR outcome assessment)

**Limit**: published since 2000

**Databases searches**

Search string in Medline

Ovid MEDLINE(R) and Epub Ahead of Print, In-Process & Other Non-Indexed Citations and Daily 1946 to present

Initial search

**Number of hits** (as of 19/06/2019): 2,444

**Search string**:

| Type of study | 1 | exp Systematic Review/ or exp Meta Analysis/ or exp Meta-Analysis as Topic/ or exp Meta-Analysis/ or exp Review Literature as Topic/ |
| --- | --- | --- |
|  | 2 | (meta analy$ or metaanaly$ or (systematic adj (review$1 or overview$1))).tw. |
|  | 3 | (cochrane or embase or (psychlit or psyclit) or (cinahl or cinhal) or (psychinfo or psycinfo) or science citation index or bids or cancerlit).ab. |
|  | 4 | (reference list$ or bibliograph$ or hand-search$ or relevant journals or manual search$).ab. |
|  | 5 | (selection criteria or data extraction).ab. |
|  | 6 | evidence synthes$.tw. |
|  | 7 | ((systematic adj2 (review* or overview*)) or (umbrella adj2 review) or "review of reviews" or (systematic adj (review$1 or overview$1))).tw. |
|  | 8 | 1 or 2 or 3 or 4 or 5 or 6 or 7 |
|  | 9 | (Comment or Letter or Editorial or animal or Published Erratum).hw. or conference paper.tw. or (Surveys and Questionnaires).hw. or note.pt. |
|  | 10 | 8 not 9 |
| Population | 11 | (Africa or Asia or Caribbean or West Indies or South America or Middle East or Latin America or Central America).ab,hw,kf,kw,ti. |
|  | 12 | (developing countries or lmic or lmics or third world or lami countries or global south or transition*).ab,hw,kf,kw,ti. |
|  | 13 | ((developing or less developed or under developed or underdeveloped or middle income or low income or lower income or underserved or under served or deprived or poor) adj countr*).ab,hw,kf,kw,ti. |
|  | 14 | (low* adj (gdp or gnp or gross domestic or gross national)).ab,hw,kf,kw,ti. |
|  | 15 | 11 or 12 or 13 or 14 |
|  | 16 | exp Infant, Newborn/ or exp Child/ or exp Infant/ or exp Parents/ or exp Child, Preschool/ |
|  | 17 | (infant or newborn or neonate or baby or babies or toddler or pre-school or pediatric or child* or perinatal or kindergarten or parent*).ab,hw,kf,kw,ti. |
|  | 18 | 16 or 17 |
|  | 19 | 15 and 18 |
| Intervention | 20 | exp Child Care/ or child welfare/ or exp Child Health/ |
|  | 21 | exp Communicable Disease Control/ |
|  | 22 | exp Communicable Diseases/ or exp Public Health/ or exp Health Promotion/ or exp Public Health/ or exp PRIMARY PREVENTION/ or exp Preventive Health Services/ or exp "DELIVERY OF HEALTH CARE"/ |
|  | 23 | (health intervention or public health or health promotion or prevention or protection or preventive health services or preventive or control or child health or child welfare or child care or policy or program or project).ab,hw,kf,kw,ti. |
|  | 24 | (health adj8 (intervention$ or prevention or policy or policies or program$ or project$)).tw. |
|  | 25 | exp Public Policy/ or exp Organizational Policy/ or exp Fiscal Policy/ or exp Policy/ or exp Health Policy/ or exp Programs/ or exp Sewage/ or exp Bathroom Equipment/ or exp Water/ or exp Hygiene/ or exp Waste Management/ or exp Nutrition Therapy/ or exp Nutrition Policy/ or exp Health Education/ or exp Counseling/ or exp Health Knowledge, Attitudes, Practice/ or exp immunization/ or exp Immunization Programs/ or exp Community Health Aides/ or exp Social Support/ or exp Financing, Organized/ or exp maternal welfare/ or exp public policy/ or exp social welfare/ or exp social security/ or exp Environmental Health/ or exp Environmental Policy/ |
|  | 26 | (Communicable disease* or infectious disease* or water or sanitation or hygiene or health knowledge or Health attitude or practice or immunization or Parasitic Diseases or Psychosocial Support Systems or water or sanitation or latrine or toilets or waste disposal or sewage or hygiene or hand wash* or nutrition policy or food policy or breastfeeding or nutrition therapy or supplement or parenting program or counseling or health education or instruction or immuni#ation or vaccine or immune* or inoculation or insecticides or mosquito control or bed nets or mosquito nets or vector control or Prevention of mother-to-child transmission or PMTCT or deworming or community health or outreach or saving* or voucher or microfinance or microcredit or Welfare or public assistance or social security or insurance or family policy or environment*).tw. |
|  | 27 | ((psychosocial adj3 support) or (social adj3 support) or (prevent* adj3 transmission) or ((finance* or cash or money) adj3 transfer) or (environment adj3 (prevent* or control*))).tw. |
|  | 28 | 20 or 21 or 22 or 23 or 24 |
|  | 29 | 25 or 26 or 27 |
|  | 30 | 28 or 29 |
| P+I+S | 31 | 10 and 19 and 30 |
| Outcomes | 32 | exp Population Health/ or exp Global Health/ or exp "Outcome Assessment (Health Care)"/ |
|  | 33 | (population health or global health or outcome assessment).ab,hw,kf,kw,ti. |
|  | 34 | 32 or 33 |
|  | 35 | (effective* or impact or effect or outcome assessment or morbidity or mortality or death* or disease* or illness or coverage or (reported adj3 cases) or (cases adj3 averted)).tw. |
|  | 36 | 34 or 35 |
| P+I+O+S | 37 | 31 and 36 |
| Year | 38 | limit 37 to yr="2000 -Current" |

Updated search

**Number of hits** (as of 05/01/2021): 3,161

**Search string**:

| Type of study | 1 | exp Systematic Review/ or exp Meta Analysis/ or exp Meta-Analysis as Topic/ or exp Meta-Analysis/ or exp Review Literature as Topic/ |
| --- | --- | --- |
|  | 2 | (meta analy$ or metaanaly$ or (systematic adj (review$1 or overview$1))).tw. |
|  | 3 | (cochrane or embase or (psychlit or psyclit) or (cinahl or cinhal) or (psychinfo or psycinfo) or science citation index or bids or cancerlit).ab. |
|  | 4 | (reference list$ or bibliograph$ or hand-search$ or relevant journals or manual search$).ab. |
|  | 5 | (selection criteria or data extraction).ab. |
|  | 6 | evidence synthes$.tw. |
|  | 7 | ((systematic adj2 (review* or overview*)) or (umbrella adj2 review) or "review of reviews" or (systematic adj (review$1 or overview$1))).tw. |
|  | 8 | 1 or 2 or 3 or 4 or 5 or 6 or 7 |
|  | 9 | (Comment or Letter or Editorial or animal or Published Erratum).hw. or conference paper.tw. or (Surveys and Questionnaires).hw. or note.pt. |
|  | 10 | 8 not 9 |
| Population | 11 | (Africa or Asia or Caribbean or West Indies or South America or Middle East or Latin America or Central America).ab,hw,kf,kw,ti. |
|  | 12 | (developing countries or lmic or lmics or third world or lami countries or global south or transition*).ab,hw,kf,kw,ti. |
|  | 13 | ((developing or less developed or under developed or underdeveloped or middle income or low income or lower income or underserved or under served or deprived or poor) adj countr*).ab,hw,kf,kw,ti. |
|  | 14 | (low* adj (gdp or gnp or gross domestic or gross national)).ab,hw,kf,kw,ti. |
|  | 15 | 11 or 12 or 13 or 14 |
|  | 16 | exp Infant, Newborn/ or exp Child/ or exp Infant/ or exp Parents/ or exp Child, Preschool/ |
|  | 17 | (infant or newborn or neonate or baby or babies or toddler or pre-school or pediatric or child* or perinatal or kindergarten or parent*).ab,hw,kf,kw,ti. |
|  | 18 | 16 or 17 |
|  | 19 | 15 and 18 |
| Intervention | 20 | exp Child Care/ or child welfare/ or exp Child Health/ |
|  | 21 | exp Communicable Disease Control/ |
|  | 22 | exp Communicable Diseases/ or exp Public Health/ or exp Health Promotion/ or exp Public Health/ or exp PRIMARY PREVENTION/ or exp Preventive Health Services/ or exp "DELIVERY OF HEALTH CARE"/ |
|  | 23 | (health intervention or public health or health promotion or prevention or protection or preventive health services or preventive or control or child health or child welfare or child care or policy or program or project).ab,hw,kf,kw,ti. |
|  | 24 | (health adj8 (intervention$ or prevention or policy or policies or program$ or project$)).tw. |
|  | 25 | exp Public Policy/ or exp Organizational Policy/ or exp Fiscal Policy/ or exp Policy/ or exp Health Policy/ or exp Programs/ or exp Sewage/ or exp Bathroom Equipment/ or exp Water/ or exp Hygiene/ or exp Waste Management/ or exp Nutrition Therapy/ or exp Nutrition Policy/ or exp Health Education/ or exp Counseling/ or exp Health Knowledge, Attitudes, Practice/ or exp immunization/ or exp Immunization Programs/ or exp Community Health Aides/ or exp Social Support/ or exp Financing, Organized/ or exp maternal welfare/ or exp public policy/ or exp social welfare/ or exp social security/ or exp Environmental Health/ or exp Environmental Policy/ |
|  | 26 | (Communicable disease* or infectious disease* or water or sanitation or hygiene or health knowledge or Health attitude or practice or immunization or Parasitic Diseases or Psychosocial Support Systems or water or sanitation or latrine or toilets or waste disposal or sewage or hygiene or hand wash* or nutrition policy or food policy or breastfeeding or nutrition therapy or supplement or parenting program or counseling or health education or instruction or immuni#ation or vaccine or immune* or inoculation or insecticides or mosquito control or bed nets or mosquito nets or vector control or Prevention of mother-to-child transmission or PMTCT or deworming or community health or outreach or saving* or voucher or microfinance or microcredit or Welfare or public assistance or social security or insurance or family policy or environment*).tw. |
|  | 27 | ((psychosocial adj3 support) or (social adj3 support) or (prevent* adj3 transmission) or ((finance* or cash or money) adj3 transfer) or (environment adj3 (prevent* or control*))).tw. |
|  | 28 | 20 or 21 or 22 or 23 or 24 |
|  | 29 | 25 or 26 or 27 |
|  | 30 | 28 or 29 |
| P+I+S | 31 | 10 and 19 and 30 |
| Outcomes | 32 | exp Population Health/ or exp Global Health/ or exp "Outcome Assessment (Health Care)"/ |
|  | 33 | (population health or global health or outcome assessment).ab,hw,kf,kw,ti. |
|  | 34 | 32 or 33 |
|  | 35 | (effective* or impact or effect or outcome assessment or morbidity or mortality or death* or disease* or illness or coverage or (reported adj3 cases) or (cases adj3 averted)).tw. |
|  | 36 | 34 or 35 |
| P+I+O+S | 37 | 31 and 36 |
| Year | 38 | limit 37 to yr="2000 -Current" |
|  | 39 | limit 38 to yr="2019 -Current" |

Search string in Scopus

<https://www.scopus.com/>

Initial search

**Number of hits** (as of 19/06/2019): 3,606

**Search string**:

( ( ( ( TITLE-ABS-KEY ( low* W/2 gdp ) OR TITLE-ABS-KEY ( low* W/2 gnp ) OR TITLE-ABS-KEY ( low* W/2 "gross domestic" ) OR TITLE-ABS-KEY ( low* W/2 "gross national" ) ) ) OR ( ( TITLE-ABS-KEY ( africa OR asia OR caribbean OR "West Indies" OR "South America" OR "Middle East" OR "Latin America" OR "Central America" ) ) OR ( TITLE-ABS-KEY ( "developing countries" OR lmic OR lmics OR "third world" OR "lami countries" OR "global south" OR transition* ) ) OR ( ( TITLE-ABS-KEY ( developing W/2 countr* ) OR TITLE-ABS-KEY ( "less developed" W/2 countr* ) OR TITLE-ABS-KEY ( "under developed" W/2 countr* ) OR TITLE-ABS-KEY ( underdeveloped W/2 countr* ) OR TITLE-ABS-KEY ( "middle income" W/2 countr* ) OR TITLE-ABS-KEY ( "low income" W/2 countr* ) OR TITLE-ABS-KEY ( "lower income" W/2 countr* ) OR TITLE-ABS-KEY ( underserved W/2 countr* ) OR TITLE-ABS-KEY ( "under served" W/2 countr* ) OR TITLE-ABS-KEY ( deprived W/2 countr* ) OR TITLE-ABS-KEY ( poor W/2 countr* ) ) ) ) )

AND ( TITLE-ABS-KEY ( infant OR newborn OR neonate OR baby OR babies OR toddler OR ( pre PRE/0 school ) OR pediatric OR child* OR perinatal OR kindergarten OR parent* ) ) )

AND ( ( ( TITLE-ABS-KEY ( health W/3 intervention* ) OR TITLE-ABS-KEY ( health W/3 prevention ) OR TITLE-ABS-KEY ( health W/3 policy ) OR TITLE-ABS-KEY ( health W/3 policies ) OR TITLE-ABS-KEY ( health W/3 program* ) OR TITLE-ABS-KEY ( health W/3 project* ) ) ) OR ( KEY ( "Public Policy" ) OR KEY ( "Organizational Policy" ) OR KEY ( "Fiscal Policy" ) OR KEY ( "Policy" ) OR KEY ( "Health Policy" ) OR KEY ( "Programs" ) OR KEY ( "Sewage" ) OR KEY ( "Bathroom Equipment" ) OR KEY ( "Water" ) OR KEY ( "Hygiene" ) OR KEY ( "Waste Management" ) OR KEY ( "Nutrition Therapy" ) OR KEY ( "Nutrition Policy" ) OR KEY ( "Health Education" ) OR KEY ( "Counseling" ) OR KEY ( "Health Knowledge, Attitudes, Practice" ) OR KEY ( "immunization" ) OR KEY ( "Immunization Programs" ) OR KEY ( "Community Health Aides" ) OR KEY ( "Social Support" ) OR KEY ( "Financing, Organized" ) OR KEY ( "maternal welfare" ) OR KEY ( "public policy" ) OR KEY ( "social welfare" ) OR KEY ( "social security" ) OR KEY ( "Environmental Health" ) OR KEY ( "Environmental Policy" ) ) OR ( ( ( TITLE-ABS-KEY ( prevent* W/3 transmission ) OR TITLE-ABS-KEY ( psychosocial W/3 support ) OR TITLE-ABS-KEY ( social W/3 support ) OR TITLE-ABS-KEY ( finance* W/3 transfer ) OR TITLE-ABS-KEY ( cash W/3 transfer ) OR TITLE-ABS-KEY ( money W/3 transfer ) OR TITLE-ABS-KEY ( environment* W/3 prevent* ) OR TITLE-ABS-KEY ( environment* W/3 control* ) ) ) OR ( TITLE-ABS-KEY ( "communicable diseases" OR "infectious diseases" OR "health knowledge" OR "health attitude" OR "health practice" OR water OR sanitation OR waste OR hygiene OR latrine OR toilets OR "waste disposal" OR sewage OR "hand washing" OR "nutrition policy" OR "food policy" OR breastfeeding OR "nutrition therapy" OR supplement OR "parenting program" OR counseling OR "health education" OR instruction OR immunisation OR immunization OR vaccine OR immun* OR inoculation OR insecticides OR "mosquito control" OR "bed nets" OR "mosquito nets" OR "vector control" OR "Prevention of mother-to-child transmission" OR pmtct OR deworming OR "community health" OR outreach OR saving OR voucher OR microfinance OR microcredit OR welfare OR "public assistance" OR "social security" OR insurance OR "family policy" OR "family policies" ) ) OR ( ( TITLE-ABS-KEY ( "health intervention" OR "public health" OR "health promotion" OR prevention OR protection OR "preventive health services" OR preventive OR control OR "child health" OR "child welfare" OR "child care" OR policy OR program OR project ) OR TITLE-ABS-KEY ( "Parasitic Diseases" OR finance* OR welfare OR environment* ) OR TITLE-ABS-KEY ( "Psychosocial Support System" ) ) ) ) )

AND ( ( ( ( KEY ( "Systematic Review" ) OR KEY ( "Meta Analysis" ) OR KEY ( "Meta-Analysis as Topic" ) OR KEY ( "Meta-Analysis" ) OR KEY ( "Review Literature as Topic" ) ) ) OR ( ( TITLE-ABS-KEY ( "meta analysis" OR "metaanalysis" OR "Meta-Analysis" OR "systematic review" OR overview* OR "evidence synthesis" OR "review of reviews" ) OR TITLE-ABS-KEY ( systematic W/2 review* ) OR TITLE-ABS-KEY ( systematic W/2 overview* ) OR TITLE-ABS-KEY ( umbrella W/2 review ) ) ) OR ( ( ABS ( cochrane OR embase OR psychlit OR psyclit ) OR ABS ( cinahl OR cinhal ) OR ABS ( psychinfo OR psycinfo ) OR ABS ( "science citation index" OR bids OR cancerlit ) ) ) OR ( ( ABS ( "reference list" OR "reference lists" OR bibliography* OR hand-search* OR "relevant journals" OR "manual search" OR "manual searches" ) OR ABS ( "selection criteria" OR "data extraction" ) ) ) ) AND NOT ( TITLE-ABS-KEY ( comment OR letter OR editorial OR animal ) ) )

AND ( ( TITLE-ABS-KEY ( "population health" OR "global health" OR "outcome assessment" ) OR TITLE-ABS-KEY ( effectiv* OR impact OR effect OR "outcome assessment" OR morbidity OR mortality OR death* OR disease* OR illness OR coverage ) OR TITLE-ABS-KEY ( reported W/3 cases ) OR TITLE-ABS-KEY ( cases W/3 averted ) ) )

AND ( PUBYEAR > 2000 )

AND ( EXCLUDE ( DOCTYPE , "sh" ) OR EXCLUDE ( DOCTYPE , "no" ) OR EXCLUDE ( DOCTYPE , "er" ) OR EXCLUDE ( DOCTYPE , "ed" ) OR EXCLUDE ( DOCTYPE , "cp" ) OR EXCLUDE ( DOCTYPE , "cr" ) )

Updated search

**Number of hits** (as of 05/01/2021): 1, 066

**Search string**:

( ( ( ( TITLE-ABS-KEY ( low* W/2 gdp ) OR TITLE-ABS-KEY ( low* W/2 gnp ) OR TITLE-ABS-KEY ( low* W/2 "gross domestic" ) OR TITLE-ABS-KEY ( low* W/2 "gross national" ) ) ) OR ( ( TITLE-ABS-KEY ( africa OR asia OR caribbean OR "West Indies" OR "South America" OR "Middle East" OR "Latin America" OR "Central America" ) ) OR ( TITLE-ABS-KEY ( "developing countries" OR lmic OR lmics OR "third world" OR "lami countries" OR "global south" OR transition* ) ) OR ( ( TITLE-ABS-KEY ( developing W/2 countr* ) OR TITLE-ABS-KEY ( "less developed" W/2 countr* ) OR TITLE-ABS-KEY ( "under developed" W/2 countr* ) OR TITLE-ABS-KEY ( underdeveloped W/2 countr* ) OR TITLE-ABS-KEY ( "middle income" W/2 countr* ) OR TITLE-ABS-KEY ( "low income" W/2 countr* ) OR TITLE-ABS-KEY ( "lower income" W/2 countr* ) OR TITLE-ABS-KEY ( underserved W/2 countr* ) OR TITLE-ABS-KEY ( "under served" W/2 countr* ) OR TITLE-ABS-KEY ( deprived W/2 countr* ) OR TITLE-ABS-KEY ( poor W/2 countr* ) ) ) ) )

AND ( TITLE-ABS-KEY ( infant OR newborn OR neonate OR baby OR babies OR toddler OR ( pre PRE/0 school ) OR pediatric OR child* OR perinatal OR kindergarten OR parent* ) ) )

AND ( ( ( TITLE-ABS-KEY ( health W/3 intervention* ) OR TITLE-ABS-KEY ( health W/3 prevention ) OR TITLE-ABS-KEY ( health W/3 policy ) OR TITLE-ABS-KEY ( health W/3 policies ) OR TITLE-ABS-KEY ( health W/3 program* ) OR TITLE-ABS-KEY ( health W/3 project* ) ) ) OR ( KEY ( "Public Policy" ) OR KEY ( "Organizational Policy" ) OR KEY ( "Fiscal Policy" ) OR KEY ( "Policy" ) OR KEY ( "Health Policy" ) OR KEY ( "Programs" ) OR KEY ( "Sewage" ) OR KEY ( "Bathroom Equipment" ) OR KEY ( "Water" ) OR KEY ( "Hygiene" ) OR KEY ( "Waste Management" ) OR KEY ( "Nutrition Therapy" ) OR KEY ( "Nutrition Policy" ) OR KEY ( "Health Education" ) OR KEY ( "Counseling" ) OR KEY ( "Health Knowledge, Attitudes, Practice" ) OR KEY ( "immunization" ) OR KEY ( "Immunization Programs" ) OR KEY ( "Community Health Aides" ) OR KEY ( "Social Support" ) OR KEY ( "Financing, Organized" ) OR KEY ( "maternal welfare" ) OR KEY ( "public policy" ) OR KEY ( "social welfare" ) OR KEY ( "social security" ) OR KEY ( "Environmental Health" ) OR KEY ( "Environmental Policy" ) ) OR ( ( ( TITLE-ABS-KEY ( prevent* W/3 transmission ) OR TITLE-ABS-KEY ( psychosocial W/3 support ) OR TITLE-ABS-KEY ( social W/3 support ) OR TITLE-ABS-KEY ( finance* W/3 transfer ) OR TITLE-ABS-KEY ( cash W/3 transfer ) OR TITLE-ABS-KEY ( money W/3 transfer ) OR TITLE-ABS-KEY ( environment* W/3 prevent* ) OR TITLE-ABS-KEY ( environment* W/3 control* ) ) ) OR ( TITLE-ABS-KEY ( "communicable diseases" OR "infectious diseases" OR "health knowledge" OR "health attitude" OR "health practice" OR water OR sanitation OR waste OR hygiene OR latrine OR toilets OR "waste disposal" OR sewage OR "hand washing" OR "nutrition policy" OR "food policy" OR breastfeeding OR "nutrition therapy" OR supplement OR "parenting program" OR counseling OR "health education" OR instruction OR immunisation OR immunization OR vaccine OR immun* OR inoculation OR insecticides OR "mosquito control" OR "bed nets" OR "mosquito nets" OR "vector control" OR "Prevention of mother-to-child transmission" OR pmtct OR deworming OR "community health" OR outreach OR saving OR voucher OR microfinance OR microcredit OR welfare OR "public assistance" OR "social security" OR insurance OR "family policy" OR "family policies" ) ) OR ( ( TITLE-ABS-KEY ( "health intervention" OR "public health" OR "health promotion" OR prevention OR protection OR "preventive health services" OR preventive OR control OR "child health" OR "child welfare" OR "child care" OR policy OR program OR project ) OR TITLE-ABS-KEY ( "Parasitic Diseases" OR finance* OR welfare OR environment* ) OR TITLE-ABS-KEY ( "Psychosocial Support System" ) ) ) ) )

AND ( ( ( ( KEY ( "Systematic Review" ) OR KEY ( "Meta Analysis" ) OR KEY ( "Meta-Analysis as Topic" ) OR KEY ( "Meta-Analysis" ) OR KEY ( "Review Literature as Topic" ) ) ) OR ( ( TITLE-ABS-KEY ( "meta analysis" OR "metaanalysis" OR "Meta-Analysis" OR "systematic review" OR overview* OR "evidence synthesis" OR "review of reviews" ) OR TITLE-ABS-KEY ( systematic W/2 review* ) OR TITLE-ABS-KEY ( systematic W/2 overview* ) OR TITLE-ABS-KEY ( umbrella W/2 review ) ) ) OR ( ( ABS ( cochrane OR embase OR psychlit OR psyclit ) OR ABS ( cinahl OR cinhal ) OR ABS ( psychinfo OR psycinfo ) OR ABS ( "science citation index" OR bids OR cancerlit ) ) ) OR ( ( ABS ( "reference list" OR "reference lists" OR bibliography* OR hand-search* OR "relevant journals" OR "manual search" OR "manual searches" ) OR ABS ( "selection criteria" OR "data extraction" ) ) ) ) AND NOT ( TITLE-ABS-KEY ( comment OR letter OR editorial OR animal ) ) )

AND ( ( TITLE-ABS-KEY ( "population health" OR "global health" OR "outcome assessment" ) OR TITLE-ABS-KEY ( effectiv* OR impact OR effect OR "outcome assessment" OR morbidity OR mortality OR death* OR disease* OR illness OR coverage ) OR TITLE-ABS-KEY ( reported W/3 cases ) OR TITLE-ABS-KEY ( cases W/3 averted ) ) )

AND ( EXCLUDE ( DOCTYPE , "sh" ) OR EXCLUDE ( DOCTYPE , "no" ) OR EXCLUDE ( DOCTYPE , "er" ) OR EXCLUDE ( DOCTYPE , "ed" ) OR EXCLUDE ( DOCTYPE , "cp" ) OR EXCLUDE ( DOCTYPE , "cr" ) )

AND ( LIMIT-TO ( PUBYEAR , 2021 ) OR LIMIT-TO ( PUBYEAR , 2020 ) OR LIMIT-TO ( PUBYEAR , 2019 ) )

Search string in EMBASE

Ovid Embase 1974 to present

Initial search

**Number of hits** (as of 19/06/2019): **2,706**

**Search string**:

| Type of study | 1 | exp meta analysis/ |
| --- | --- | --- |
|  | 2 | ((meta adj analy$) or metaanalys$).tw. |
|  | 3 | (systematic adj (review$1 or overview$1)).tw. |
|  | 4 | (cochrane or embase or (psychlit or psyclit) or (cinahl or cinhal) or (psychinfo or psycinfo) or "science citation index" or bids or cancerlit).ab. |
|  | 5 | ("reference list*" or bibliograph* or hand-search* or "relevant journals" or "manual search*").ab. |
|  | 6 | ("selection criteria" or "data extraction").ab. |
|  | 7 | "evidence synthes*".ti,ab. |
|  | 8 | ((systematic adj2 (review* or overview*)) or (umbrella adj2 review) or "review of reviews" or (systematic adj (review$1 or overview$1))).ti,ab. |
|  | 9 | 1 or 2 or 3 or 4 or 5 or 6 or 7 or 8 |
|  | 10 | note.pt. |
|  | 11 | (Comment or Letter or Editorial or animal or "Published Erratum").hw. |
|  | 12 | "conference paper".ti,ab. |
|  | 13 | (Surveys and Questionnaires).hw. |
|  | 14 | 10 or 11 or 12 or 13 |
|  | 15 | 9 not 14 |
| Population | 16 | (Africa or Asia or Caribbean or "West Indies" or "South America" or "Middle East" or "Latin America" or "Central America").ab,hw,kw,ti. |
|  | 17 | ("developing countries" or lmic or lmics or "third world" or "lami countries" or "global south" or transition*).ab,hw,kw,ti. |
|  | 18 | ((developing or "less developed" or "under developed" or underdeveloped or "middle income" or "low income" or "lower income" or underserved or "under served" or deprived or poor) adj countr*).ab,hw,kw,ti. |
|  | 19 | (low* adj (gdp or gnp or "gross domestic" or "gross national")).ab,hw,kw,ti. |
|  | 20 | 16 or 17 or 18 or 19 |
|  | 21 | exp infant/ |
|  | 22 | exp newborn/ |
|  | 23 | exp child/ |
|  | 24 | exp parent/ |
|  | 25 | exp preschool child/ |
|  | 26 | 21 or 22 or 23 or 24 or 25 |
|  | 27 | (infant or newborn or neonate or baby or babies or toddler or pre-school or pediatric or child* or perinatal or kindergarten or parent*).ab,hw,kw,ti. |
|  | 28 | 26 or 27 |
|  | 29 | 20 and 28 |
| Intervention | 30 | exp child care/ |
|  | 31 | exp child welfare/ |
|  | 32 | exp child health/ |
|  | 33 | exp communicable disease control/ or exp communicable disease/ or exp public health service/ or exp public health/ |
|  | 34 | exp health promotion/ |
|  | 35 | exp primary prevention/ |
|  | 36 | exp preventive health service/ |
|  | 37 | exp health care delivery/ |
|  | 38 | exp health care delivery/ |
|  | 39 | 30 or 31 or 32 or 33 or 34 or 35 or 36 or 37 or 38 |
|  | 40 | ("health intervention" or "public health" or "health promotion" or prevention or protection or "preventive health services" or preventive or control or "child health" or "child welfare" or "child care" or policy or program or project).ab,hw,kw,ti. |
|  | 41 | (health adj8 (intervention* or prevention or policy or policies or program* or project*)).ti,ab. |
|  | 42 | exp public policy/ or exp policy/ |
|  | 43 | exp organizational policy/ or exp public policy/ or exp environmental policy/ or exp nutrition policy/ or exp fiscal policy/ |
|  | 44 | exp health program/ |
|  | 45 | exp sewage/ |
|  | 46 | exp bathroom equipment/ |
|  | 47 | exp water/ |
|  | 48 | exp hygiene/ |
|  | 49 | exp waste management/ |
|  | 50 | exp nutrition service/ |
|  | 51 | exp health education/ |
|  | 52 | exp counseling/ |
|  | 53 | exp attitude to health/ |
|  | 54 | exp immunization/ |
|  | 55 | exp social support/ or exp social welfare/ or exp social security/ |
|  | 56 | exp financial management/ |
|  | 57 | exp maternal welfare/ |
|  | 58 | exp environmental health/ |
|  | 59 | 42 or 43 or 44 or 45 or 46 or 47 or 48 or 49 or 50 or 51 or 52 or 53 or 54 or 55 or 56 or 57 or 58 |
|  | 60 | (Communicable disease* or infectious disease* or water or sanitation or hygiene or health knowledge or Health attitude or practice or immunization or Parasitic Diseases or Psychosocial Support Systems or water or sanitation or latrine or toilets or waste disposal or sewage or hygiene or hand wash* or nutrition policy or food policy or breastfeeding or nutrition therapy or supplement or parenting program or counseling or health education or instruction or immuni#ation or vaccine or immune* or inoculation or insecticides or mosquito control or bed nets or mosquito nets or vector control or Prevention of mother-to-child transmission or PMTCT or deworming or community health or outreach or saving* or voucher or microfinance or microcredit or Welfare or public assistance or social security or insurance or family policy or environment*).ti,ab. |
|  | 61 | ((psychosocial adj3 support) or (social adj3 support) or (prevent* adj3 transmission) or ((finance* or cash or money) adj3 transfer) or (environment adj3 (prevent* or control*))).ti,ab. |
|  | 62 | 39 or 40 or 41 or 59 or 60 or 61 |
| P+I+S | 63 | 15 and 29 and 62 |
| Outcomes | 64 | exp population health/ |
|  | 65 | exp global health/ |
|  | 66 | exp outcome assessment/ |
|  | 67 | (population health or global health or outcome assessment).ab,hw,kw,ti. |
|  | 68 | 64 or 65 or 66 |
|  | 69 | 67 or 68 |
|  | 70 | (effective* or impact or effect or outcome assessment or morbidity or mortality or death* or disease* or illness or coverage or (reported adj3 cases) or (cases adj3 averted)).ti,ab. |
|  | 71 | 69 or 70 |
| P+I+O+S | 72 | 63 and 71 |
| Year | 73 | limit 72 to yr="2000 -Current" |

Updated search

**Number of hits** (as of 05/01/2021): **835**

**Search string**:

| Type of study | 1 | exp meta analysis/ |
| --- | --- | --- |
|  | 2 | ((meta adj analy$) or metaanalys$).tw. |
|  | 3 | (systematic adj (review$1 or overview$1)).tw. |
|  | 4 | (cochrane or embase or (psychlit or psyclit) or (cinahl or cinhal) or (psychinfo or psycinfo) or "science citation index" or bids or cancerlit).ab. |
|  | 5 | ("reference list*" or bibliograph* or hand-search* or "relevant journals" or "manual search*").ab. |
|  | 6 | ("selection criteria" or "data extraction").ab. |
|  | 7 | "evidence synthes*".ti,ab. |
|  | 8 | ((systematic adj2 (review* or overview*)) or (umbrella adj2 review) or "review of reviews" or (systematic adj (review$1 or overview$1))).ti,ab. |
|  | 9 | 1 or 2 or 3 or 4 or 5 or 6 or 7 or 8 |
|  | 10 | note.pt. |
|  | 11 | (Comment or Letter or Editorial or animal or "Published Erratum").hw. |
|  | 12 | "conference paper".ti,ab. |
|  | 13 | (Surveys and Questionnaires).hw. |
|  | 14 | 10 or 11 or 12 or 13 |
|  | 15 | 9 not 14 |
| Population | 16 | (Africa or Asia or Caribbean or "West Indies" or "South America" or "Middle East" or "Latin America" or "Central America").ab,hw,kw,ti. |
|  | 17 | ("developing countries" or lmic or lmics or "third world" or "lami countries" or "global south" or transition*).ab,hw,kw,ti. |
|  | 18 | ((developing or "less developed" or "under developed" or underdeveloped or "middle income" or "low income" or "lower income" or underserved or "under served" or deprived or poor) adj countr*).ab,hw,kw,ti. |
|  | 19 | (low* adj (gdp or gnp or "gross domestic" or "gross national")).ab,hw,kw,ti. |
|  | 20 | 16 or 17 or 18 or 19 |
|  | 21 | exp infant/ |
|  | 22 | exp newborn/ |
|  | 23 | exp child/ |
|  | 24 | exp parent/ |
|  | 25 | exp preschool child/ |
|  | 26 | 21 or 22 or 23 or 24 or 25 |
|  | 27 | (infant or newborn or neonate or baby or babies or toddler or pre-school or pediatric or child* or perinatal or kindergarten or parent*).ab,hw,kw,ti. |
|  | 28 | 26 or 27 |
|  | 29 | 20 and 28 |
| Intervention | 30 | exp child care/ |
|  | 31 | exp child welfare/ |
|  | 32 | exp child health/ |
|  | 33 | exp communicable disease control/ or exp communicable disease/ or exp public health service/ or exp public health/ |
|  | 34 | exp health promotion/ |
|  | 35 | exp primary prevention/ |
|  | 36 | exp preventive health service/ |
|  | 37 | exp health care delivery/ |
|  | 38 | exp health care delivery/ |
|  | 39 | 30 or 31 or 32 or 33 or 34 or 35 or 36 or 37 or 38 |
|  | 40 | ("health intervention" or "public health" or "health promotion" or prevention or protection or "preventive health services" or preventive or control or "child health" or "child welfare" or "child care" or policy or program or project).ab,hw,kw,ti. |
|  | 41 | (health adj8 (intervention* or prevention or policy or policies or program* or project*)).ti,ab. |
|  | 42 | exp public policy/ or exp policy/ |
|  | 43 | exp organizational policy/ or exp public policy/ or exp environmental policy/ or exp nutrition policy/ or exp fiscal policy/ |
|  | 44 | exp health program/ |
|  | 45 | exp sewage/ |
|  | 46 | exp bathroom equipment/ |
|  | 47 | exp water/ |
|  | 48 | exp hygiene/ |
|  | 49 | exp waste management/ |
|  | 50 | exp nutrition service/ |
|  | 51 | exp health education/ |
|  | 52 | exp counseling/ |
|  | 53 | exp attitude to health/ |
|  | 54 | exp immunization/ |
|  | 55 | exp social support/ or exp social welfare/ or exp social security/ |
|  | 56 | exp financial management/ |
|  | 57 | exp maternal welfare/ |
|  | 58 | exp environmental health/ |
|  | 59 | 42 or 43 or 44 or 45 or 46 or 47 or 48 or 49 or 50 or 51 or 52 or 53 or 54 or 55 or 56 or 57 or 58 |
|  | 60 | (Communicable disease* or infectious disease* or water or sanitation or hygiene or health knowledge or Health attitude or practice or immunization or Parasitic Diseases or Psychosocial Support Systems or water or sanitation or latrine or toilets or waste disposal or sewage or hygiene or hand wash* or nutrition policy or food policy or breastfeeding or nutrition therapy or supplement or parenting program or counseling or health education or instruction or immuni#ation or vaccine or immune* or inoculation or insecticides or mosquito control or bed nets or mosquito nets or vector control or Prevention of mother-to-child transmission or PMTCT or deworming or community health or outreach or saving* or voucher or microfinance or microcredit or Welfare or public assistance or social security or insurance or family policy or environment*).ti,ab. |
|  | 61 | ((psychosocial adj3 support) or (social adj3 support) or (prevent* adj3 transmission) or ((finance* or cash or money) adj3 transfer) or (environment adj3 (prevent* or control*))).ti,ab. |
|  | 62 | 39 or 40 or 41 or 59 or 60 or 61 |
| P+I+S | 63 | 15 and 29 and 62 |
| Outcomes | 64 | exp population health/ |
|  | 65 | exp global health/ |
|  | 66 | exp outcome assessment/ |
|  | 67 | (population health or global health or outcome assessment).ab,hw,kw,ti. |
|  | 68 | 64 or 65 or 66 |
|  | 69 | 67 or 68 |
|  | 70 | (effective* or impact or effect or outcome assessment or morbidity or mortality or death* or disease* or illness or coverage or (reported adj3 cases) or (cases adj3 averted)).ti,ab. |
|  | 71 | 69 or 70 |
| P+I+O+S | 72 | 63 and 71 |
| Year | 73 | limit 72 to yr="2000 -Current" |
|  | 74 | limit 72 to yr="2019 -Current" |

Search string in Web of Science - Social Sciences Citation Index

Web of Science : Social Sciences Citation Index (SSCI) --1956-present

Initial search

**Number of hits** (as of 21/06/2019): **1,421**

**Search string**:

| #1 | TOPIC: (low* NEAR/2 gdp) OR TOPIC: (low* NEAR/2 gnp) OR TOPIC: (low* NEAR/2 "gross domestic") OR TOPIC: (low* NEAR/2 "gross national") OR TOPIC: (africa OR asia OR caribbean OR "West Indies" OR "South America" OR "Middle East" OR "Latin America" OR "Central America") OR TOPIC: ("developing countries" OR lmic OR lmics OR "third world" OR "lami countries" OR "global south" OR transition*) OR TOPIC: (developing NEAR/2 countr*) OR TOPIC: ("less developed" NEAR/2 countr*) OR TOPIC: ("under developed" NEAR/2 countr*) OR TOPIC: (underdeveloped NEAR/2 countr*) OR TOPIC: ("middle income" NEAR/2 countr*) OR TOPIC: ("low income" NEAR/2 countr*) OR TOPIC: ("lower income" NEAR/2 countr*) OR TOPIC: (underserved NEAR/2 countr*) OR TOPIC: ("under served" NEAR/2 countr*) OR TOPIC: (deprived NEAR/2 countr*) OR TOPIC: (poor NEAR/2 countr*)  Indexes=SSCI Timespan=1945-2019 |
| --- | --- |
| #2 | TOPIC: (pre NEAR/0 school) OR TOPIC: (infant OR newborn OR neonate OR baby OR babies OR toddler OR pediatric OR child* OR perinatal OR kindergarten OR parent*)  Indexes=SSCI Timespan=1945-2019 |
| #3 | TOPIC: (( health NEAR/3 intervention* ) OR ( health NEAR/3 prevention ) OR ( health NEAR/3 policy ) OR ( health NEAR/3 policies ) OR ( health NEAR/3 program* ) OR ( health NEAR/3 project* )) OR TOPIC: ("Public Policy" OR "Organizational Policy" OR "Fiscal Policy" OR "Health Policy" OR Program* OR "Bathroom Equipment" OR “Waste Management" OR Counseling OR immunization OR immune* OR vaccine OR "Immunization Programs" OR "Social Support" OR "Financing, Organized" OR "maternal welfare" OR "social welfare" OR “Environmental Health" OR "Environmental Policy" OR "communicable diseases" OR "infectious diseases" OR "health knowledge" OR "health attitude" OR "health practice" OR water OR sanitation OR waste OR hygiene OR latrine OR toilets OR "waste disposal" OR sewage OR "hand washing" OR "nutrition policy" OR "food policy" OR breastfeeding OR "nutrition therapy" OR supplement OR "parenting program" OR counseling OR "health education" OR instruction OR inoculation OR insecticides OR "mosquito control" OR "bed nets" OR "mosquito nets" OR "vector control" OR "Prevention of mother-to-child transmission" OR pmtct OR deworming OR "community health" OR outreach OR saving OR voucher OR microfinance OR microcredit OR welfare OR "public assistance" OR "social security" OR insurance OR "family policy" OR "family policies" OR "health intervention" OR "public health" OR "health promotion" OR prevention OR protection OR "preventive health services" OR preventive OR control OR "child health" OR "child welfare" OR "child care" OR policy OR program OR project OR "Parasitic Diseases" OR finance* OR welfare OR environment* OR "Psychosocial Support System") OR TOPIC: (( prevent* NEAR/3 transmission ) OR ( psychosocial NEAR/3 support ) OR ( social NEAR/3 support ) OR ( finance* NEAR/3 transfer ) OR ( cash NEAR/3 transfer ) OR ( money NEAR/3 transfer ) OR ( environment* NEAR/3 prevent* ) OR ( environment* NEAR/3 control* ))  Indexes=SSCI Timespan=1945-2019 |
| #4 | TOPIC: ("Systematic Review" OR "Meta Analysis" OR "Meta-Analysis as Topic" OR "Review Literature as Topic" OR meta*analysis OR overview* OR "evidence synthesis" OR "review of reviews") OR TOPIC: ((systematic NEAR/2 review* ) OR ( systematic NEAR/2 overview* ) OR ( umbrella NEAR/2 review )) OR TOPIC: (cochrane OR embase OR psyc*lit OR cinahl OR cinhal OR psyc*info OR "science citation index" OR bids OR cancerlit OR “reference list" OR "reference lists" OR bibliography* OR hand-search* OR "relevant journals" OR "manual search" OR "manual searches" OR "selection criteria" OR "data extraction") NOT TOPIC: (Editorial or Letter or Comment or Erratum or Conference paper or Survey or Note)  Indexes=SSCI Timespan=1945-2019 |
| #5 | TOPIC: ("population health" OR "global health" OR "outcome assessment" OR effectiv* OR impact OR effect OR "outcome assessment" OR morbidity OR mortality OR death* OR disease* OR illness OR coverage) OR TOPIC: (( reported NEAR/3 cases ) OR ( cases NEAR/3 averted ))  Indexes=SSCI Timespan=1945-2019 |
| #6 | #2 AND #1  Indexes=SSCI Timespan=1945-2019 |
| #7 | #6 AND #5 AND #4 AND #3  Indexes=SSCI Timespan=1945-2019 |
| #8 | #6 AND #5 AND #4 AND #3  Refined by: PUBLICATION YEARS: ( 2019 OR 2012 OR 2005 OR 2018 OR 2011 OR 2004 OR 2017 OR 2010 OR 2003 OR 2016 OR 2009 OR 2002 OR 2015 OR 2008 OR 2001 OR 2014 OR 2007 OR 2000 OR 2013 OR 2006 )  Indexes=SSCI Timespan=1945-2019 |
| #9 | #6 AND #5 AND #3 AND #2  Refined by: PUBLICATION YEARS: ( 2019 OR 2012 OR 2005 OR 2018 OR 2011 OR 2004 OR 2017 OR 2010 OR 2003 OR 2016 OR 2009 OR 2002 OR 2015 OR 2008 OR 2001 OR 2014 OR 2007 OR 2000 OR 2013 OR 2006 ) AND [excluding] DOCUMENT TYPES: ( EDITORIAL MATERIAL OR PROCEEDINGS PAPER )  Indexes=SSCI Timespan=1945-2019 |

Updated search

**Number of hits** (as of 05/01/2021): **461**

**Search string**:

| # 1 | **TOPIC:**  (low* NEAR/2 gdp)  *OR*  **TOPIC:**  (low* NEAR/2 gnp)  *OR*  **TOPIC:**  (low* NEAR/2 "gross domestic")  *OR*  **TOPIC:**  (low* NEAR/2 "gross national")  *OR*  **TOPIC:**  (africa OR asia OR caribbean OR "West Indies" OR "South America" OR "Middle East" OR "Latin America" OR "Central America")  *OR*  **TOPIC:**  ("developing countries" OR lmic OR lmics OR "third world" OR "lami countries" OR "global south" OR transition*)  *OR*  **TOPIC:**  (developing NEAR/2 countr*)  *OR*  **TOPIC:**  ("less developed" NEAR/2 countr*)  *OR*  **TOPIC:**  ("under developed" NEAR/2 countr*)  *OR*  **TOPIC:**  (underdeveloped NEAR/2 countr*)  *OR*  **TOPIC:**  ("middle income" NEAR/2 countr*)  *OR*  **TOPIC:**  ("low income" NEAR/2 countr*)  *OR*  **TOPIC:**  ("lower income" NEAR/2 countr*)  *OR*  **TOPIC:**  (underserved NEAR/2 countr*)  *OR*  **TOPIC:**  ("under served" NEAR/2 countr*)  *OR*  **TOPIC:**  (deprived NEAR/2 countr*)  *OR*  **TOPIC:**  (poor NEAR/2 countr*)  Indexes=SSCI Timespan=All years |
| --- | --- |
| # 2 | **TOPIC:**  (( health NEAR/3 intervention* )  OR  ( health NEAR/3 prevention )  OR  ( health NEAR/3 policy )  OR  ( health NEAR/3 policies )  OR  ( health NEAR/3 program* )  OR  ( health NEAR/3 project* ) )  *OR*  **TOPIC:**  ("Public Policy" OR "Organizational Policy" OR "Fiscal Policy" OR "Health Policy" OR Program* OR "Bathroom Equipment" OR “Waste Management" OR Counseling OR immunization OR immune* OR vaccine OR "Immunization Programs" OR "Social Support" OR "Financing, Organized" OR "maternal welfare" OR "social welfare" OR “Environmental Health" OR "Environmental Policy" OR "communicable diseases" OR "infectious diseases" OR "health knowledge" OR "health attitude" OR "health practice" OR water OR sanitation OR waste OR hygiene OR latrine OR toilets OR "waste disposal" OR sewage OR "hand washing" OR "nutrition policy" OR "food policy" OR breastfeeding OR "nutrition therapy" OR supplement OR "parenting program" OR counseling OR "health education" OR instruction OR inoculation OR insecticides OR "mosquito control" OR "bed nets" OR "mosquito nets" OR "vector control" OR "Prevention of mother-to-child transmission" OR pmtct OR deworming OR "community health" OR outreach OR saving OR voucher OR microfinance OR microcredit OR welfare OR "public assistance" OR "social security" OR insurance OR "family policy" OR "family policies" OR "health intervention" OR "public health" OR "health promotion" OR prevention OR protection OR "preventive health services" OR preventive OR control OR "child health" OR "child welfare" OR "child care" OR policy OR program OR project OR "Parasitic Diseases" OR finance* OR welfare OR environment* OR "Psychosocial Support System")  *OR*  **TOPIC:**  (( prevent* NEAR/3 transmission )  OR  ( psychosocial NEAR/3 support )  OR  ( social NEAR/3 support )  OR  ( finance* NEAR/3 transfer )  OR  ( cash NEAR/3 transfer )  OR  ( money NEAR/3 transfer )  OR  ( environment* NEAR/3 prevent* )  OR  ( environment* NEAR/3 control* ) )  Indexes=SSCI Timespan=All years |
| # 3 | **TOPIC:**  ("population health" OR "global health" OR "outcome assessment" OR effectiv* OR impact OR effect OR "outcome assessment" OR morbidity OR mortality OR death* OR disease* OR illness OR coverage)  *OR*  **TOPIC:**  (( reported NEAR/3 cases )  OR  ( cases NEAR/3 averted ) )  Indexes=SSCI Timespan=All years |
| # 4 | **TOPIC:**  (pre NEAR/0 school)  *OR*  **TOPIC:**  (infant OR newborn OR neonate OR baby OR babies OR toddler OR pediatric OR child* OR perinatal OR kindergarten OR parent*)  Indexes=SSCI Timespan=All years |
| # 5 | **TOPIC:**  ("Systematic Review" OR "Meta Analysis" OR "Meta-Analysis as Topic" OR "Review Literature as Topic" OR meta*analysis OR overview* OR "evidence synthesis" OR "review of reviews")  *OR*  **TOPIC:**  ((systematic NEAR/2 review* )  OR  ( systematic NEAR/2 overview* )  OR  ( umbrella NEAR/2 review ) )  *OR*  **TOPIC:**  (cochrane OR embase OR psyc*lit OR cinahl OR cinhal OR psyc*info OR "science citation index" OR bids OR cancerlit OR “reference list" OR "reference lists" OR bibliography* OR hand-search* OR "relevant journals" OR "manual search" OR "manual searches" OR "selection criteria" OR "data extraction")  *NOT*  **TOPIC:**  (Editorial or Letter or Comment or Erratum or Conference paper or Survey or Note)  Indexes=SSCI Timespan=All years |
| # 6 | #4  AND  #1  Indexes=SSCI Timespan=All years |
| # 7 | #6  AND  #5  AND  #3  AND  #2  Indexes=SSCI Timespan=All years |
| # 8 | #6  AND  #5  AND  #3  AND  #2  **Refined by:** **PUBLICATION YEARS:** ( 2019 OR 2012 OR 2005 OR 2018 OR 2011 OR 2004 OR 2017 OR 2010 OR 2003 OR 2016 OR 2009 OR 2002 OR 2015 OR 2008 OR 2001 OR 2014 OR 2007 OR 2000 OR 2013 OR 2006 )  Indexes=SSCI Timespan=All years |
| # 9 | #6  AND  #5  AND  #3  AND  #2  **Refined by:** **PUBLICATION YEARS:** ( 2019 OR 2012 OR 2005 OR 2018 OR 2011 OR 2004 OR 2017 OR 2010 OR 2003 OR 2016 OR 2009 OR 2002 OR 2015 OR 2008 OR 2001 OR 2014 OR 2007 OR 2000 OR 2013 OR 2006 ) AND [excluding] **DOCUMENT TYPES:** ( EDITORIAL MATERIAL OR PROCEEDINGS PAPER )  Indexes=SSCI Timespan=All years |
| # 10 | #6  AND  #5  AND  #3  AND  #2  **Refined by:** **PUBLICATION YEARS:** ( 2020 OR 2019 )  Indexes=SSCI Timespan=All years |
| # 11 | #6  AND  #5  AND  #3  AND  #2  **Refined by:** **PUBLICATION YEARS:** ( 2020 OR 2019 ) AND [excluding] **DOCUMENT TYPES:** ( EDITORIAL MATERIAL OR PROCEEDINGS PAPER )  Indexes=SSCI Timespan=All years |

Search string in the Cochrane Library

Cochrane Library: Cochrane Database of Systematic Reviews, the Cochrane Central Register of Controlled Trials and the Cochrane Clinical Answers with Cochrane Library publication date from Jan 2000 to present

Initial search

**Number of hits** (as of 19/06/2019): **559**

**Search string**:

| #1 | [mh "Systematic Review"] OR [mh "Meta Analysis"] OR [mh "Meta-Analysis as Topic"] OR [mh Meta-Analysis] OR [mh "Review Literature as Topic"] |
| --- | --- |
| #2 | ("meta analysis" OR metaanalysis OR (systematic NEAR (review OR overview))):ti,ab |
| #3 | (cochrane OR embase OR (psychlit OR psyclit) OR (cinahl OR cinhal) OR (psychinfo OR psycinfo) OR "science citation index" OR bids OR cancerlit):ab |
| #4 | ("reference list" OR bibliography OR bibliographies OR hand-search OR "relevant journals" OR "manual search" OR "manual searches"):ab |
| #5 | (("selection criteria" OR "data extraction"):ab) |
| #6 | ("evidence synthesis":ti,ab) |
| #7 | (((systematic NEAR/2 (review OR overview)) OR (umbrella NEAR/2 review) OR "review of reviews" OR (systematic NEAR (review OR overview))):ti,ab) |
| #8 | #1 OR #2 OR #3 OR #4 OR #5 OR #6 OR #7 |
| #9 | (Comment OR Letter OR Editorial OR animal OR "Published Erratum"):ti,ab,kw OR ("conference paper"):ti,ab,kw OR (Surveys AND Questionnaires):ti,ab,kw OR (note):pt |
| #10 | #8 NOT #9 |
| #11 | (Africa OR Asia OR Caribbean OR "West Indies" OR "South America" OR "Middle East" OR "Latin America" OR "Central America"):ti,ab,kw OR ("developing countries" OR lmic OR lmics OR "third world" OR "lami countries" OR "global south" OR transition*):ti,ab,kw OR ((developing OR "less developed" OR "under developed" OR underdeveloped OR "middle income" OR "low income" OR "lower income" OR underserved OR "under served" OR deprived OR poor) NEAR (country OR countries)):ti,ab,kw OR (low* NEAR (gdp OR gnp OR "gross domestic" OR "gross national")):pt |
| #12 | [mh "Infant, Newborn"] OR [mh Child] OR [mh Infant] OR [mh Parents] OR [mh "Child, Preschool"] |
| #13 | (infant OR newborn OR neonate OR baby OR babies OR toddler OR pre-school OR pediatric OR child* OR perinatal OR kindergarten OR parent*):ti,ab,kw |
| #14 | #12 OR #13 |
| #15 | #11 AND #14 |
| #16 | [mh "Child Care"] OR [mh ^"child welfare"] OR [mh "Child Health"] |
| #17 | [mh "Communicable Disease Control"] |
| #18 | [mh "Communicable Diseases"] OR [mh "Public Health"] OR [mh "Health Promotion"] OR [mh "Public Health"] OR [mh "PRIMARY PREVENTION"] OR [mh "Preventive Health Services"] OR [mh "DELIVERY OF HEALTH CARE"] |
| #19 | [mh "Public Policy"] OR [mh "Organizational Policy"] OR [mh "Fiscal Policy"] OR [mh Policy] OR [mh "Health Policy"] OR [mh Programs] OR [mh Sewage] OR [mh "Bathroom Equipment"] OR [mh Water] OR [mh Hygiene] OR [mh "Waste Management"] OR [mh "Nutrition Therapy"] OR [mh "Nutrition Policy"] OR [mh "Health Education"] OR [mh Counseling] OR [mh "Health Knowledge, Attitudes, Practice"] OR [mh immunization] OR [mh "Immunization Programs"] OR [mh "Community Health Aides"] OR [mh "Social Support"] OR [mh "Financing, Organized"] OR [mh "maternal welfare"] OR [mh "public policy"] OR [mh "social welfare"] OR [mh "social security"] OR [mh "Environmental Health"] OR [mh "Environmental Policy"] |
| #20 | ("health intervention" OR "public health" OR "health promotion" OR prevention OR protection OR "preventive health services" OR preventive OR control OR "child health" OR "child welfare" OR "child care" OR policy OR program OR project):ti,ab,kw OR (health NEAR/8 (intervention* OR prevention OR policy OR policies OR program* OR project*)):ti,ab,kw OR ("Communicable diseases" OR "infectious diseases" OR water OR sanitation OR hygiene OR "health knowledge" OR "Health attitude" OR practice OR immunization OR "Parasitic Diseases" OR "Psychosocial Support Systems" OR water OR sanitation OR latrine OR toilets OR "waste disposal" OR sewage OR hygiene OR "hand washing" OR "nutrition policy" OR "food policy" OR breastfeeding OR "nutrition therapy" OR supplement OR "parenting program" OR counseling OR "health education" OR instruction OR immunization OR immunization OR vaccine OR immune* OR inoculation OR insecticides OR "mosquito control" OR "bed nets" OR "mosquito nets" OR "vector control" OR "Prevention of mother-to-child transmission" OR PMTCT OR deworming OR "community health" OR outreach OR saving* OR voucher OR microfinance OR microcredit OR Welfare OR "public assistance" OR "social security" OR insurance OR "family policy" OR environment*):ti,ab,kw OR ((psychosocial NEAR/3 support) OR (social NEAR/3 support) OR (prevent* NEAR/3 transmission) OR ((finance* OR cash OR money) NEAR/3 transfer) OR (environment NEAR/3 (prevent* OR control*))):ti,ab,kw |
| #21 | #16 OR #17 OR #18 OR #19 |
| #22 | #20 OR #21 |
| #23 | #10 AND #15 AND #22 |
| #24 | [mh "Population Health"] OR [mh "Global Health"] OR [mh "Outcome Assessment (Health Care)"] |
| #25 | ("population health" OR "global health" OR "outcome assessment"):ti,ab,kw |
| #26 | #24 OR #25 |
| #27 | (effective* OR impact OR effect OR "outcome assessment" OR morbidity OR mortality OR death* OR disease* OR illness OR coverage OR (reported NEAR/3 cases) OR (cases NEAR/3 averted)):ti,ab,kw |
| #28 | #26 OR #27 |
| #29 | #23 AND #28 |

Updated search (modified interface)

**Number of hits** (as of 05/01/2021): **544**

**Search string**

Search Name: PH umbrella Jan 2021

Last Saved: 05/01/2021 21:29:36

Comment: Full search string PIOS

| #1 | [mh “Systematic Reviews as Topic”] OR [mh "Meta-Analysis as Topic"] OR [mh "Systematic Review"] OR [mh "Review Literature as Topic"] OR [mh "Review Literature"] OR [mh "Meta Analysis"] OR [mh "Meta-Analysis"] |
| --- | --- |
| #2 | ("meta analysis" OR metaanalysis OR (systematic NEAR (review OR overview))):ti,ab |
| #3 | (cochrane OR embase OR (psychlit OR psyclit) OR (cinahl OR cinhal) OR (psychinfo OR psycinfo) OR "science citation index" OR bids OR cancerlit):ab |
| #4 | ("reference list" OR bibliography OR bibliographies OR hand-search OR "relevant journals" OR "manual search" OR "manual searches"):ab |
| #5 | (("selection criteria" OR "data extraction"):ab) |
| #6 | ("evidence synthesis":ti,ab) |
| #7 | (((systematic NEAR/2 (review OR overview)) OR (umbrella NEAR/2 review) OR "review of reviews" OR (systematic NEAR (review OR overview))):ti,ab) |
| #8 | #1 OR #2 OR #3 OR #4 OR #5 OR #6 OR #7 |
| #9 | (Comment OR Letter OR Editorial OR animal OR "Published Erratum"):ti,ab,kw OR ("conference paper"):ti,ab,kw OR (Surveys AND Questionnaires):ti,ab,kw OR (note):pt |
| #10 | #8 NOT #9 |
| #11 | (Africa OR Asia OR Caribbean OR "West Indies" OR "South America" OR "Middle East" OR "Latin America" OR "Central America"):ti,ab,kw OR ("developing countries" OR lmic OR lmics OR "third world" OR "lami countries" OR "global south" OR transition*):ti,ab,kw OR ((developing OR "less developed" OR "under developed" OR underdeveloped OR "middle income" OR "low income" OR "lower income" OR underserved OR "under served" OR deprived OR poor) NEAR (country OR countries)):ti,ab,kw OR (low* NEAR (gdp OR gnp OR "gross domestic" OR "gross national")):pt |
| #12 | [mh "Infant, Newborn"] OR [mh Child] OR [mh Infant] OR [mh Parents] OR [mh "Child, Preschool"] |
| #13 | (infant OR newborn OR neonate OR baby OR babies OR toddler OR pre-school OR pediatric OR child* OR perinatal OR kindergarten OR parent*):ti,ab,kw |
| #14 | #12 OR #13 |
| #15 | #11 AND #14 |
| #16 | [mh "Child Care"] OR [mh ^"child welfare"] OR [mh "Child Health"] |
| #17 | [mh "Communicable Disease Control"] |
| #18 | [mh "Communicable Diseases"] OR [mh "Public Health"] OR [mh "Health Promotion"] OR [mh "Public Health"] OR [mh "PRIMARY PREVENTION"] OR [mh "Preventive Health Services"] OR [mh "DELIVERY OF HEALTH CARE"] |
| #19 | [mh "Public Policy"] OR [mh "Organizational Policy"] OR [mh "Fiscal Policy"] OR [mh Policy] OR [mh "Health Policy"] OR [mh Programs] OR [mh Sewage] OR [mh "Bathroom Equipment"] OR [mh Water] OR [mh Hygiene] OR [mh "Waste Management"] OR [mh "Nutrition Therapy"] OR [mh "Nutrition Policy"] OR [mh "Health Education"] OR [mh Counseling] OR [mh "Health Knowledge, Attitudes, Practice"] OR [mh immunization] OR [mh "Immunization Programs"] OR [mh "Community Health Aides"] OR [mh "Social Support"] OR [mh "Financing, Organized"] OR [mh "maternal welfare"] OR [mh "public policy"] OR [mh "social welfare"] OR [mh "social security"] OR [mh "Environmental Health"] OR [mh "Environmental Policy"] |
| #20 | ("health intervention" OR "public health" OR "health promotion" OR prevention OR protection OR "preventive health services" OR preventive OR control OR "child health" OR "child welfare" OR "child care" OR policy OR program OR project):ti,ab,kw OR (health NEAR/8 (intervention* OR prevention OR policy OR policies OR program* OR project*)):ti,ab,kw OR ("Communicable diseases" OR "infectious diseases" OR water OR sanitation OR hygiene OR "health knowledge" OR "Health attitude" OR practice OR immunization OR "Parasitic Diseases" OR "Psychosocial Support Systems" OR water OR sanitation OR latrine OR toilets OR "waste disposal" OR sewage OR hygiene OR "hand washing" OR "nutrition policy" OR "food policy" OR breastfeeding OR "nutrition therapy" OR supplement OR "parenting program" OR counseling OR "health education" OR instruction OR immunization OR immunization OR vaccine OR immune* OR inoculation OR insecticides OR "mosquito control" OR "bed nets" OR "mosquito nets" OR "vector control" OR "Prevention of mother-to-child transmission" OR PMTCT OR deworming OR "community health" OR outreach OR saving* OR voucher OR microfinance OR microcredit OR Welfare OR "public assistance" OR "social security" OR insurance OR "family policy" OR environment*):ti,ab,kw OR ((psychosocial NEAR/3 support) OR (social NEAR/3 support) OR (prevent* NEAR/3 transmission) OR ((finance* OR cash OR money) NEAR/3 transfer) OR (environment NEAR/3 (prevent* OR control*))):ti,ab,kw |
| #21 | #16 OR #17 OR #18 OR #19 |
| #22 | #20 OR #21 |
| #23 | #10 AND #15 AND #22 |
| #24 | [mh "Population Health"] OR [mh "Global Health"] OR [mh "Outcome Assessment (Health Care)"] |
| #25 | ("population health" OR "global health" OR "outcome assessment"):ti,ab,kw |
| #26 | #24 OR #25 |
| #27 | (effective* OR impact OR effect OR "outcome assessment" OR morbidity OR mortality OR death* OR disease* OR illness OR coverage OR (reported NEAR/3 cases) OR (cases NEAR/3 averted)):ti,ab,kw |
| #28 | #26 OR #27 |
| #29 | #23 AND #28 with Cochrane Library publication date Between Jan 2000 and Jan 2021 |

Search string in CAB Global Health

Ovid: Global Health 1973 to present

Initial search

**Number of hits** (as of 19/06/2019): **1,770**

**Search string**:

| Type of study | 1 | exp systematic reviews/ or exp meta-analysis/ |
| --- | --- | --- |
|  | 2 | literature reviews.sh. |
|  | 3 | (meta analy$ or metaanaly$ or (systematic adj (review$1 or overview$1))).ab,ti. |
|  | 4 | (cochrane or embase or (psychlit or psyclit) or (cinahl or cinhal) or (psychinfo or psycinfo) or science citation index or bids or cancerlit).ab. |
|  | 5 | (reference list$ or bibliograph$ or hand-search$ or relevant journals or manual search$).ab. |
|  | 6 | (selection criteria or data extraction).ab. |
|  | 7 | evidence synthes$.ab,ti. |
|  | 8 | ((systematic adj2 (review* or overview*)) or (umbrella adj2 review) or "review of reviews" or (systematic adj (review$1 or overview$1))).ab,ti. |
|  | 9 | 1 or 2 or 3 or 4 or 5 or 6 or 7 or 8 |
|  | 10 | (Comment or Letter or Editorial or animal or Published Erratum).hw. or conference paper.ab,ti. or (Surveys and Questionnaires).hw. or note.pt. |
|  | 11 | 9 not 10 |
| Population | 12 | (Africa or Asia or Caribbean or West Indies or South America or Middle East or Latin America or Central America).ab,hw,ti. |
|  | 13 | (developing countries or lmic or lmics or third world or lami countries or global south or transition*).ab,hw,ti. |
|  | 14 | ((developing or less developed or under developed or underdeveloped or middle income or low income or lower income or underserved or under served or deprived or poor) adj countr*).ab,hw,ti. |
|  | 15 | (low$ adj (gdp or gnp or gross domestic or gross national)).ab,hw,ti. |
|  | 16 | 12 or 13 or 14 or 15 |
|  | 17 | exp infants/ |
|  | 18 | exp preschool children/ or exp children/ |
|  | 19 | exp parents/ |
|  | 20 | 17 or 18 or 19 |
|  | 21 | (infant or newborn or neonate or baby or babies or toddler or pre-school or pediatric or child$ or perinatal or kindergarten or parent$).ab,hw,ti. |
|  | 22 | 20 or 21 |
|  | 23 | 16 and 22 |
| Intervention | 24 | exp child care/ |
|  | 25 | exp child welfare/ |
|  | 26 | exp child health/ |
|  | 27 | (health policy or public health or disease control or infectious diseases or infection control).sh. |
|  | 28 | exp health promotion/ |
|  | 29 | (disease prevention or prevention).sh. |
|  | 30 | preventive medicine.sh. |
|  | 31 | exp policy/ or exp nutrition policy/ or exp environmental policy/ or exp fiscal policy/ |
|  | 32 | exp programs/ |
|  | 33 | exp sewage/ |
|  | 34 | exp water/ |
|  | 35 | exp hygiene/ |
|  | 36 | exp waste management/ |
|  | 37 | exp nutritional support/ |
|  | 38 | exp health education/ |
|  | 39 | exp family counseling/ |
|  | 40 | exp immunization/ or exp immunization programmes/ |
|  | 41 | exp community health workers/ |
|  | 42 | finance.sh. |
|  | 43 | exp social welfare/ |
|  | 44 | exp social security/ |
|  | 45 | exp environmental health/ |
|  | 46 | 24 or 25 or 26 or 27 or 28 or 29 or 30 or 31 or 32 or 33 or 34 or 35 or 36 or 37 or 38 or 39 or 40 or 41 or 42 or 43 or 44 or 45 |
|  | 47 | (health intervention or public health or health promotion or prevention or protection or preventive health services or preventive or control or child health or child welfare or child care or policy or program or project).ab,hw,ti. |
|  | 48 | (health adj8 (intervention$ or prevention or policy or policies or program$ or project$)).ab,ti. |
|  | 49 | (Communicable disease$ or infectious disease$ or water or sanitation or hygiene or health knowledge or Health attitude or practice or immunization or Parasitic Diseases or Psychosocial Support Systems or water or sanitation or latrine or toilets or waste disposal or sewage or hygiene or hand wash$ or nutrition policy or food policy or breastfeeding or nutrition therapy or supplement or parenting program or counseling or health education or instruction or immuni#ation or vaccine or immune$ or inoculation or insecticides or mosquito control or bed nets or mosquito nets or vector control or Prevention of mother-to-child transmission or PMTCT or deworming or community health or outreach or saving$ or voucher or microfinance or microcredit or Welfare or public assistance or social security or insurance or family policy or environment$).ab,ti. |
|  | 50 | ((psychosocial adj3 support) or (social adj3 support) or (prevent$ adj3 transmission) or ((finance$ or cash or money) adj3 transfer) or (environment adj3 (prevent$ or control$))).ab,ti. |
|  | 51 | 47 or 48 or 49 or 50 |
|  | 52 | 46 or 51 |
| P+I+S | 53 | 11 and 23 and 52 |
| Outcomes | 54 | (population health or global health or outcome assessment).ab,hw,ti. |
|  | 55 | (effective* or impact or effect or outcome assessment or morbidity or mortality or death* or disease* or illness or coverage or (reported adj3 cases) or (cases adj3 averted)).ab,ti. |
|  | 56 | 54 or 55 |
| P+I+O+S | 57 | 53 and 56 |
| Year | 58 | limit 57 to yr="2000 -Current" |

Updated search

**Number of hits** (as of 19/01/2021): **622**

**Search string**:

| Type of study | 1 | exp systematic reviews/ or exp meta-analysis/ |
| --- | --- | --- |
|  | 2 | literature reviews.sh. |
|  | 3 | (meta analy$ or metaanaly$ or (systematic adj (review$1 or overview$1))).ab,ti. |
|  | 4 | (cochrane or embase or (psychlit or psyclit) or (cinahl or cinhal) or (psychinfo or psycinfo) or science citation index or bids or cancerlit).ab. |
|  | 5 | (reference list$ or bibliograph$ or hand-search$ or relevant journals or manual search$).ab. |
|  | 6 | (selection criteria or data extraction).ab. |
|  | 7 | evidence synthes$.ab,ti. |
|  | 8 | ((systematic adj2 (review* or overview*)) or (umbrella adj2 review) or "review of reviews" or (systematic adj (review$1 or overview$1))).ab,ti. |
|  | 9 | 1 or 2 or 3 or 4 or 5 or 6 or 7 or 8 |
|  | 10 | (Comment or Letter or Editorial or animal or Published Erratum).hw. or conference paper.ab,ti. or (Surveys and Questionnaires).hw. or note.pt. |
|  | 11 | 9 not 10 |
| Population | 12 | (Africa or Asia or Caribbean or West Indies or South America or Middle East or Latin America or Central America).ab,hw,ti. |
|  | 13 | (developing countries or lmic or lmics or third world or lami countries or global south or transition*).ab,hw,ti. |
|  | 14 | ((developing or less developed or under developed or underdeveloped or middle income or low income or lower income or underserved or under served or deprived or poor) adj countr*).ab,hw,ti. |
|  | 15 | (low$ adj (gdp or gnp or gross domestic or gross national)).ab,hw,ti. |
|  | 16 | 12 or 13 or 14 or 15 |
|  | 17 | exp infants/ |
|  | 18 | exp preschool children/ or exp children/ |
|  | 19 | exp parents/ |
|  | 20 | 17 or 18 or 19 |
|  | 21 | (infant or newborn or neonate or baby or babies or toddler or pre-school or pediatric or child$ or perinatal or kindergarten or parent$).ab,hw,ti. |
|  | 22 | 20 or 21 |
|  | 23 | 16 and 22 |
| Intervention | 24 | exp child care/ |
|  | 25 | exp child welfare/ |
|  | 26 | exp child health/ |
|  | 27 | (health policy or public health or disease control or infectious diseases or infection control).sh. |
|  | 28 | exp health promotion/ |
|  | 29 | (disease prevention or prevention).sh. |
|  | 30 | preventive medicine.sh. |
|  | 31 | exp policy/ or exp nutrition policy/ or exp environmental policy/ or exp fiscal policy/ |
|  | 32 | exp programs/ |
|  | 33 | exp sewage/ |
|  | 34 | exp water/ |
|  | 35 | exp hygiene/ |
|  | 36 | exp waste management/ |
|  | 37 | exp nutritional support/ |
|  | 38 | exp health education/ |
|  | 39 | exp family counseling/ |
|  | 40 | exp immunization/ or exp immunization programmes/ |
|  | 41 | exp community health workers/ |
|  | 42 | finance.sh. |
|  | 43 | exp social welfare/ |
|  | 44 | exp social security/ |
|  | 45 | exp environmental health/ |
|  | 46 | 24 or 25 or 26 or 27 or 28 or 29 or 30 or 31 or 32 or 33 or 34 or 35 or 36 or 37 or 38 or 42 or 43 or 44 or 45 |
|  | 47 | (health intervention or public health or health promotion or prevention or protection or preventive health services or preventive or control or child health or child welfare or child care or policy or program or project).ab,hw,ti. |
|  | 48 | (health adj8 (intervention$ or prevention or policy or policies or program$ or project$)).ab,ti. |
|  | 49 | (Communicable disease$ or infectious disease$ or water or sanitation or hygiene or health knowledge or Health attitude or practice or immunization or Parasitic Diseases or Psychosocial Support Systems or water or sanitation or latrine or toilets or waste disposal or sewage or hygiene or hand wash$ or nutrition policy or food policy or breastfeeding or nutrition therapy or supplement or parenting program or counseling or health education or instruction or immuni#ation or vaccine or immune$ or inoculation or insecticides or mosquito control or bed nets or mosquito nets or vector control or Prevention of mother-to-child transmission or PMTCT or deworming or community health or outreach or saving$ or voucher or microfinance or microcredit or Welfare or public assistance or social security or insurance or family policy or environment$).ab,ti. |
|  | 50 | ((psychosocial adj3 support) or (social adj3 support) or (prevent$ adj3 transmission) or ((finance$ or cash or money) adj3 transfer) or (environment adj3 (prevent$ or control$))).ab,ti. |
|  | 51 | 47 or 48 or 49 or 50 |
|  | 52 | 46 or 51 |
| P+I+S | 53 | 11 and 23 and 52 |
| Outcomes | 54 | (population health or global health or outcome assessment).ab,hw,ti. |
|  | 55 | (effective* or impact or effect or outcome assessment or morbidity or mortality or death* or disease* or illness or coverage or (reported adj3 cases) or (cases adj3 averted)).ab,ti. |
|  | 56 | 54 or 55 |
| P+I+O+S | 57 | 53 and 56 |
| Year | 58 | limit 57 to yr="2019-Current" |

Search string in Health Evidence

McMaster University: <https://www.healthevidence.org/search.aspx>

Initial search

**Number of hits** (as of 19/06/2019): **299**

**Search string**:

Results for: [(“health intervention” OR “public health” OR “health promotion” OR prevention OR protection OR “preventive health services” OR preventive OR control OR “child health” OR “child welfare” OR “child care” OR policy OR program OR project OR water OR sanitation OR hygiene OR “parenting program” OR counseling OR “health education” OR immunization OR vaccine OR Parasitic Diseases OR vector control OR outreach OR psychosocial OR financing OR social security OR insurance OR “family policy” OR environment*) AND (effective* OR impact OR effect OR "outcome assessment" OR morbidity OR mortality OR death* OR disease* OR illness OR coverage)] AND Limit:

Date = Published from 2000 to 2019

Review Type = Meta-analysis, Systematic review of reviews

Population = Infants (0-1 years), LMIC (low-to-middle-income countries), Preschool aged (1-4 years)

Topic Area = Communicable Disease/Infection, Environmental Health, Health Through the Ages -> Healthy Communities (e.g., community development, multicultural health, rural/urban health), Health Through the Ages -> Reproductive Health & Healthy Families, Nutrition, Social Determinants of Health (e.g., social environments, education, employment and working conditions)

Updated search (modified interface)

**Number of hits** (as of 11/01/2021): **116**

**Search string**:

Results for: [(“health intervention” OR “public health” OR “health promotion” OR prevention OR protection OR “preventive health services” OR preventive OR control OR “child health” OR “child welfare” OR “child care” OR policy OR program OR project OR water OR sanitation OR hygiene OR “parenting program” OR counseling OR “health education” OR immunization OR vaccine OR Parasitic Diseases OR vector control OR outreach OR psychosocial OR financing OR social security OR insurance OR “family policy” OR environment*) AND (effective* OR impact OR effect OR "outcome assessment" OR morbidity OR mortality OR death* OR disease* OR illness OR coverage)]

AND Limit:

Date = Published from 2019 to 2021

Review Type = Meta-analysis, Systematic review of reviews;

Population = Infants (0-1 years), LMIC (low-to-middle-income countries), Preschool aged (1-4 years)

Topic Area = Communicable Disease/Infection, Environmental Health, Health Through the Ages -> Healthy Communities (e.g., community development, multicultural health, rural/urban health), Health Through the Ages -> Reproductive Health & Healthy Families, Nutrition, Social Determinants of Health (e.g., social environments, education, employment and working conditions)

**Number of hits** (as of 11/01/2021): **320**

**Search string**:

Results for: [(“health intervention” OR “public health” OR “health promotion” OR prevention OR protection OR “preventive health services” OR preventive OR control OR “child health” OR “child welfare” OR “child care” OR policy OR program OR project OR water OR sanitation OR hygiene OR “parenting program” OR counseling OR “health education” OR immunization OR vaccine OR Parasitic Diseases OR vector control OR outreach OR psychosocial OR financing OR social security OR insurance OR “family policy” OR environment*) AND (effective* OR impact OR effect OR "outcome assessment" OR morbidity OR mortality OR death* OR disease* OR illness OR coverage)]

AND Limit:

Date = Published from 2000 to 2021

Review Type = narrative review

Population = Infants (0-1 years), LMIC (low-to-middle-income countries), Preschool aged (1-4 years)

Topic Area = Communicable Disease/Infection, Environmental Health, Health Through the Ages -> Healthy Communities (e.g., community development, multicultural health, rural/urban health), Health Through the Ages -> Reproductive Health & Healthy Families, Nutrition, Social Determinants of Health (e.g., social environments, education, employment and working conditions)

Search string in the Campbell Collaboration Library of Systematic Reviews

Initial search

The Campbell Library: <https://campbellcollaboration.org/library.html>

**Number of hits** (as of 21/06/2019): **33**

**Search string**:

Keyword: child AND health

Coordinating group(s): International Development; Knowledge Translation and Implementation; Nutrition; Social Welfare

Published date: 2000-01-01 to 2019-06-21

Type of documents: Protocol; Review

Updated search (modified interface)

**Number of hits** (as of 07/01/2021): 29

**Search string**:

[https://campbellcollaboration.org/component/jak2filter/?Itemid=1352&issearch=1&isc=1&category_id=101&searchword=child%20AND%20health&xf_3_from=2019-06-21&xf_3_to=2021-01-06&xf_4[0]=3&xf_4[1]=7&xf_4[2]=4&xf_4[3]=5&xf_8[0]=3&ordering=publishUp](https://campbellcollaboration.org/component/jak2filter/?Itemid=1352&issearch=1&isc=1&category_id=101&searchword=child%20AND%20health&xf_3_from=2019-06-21&xf_3_to=2021-01-06&xf_4%5b0%5d=3&xf_4%5b1%5d=7&xf_4%5b2%5d=4&xf_4%5b3%5d=5&xf_8%5b0%5d=3&ordering=publishUp)

Keyword: child AND health

Coordinating group(s): International Development; Knowledge Translation and Implementation; Nutrition; Social Welfare

Published date: 2000-01-01to 2021-01-07

Type of documents; Review

Search in 3ie Systematic review repository

International Initiative for Impact Evaluation - 3ie: https://www.3ieimpact.org/evidence-hub/systematic-review-repository

Initial search

Updated search

**Number of hits** (as of 24/06/2019): 110

**Search terms**:

child AND "public health" AND ("infectious diseases" OR "communicable diseases")

SR status: review; protocol

SR Type: Effectiveness review

Search in 3ie Evidence Hub (new interface)

Initial search

International Initiative for Impact Evaluation - 3ie: https://www.3ieimpact.org/evidence-hub/systematic-review-repository

**Number of hits** (as of 24/06/2019): 110

**Search terms**:

child AND "public health" AND ("infectious diseases" OR "communicable diseases")

SR status: review; protocol

SR Type: Effectiveness review

Updated search

International Initiative for Impact Evaluation - 3ie Evidence HUB: <https://developmentevidence.3ieimpact.org/>

**Number of hits** (as of 06/01/2019): 81

**Search terms**:

keywords:("Child Health") OR keywords:("Child") OR keywords:("Public Health") OR keywords:("Infectious Disease") OR keywords:("Communicable Diseases")

Filter: Systematic Reviews

Search string in Prospero

Centre for Reviews and Dissemination, University of York: <https://www.crd.york.ac.uk/prospero/>

Initial search

**Number of hits** (as of 19/06/2019): 23

**Search string**:

(child AND low- and middle-income countries):CT,KW,PA,RQ AND (Intervention OR Prevention OR Systematic Review OR Meta-Analysis OR Review of reviews):RT AND (child_health OR Public health including social determinants of health OR Tropical Medicine):HA WHERE CD FROM 01/01/2000 TO 19/06/2019

Updated search

**Number of hits** (as of 07/01/2021): 33

**Search string**:

(child AND low- and middle-income countries):CT,KW,PA,RQ AND (Intervention OR Prevention OR Systematic Review OR Meta-Analysis OR Review of reviews):RT AND (child_health OR Public health including social determinants of health OR Tropical Medicine):HA WHERE CD FROM 19/06/2019 TO 07/01/2021

**Manual search**

Search in Google Scholar

<https://scholar.google.com/>

Initial search

Advanced search for three separate search strings

**Number of hits** (as of 21/06/2019): 46

**Search terms**:

Find articles with all of the words: child health

with the exact phrase: systematic review

with at least one of the words: promotion prevention "infectious diseases" "public health" intervention "low * income" "middle * income" countries

where my words occur: In the title of the article

Dated between: 2000-2019

Find articles with all of the words: child health

with the exact phrase: meta-analysis

with at least one of the words: promotion prevention "infectious diseases" "public health" intervention "low * income" "middle * income" countries

where my words occur: In the title of the article

Dated between: 2000-2019

Find articles with all of the words: child health synthesis

with at least one of the words: promotion prevention "infectious diseases" "public health" intervention "low * income" "middle * income" countries

where my words occur: In the title of the article

Dated between: 2000-2019

Updated search

Advanced search for three separate search strings

**Number of hits** (as of 07/01/2021): **24**

**Search terms**:

Find articles with all of the words: child health

with the exact phrase: systematic review

with at least one of the words: promotion prevention "infectious diseases" "public health" intervention "low * income" "middle * income" countries

where my words occur: In the title of the article

Dated between: 2019-2021

Find articles with all of the words: child health

with the exact phrase: meta-analysis

with at least one of the words: promotion prevention "infectious diseases" "public health" intervention "low * income" "middle * income" countries

where my words occur: In the title of the article

Dated between: 2019-2021

Find articles with all of the words: child health synthesis

with at least one of the words: promotion prevention "infectious diseases" "public health" intervention "low * income" "middle * income" countries

where my words occur: In the title of the article

Dated between: 2019-2021

Search in UNICEF Office of Research – Innocenti

<https://www.unicef-irc.org/publications/>

Initial search

**Number of records selected** (as of 26/06/2019): 5 (after removing duplicates)

**Search terms**:

Search publication>advanced options, Keyword search: “health”

Three thesaurus searches for “Health”, “Cash transfer”, “Public policy”

Updated search

**Number of records selected** (as of 08/01/2021): 1

**Search terms**:

Search publication>advanced options, Keyword search: “health”

Three thesaurus searches for “Health”, “Cash transfer”, “Public policy”

Date: June 2019 – January 2021

Search in UNICEF websites:

Initial search

**Number of records selected** (as of 26/06/2019): 12 (after removing duplicates between the two websites)

https://www.unicef.org/publications/

Hand search of the resource pages under the Child survival theme.

<https://data.unicef.org/resources/>

**Search terms:**

Resource types: Journal article; Publications

Resource Topics: Diarrhoea; Early childhood development; health; HIV/AIDS; Immunization; Infant and young child feeding; Iodine; Malaria; Malnutrion; Mortality; Nutrition; Pneumonia; SDGs; Survival; Tuberculosis; Vitamin A; Water and sanitation

Updated search

**Number of records selected** (as of 08/01/2021): 2 (after removing duplicates between the two websites)

<https://www.unicef.org/reports> (new website for post-2019 publications)

Hand search of the resource pages under the Child survival theme.

<https://data.unicef.org/resources/>

**Search terms:**

Resource types: Journal article; Publications

Resource Topics: Diarrhoea; Early childhood development; health; HIV/AIDS; Immunization; Infant and young child feeding; Iodine; Malaria; Malnutrion; Mortality; Nutrition; Pneumonia; SDGs; Survival; Tuberculosis; Vitamin A; Water and sanitation

Resource Topics: 2019; 2020; 2021

Search in World Health Organization (WHO)

<http://www.who.int/>

Initial search

**Number of records selected** (as of 26/06/2019): 14 (after removing duplicates)

Updated search

**Number of records selected** (as of 08/01/2021): 0

IRIS repository

<https://apps.who.int/iris/>

Initial search

**Search terms**:

Date issues: 2000-2019

Title: Contains “review”

Title: Contains “child”

**Search terms**:

Date issues: 2000-2019

Title: Contains “review”

Subject: Contains “child”

**Search terms**:

Date issues: 2000-2019

Title: Contains “review”

Subject: Contains “public health”

Updated search

**Search terms**:

Date issues: 2019-2021

Title: Contains “review”

Title: Contains “child”

**Search terms**:

Date issues: 2019-2021

Title: Contains “review”

Subject: Contains “child”

**Search terms**:

Date issues: 2019-2021

Title: Contains “review”

Subject: Contains “public health”

WHO Child health publication page

Initial search

<https://www.who.int/maternal_child_adolescent/documents/year/en/>

Hand search for records published after 2000, with a title containing “review”

Updated search

<https://www.who.int/maternal_child_adolescent/documents/year/en/>

<https://www.who.int/publications/i?healthtopics=56e27fa8-d578-47b0-87c7-ed0bafa14b35&healthtopics-hidden=true> (new website)

Hand search for records published after 2019, with a title containing “review”
