# Supplementary material for: Which public health interventions are effective in reducing morbidity, mortality and health inequalities from infectious diseases amongst children in low- and middle-income countries (LMICs): An umbrella review
Source: PLoS One. 2021 Jun 10;16(6):e0251905. doi: 10.1371/journal.pone.0251905 (PMC8191901; doi:10.1371/journal.pone.0251905)
Supplement: S6 Appendix — (DOCX) [file pone.0251905.s006.docx]

# Annex 5: Extraction template

## Main extraction

| **Bibliographic details** | | | | | | |
| --- | --- | --- | --- | --- | --- | --- |
| Publication ID. | Author(s) | Publication Year | Title | Language | Link | Relevant Review Keywords |
| ####### | Free text | Number | Free text | Free text | Hyperlink | Keyword; Keyword;… |
| System Id in Rayyan |  |  |  |  |  | Topics in Rayyan (minus irrelevant ones) |

| **Review details** | | | | | | | | | | | | |
| --- | --- | --- | --- | --- | --- | --- | --- | --- | --- | --- | --- | --- |
| Type of review | | Countries covered in eligible studies | | Population(s) covered in eligible studies | | Years covered by the review search strategy | | Number of studies included in the review (total) | | Number of relevant studies included | | AMSTAR 2 rating |
| Page # | 1. Systematic review  2. Meta-analysis 3. Evidence synthesis | Page # | Country 1; Country 2;… | Page # | 1.Nenonate (<28 days) 2.Infant (<1 y.o) 3.Child (<5 y.o) 4.Household with children <5 | Page # | Free text | Page # | Number | Page # | Number | High Moderate Low Critically low |
|  |  |  | These are the countries covered in the synthesis of studies meeting the inclusion criteria, not the whole review |  | Target population(s) covered in the synthesis of studies meeting the inclusion criteria, not the whole review |  | Report limits or filters applied in the search regarding publication years |  | Number of studies included in the whole review |  | Number of studies included and synthesized in the review, which match our inclusion criteria | Please fill out the second tab of this document for each individual study |

| **Individual studies; synthesis characteristics** | | | | | | | | | | | | | | | | | |
| --- | --- | --- | --- | --- | --- | --- | --- | --- | --- | --- | --- | --- | --- | --- | --- | --- | --- |
| Included primary studies ref. | Study design(s) | | Type of control/comparison groups | | Setting and population | | Description of the intervention(s) | | Population Health outcome(s) reported | | | | Health Inequalities outcome(s) reported | | | |  |
| Page # | Page # | 1. Randomized controlled trials  2. Non-randomized controlled trials 3. Controlled observational studies  4. Before and after studies 5. Interrupted time-series studies 6. Natural policy experiments 7. Evaluation studies 8. Cohort studies 9. Case-control studies 10. Ecological studies | Page # | Free text | Page # | Free text | Page # | Free text | Page # | 1. Mortality 2. Morbidity 3. Number of Cases reported 4. Number of Cases averted 5. Number of deaths due to these diseases,  6. Diseases Incidence 7. Service/ intervention uptake 8. Service/ intervention coverage | Page # | Free text | Page # | Variation between groups or populations in 1. Mortality 2. Morbidity 3. Number of Cases reported 4. Number of Cases averted 5. Number of deaths due to these diseases,  6. Diseases Incidence 7. Service/ intervention uptake 8. Service/ intervention coverage | Page # | Free text |  |
| Please remember to report individual studies in the citation matrix |  | Type of study designs included in the synthesis (please select from the list). Please only report on the designs used in the studies included and synthesized in the review, which match our inclusion criteria. |  | Type of control/comparison groups used in the studies included in the synthesis. Please only report on the groups used in the studies included and synthesized in the review, which match our inclusion criteria. |  | Free text |  | Type/ Objective/ Process |  | Outcome measure used. Please keep one line per outcome. |  | Results |  | Outcome measure used. Please keep one line per outcome. |  | Results (variation in these health outcomes between groups or populations according to the Progress + factors) |  |

| **Intervention(s) and outcome summary** | | | | | | | | | | | |
| --- | --- | --- | --- | --- | --- | --- | --- | --- | --- | --- | --- |
| Type of intervention | | Summary of review | Level | Public health Function | Approach(es) to populations | Equity objective | PROGRESS+ factors covered | | Effect on population health | Effect on health inequalities |  |
| Page # | List (Table 1) | Free text | 1. Structural 2. Public policy 3. Social/ community 4. Individual/ household | 1. Promotion  2. Protection  3. Prevention | 1. Universal 2. Targeted  3. Proportionate universalism | 0. Not mentioned/not relevant 1. Health disadvantage  2. Health gap 3. Health gradient | 1. Place of Residence  2. Race, Ethnicity, cultural background  3. Occupation  4. Gender and sex  5. Religion 6. Education  7. Social Capital  8. Socio-economic status  9. +/Others | Free text | 0. Not reported/not relevant  1. Effective 2. Ineffective 3. unclear/insufficient evidence | 0. Not reported/not relevant 1. Positive 2. Neutral 3. Detrimental 4. unclear/insufficient evidence |  |
|  | Please refer to Table 1 in the protocol | A short paragraph summarizing: the aim; PICOS of the review/synthesis; its results. Please report potential heterogeneity/quality issues. | Please refer to the definitions in the protocol's framework | Please refer to the definitions in the protocol's framework | Please refer to the definitions in the protocol's framework | Please refer to the definitions in the protocol's framework | Please refer to Table 2 in the protocol | Details on the factor(s) covered (see examples in Table 2) | Please refer to the protocol's framework | Please refer to the protocol's framework |  |

## AMSTAR 2 Checklist

When assessing individual studies, please refer to the AMSTAR 2 checklist and guidance document. (Shea et al., 2017) Then, please report your answer in the table below.

| Publication ID. | [Publication ID.] | [Publication ID.] |
| --- | --- | --- |
| Overall rating  *High: 0 or 1 non-critical weakness (critical items are marked with a ‘*’)*  *Moderate: >1 non-critical weakness*  *Low: 1 critical weakness with or without non-critical weaknesses*  *Critically low: >1 critical weakness with or without non-critical weaknesses* | High  Moderate  Low  Critically low | High  Moderate  Low  Critically low |
| 1. Did the research questions and inclusion criteria for the review include the components of PICO? | Yes  No | Yes  No |
| *2. Did the report of the review contain an explicit statement that the review methods were established prior to the conduct of the review and did the report justify any significant deviations from the protocol? | Yes  Partial yes  No | Yes  Partial yes  No |
| 3. Did the review authors explain their selection of the study designs for inclusion in the review? | Yes  No | Yes  No |
| *4. Did the review authors use a comprehensive literature search strategy? | Yes  Partial yes  No | Yes  Partial yes  No |
| 5. Did the review authors perform study selection in duplicate? | Yes  No | Yes  No |
| 6. Did the review authors perform data extraction in duplicate? | Yes  No | Yes  No |
| *7. Did the review authors provide a list of excluded studies and justify the exclusions? | Yes  Partial yes  No | Yes  Partial yes  No |
| 8. Did the review authors describe the included studies in adequate detail? | Yes  Partial yes  No | Yes  Partial yes  No |
| *9. Did the review authors use a satisfactory technique for assessing the risk of bias (RoB) in individual studies that were included in the review? | Yes  Partial yes  No | Yes  Partial yes  No |
| 10. Did the review authors report on the sources of funding for the studies included in the review? | Yes  No | Yes  No |
| *11. If meta-analysis was performed did the review authors use appropriate methods for statistical combination of results? | Yes  No  NA | Yes  No  NA |
| 12. If meta-analysis was performed, did the review authors assess the potential impact of RoB in individual studies on the results of the meta-analysis or other evidence synthesis? | Yes  No  NA | Yes  No  NA |
| *13. Did the review authors account for RoB in individual studies when interpreting/ discussing the results of the review? | Yes  No | Yes  No |
| 14. Did the review authors provide a satisfactory explanation for, and discussion of, any heterogeneity observed in the results of the review? | Yes  No | Yes  No |
| *15. If they performed quantitative synthesis did the review authors carry out an adequate investigation of publication bias (small study bias) and discuss its likely impact on the results of the review? | Yes  No  NA | Yes  No  NA |
| 16. Did the review authors report any potential sources of conflict of interest, including any funding they received for conducting the review? | Yes  No | Yes  No |

## Citation matrix

| No. | Reference | Multiple  articles  reported  as one  study | *SR 1* | *SR2* | *…* |
| --- | --- | --- | --- | --- | --- |
|  |  |  |  |  |  |
|  | Number of references | |  |  |  |
|  | Number of studies | |  |  |  |
|  |  |  |  |  |  |
| 1 | *Primary 1* |  |  |  |  |
| 2 | *Primary2* |  |  |  |  |
| 3 | … |  |  |  |  |
| 4 |  |  |  |  |  |
| 5 |  |  |  |  |  |
| 6 |  |  |  |  |  |
| 7 |  |  |  |  |  |
| 8 |  |  |  |  |  |
| 9 |  |  |  |  |  |
| 10 |  |  |  |  |  |
| 11 |  |  |  |  |  |
| 12 |  |  |  |  |  |
| 13 |  |  |  |  |  |
| … |  |  |  |  |  |
