# Supplementary material for: Which public health interventions are effective in reducing morbidity, mortality and health inequalities from infectious diseases amongst children in low- and middle-income countries (LMICs): An umbrella review
Source: PLoS One. 2021 Jun 10;16(6):e0251905. doi: 10.1371/journal.pone.0251905 (PMC8191901; doi:10.1371/journal.pone.0251905)
Supplement: S7 Appendix — (DOCX) [file pone.0251905.s007.docx]

# S7 Appendix: List of excluded studies

# The publication’s full text cannot be obtained by the review team

*Reviews withdrawn*

1. Nisar, M. I., Jehan, F., Shafiq, Y., Lassi, Z. S., & Zaidi, A. K. (2016). Conjugate vaccines for preventing Haemophilus influenzae type b infections in children under 5 years of age. Cochrane Database of Systematic Reviews, 2016(10). https://www.cochrane.org/CD010377/ARI_conjugate-vaccines-preventing-haemophilus-influenzae-type-b-infections-children-under-5-years-age
2. Jehan, F., Nisar, M. I., Lassi, Z. S., Omer, S. B., & Zaidi, A. K. M. (2017). Oral polio vaccine plus inactivated polio vaccine versus oral polio vaccine alone for reducing polio in children under two years of age. Cochrane Database of Systematic Reviews, 2017(1). https://www.cochranelibrary.com/cdsr/doi/10.1002/14651858.CD010857.pub2/full

*Protocols without published reviews*

1. Brown JVE, Walsh V, McGuire W. Birth room transition support for preterm infants: A cochrane overview. Cochrane Database Syst Rev. 2019;2019(9):1–7.
2. Chachou, M. J., Mukinda, F. K., Motaze, V., & Wiysonge, C. S. (2015). Electronic and postal reminders for improving immunisation coverage in children: Protocol for a systematic review and meta-analysis. BMJ Open, 5(10), e008310.
3. Demessie HF, Hailemariam DH, Kloos HK, Adugna AA, Mekonnen WM. Protocol for Systematic Review on Inequity in Child Health Care Service Utilization in Low and Middle-income Countries. 2020;
4. Desai S, Mehta K, Jyoti Singh R, Dirusu O, Wong C, Westley A, et al. What is the effect of integrated economic and health interventions with women’s groups on health-related knowledge, behaviours and outcomes in low-and middle-income countries? PROSPERO [Internet]. 2020; Available from: <https://www.crd.york.ac.uk/prospero/display_record.php?RecordID=199998>
5. Dickinson-Craig E, Woolley K, Bartington S, Oludotun T, Pope F, Singh A, et al. Effectiveness of interventions, to reduce household air pollution from solid biomass fuels to improve pregnancy and child health outcomes in Low and Middle Income Countries: a systematic review protocol. PROSPERO [Internet]. 2020; Available from: https://www.crd.york.ac.uk/prospero/display_record.php?RecordID=164998
6. Evans, K., King, R., Elsey, H., Das, M., Putnis, N., Barua, D., Rassi, C., Cartwright, C., & Ferdous, T. (2017). Community engagement interventions for communicable disease control and management in low and lower middle income countries: An umbrella review. PROSPERO. http://www.crd.york.ac.uk/PROSPERO/display_record.php?ID=CRD42017074134
7. Fewtrell M, Nicholls A, McCann L, Fair F, Kalea A, Soltani H. Effects of interventions to increase exclusive breastfeeding on health outcomes in infants and children up to 7 years of age: protocol for a systematic review and meta-analysis. PROSPERO [Internet]. 2020; Available from: https://www.crd.york.ac.uk/prospero/display_record.php?RecordID=203796
8. Gurung R, Zaka N, Budhathoki SS, Sunny AK, Thapa J, Zhou H, et al. Study protocol: Impact of quality improvement interventions on perinatal outcomes in health facilities-a systematic review. Systematic reviews. 2019;8(1):205.
9. James, N., & Lawson, K. (2019). A systematic review of Result Based Financing (RBF) in maternal and child health for low- and middle-income countries (LMICs); what do we know, do not know and need to know ? PROSPERO. http://www.crd.york.ac.uk/PROSPERO/display_record.php?ID=CRD42019133119
10. Kagina, B. M., Wiysonge, C. S., Machingaidze, S., Abdullahi, L. H., Adebayo, E., Uthman, O. A., & Hussey, G. D. (2014). The use of supplementary immunisation activities to improve uptake of current and future vaccines in low-income and middle-income countries: A systematic review protocol. BMJ Open, 4(2), e004429.
11. Kalata NL, Nyazika TK, Swarthout TD, Everett D, French N, Heyderman RS, et al. Pneumococcal pneumonia and carriage in Africa before and after introduction of pneumococcal conjugate vaccines, 2000-2019: protocol for systematic review. BMJ open. 2019;9(11):e030981.
12. Lattof SR, Maliqi B. Private sector delivery of quality care for maternal, newborn and child health in low-income and middle-income countries: a mixed-methods systematic review protocol. BMJ open. 2020;10(2):e033141.
13. Likka, M. H., Handalo, D. M., Weldsilase, Y. A., & Sinkie, S. O. (2018). The effect of community-based health insurance schemes on utilization of healthcare services in low- and middle-income countries: A systematic review protocol of quantitative evidence. JBI Database Of Systematic Reviews And Implementation Reports, 16(3), 653–661.
14. Little, M., Roelen, K., Yakubovich, A., Steinert, J., Cluver, L., & Humphreys, D. K. (2018). Implementation and evaluation of packaged cash-plus interventions to accelerate progress toward achieving the SDGs for infants and children in low- and middle-income countries: A systematic review and meta-analysis. PROSPERO. http://www.crd.york.ac.uk/PROSPERO/display_record.php?ID=CRD42018108017
15. Lufumpa, N. (2018). The role of infrastructure in the improvement of child health in low and middle income countries: A systematic review of the impact of different forms of infrastructure on the health of children in low and middle income countries. PROSPERO. http://www.crd.york.ac.uk/PROSPERO/display_record.php?ID=CRD42018096922
16. Meshak, D., & Greenwood, B. (2018). The effect of mass drug administration of azithromycin for the management of trachoma on overall child mortality: A systematic review. PROSPERO. http://www.crd.york.ac.uk/PROSPERO/display_record.php?ID=CRD42018104162
17. Molloy, C., Beatson, R., Goldfeld, S., Harrop, C., & Perini, N. (2018). Sustained nurse home visiting programs for disadvantaged families with young children: Protocol for a restricted systematic review of program effectiveness and components associated with enhanced health, well-being, and life-course outcomes. PROSPERO. http://www.crd.york.ac.uk/PROSPERO/display_record.php?ID=CRD42018106781
18. Momberg, D., Ngandu, C., May, J., Norris, S., & Said-Mohamed, R. (2017). Governance of water, sanitation and hygiene (WASH) in sub-Saharan Africa and associations with nutritional status in children under five years of age: A systematic review. Annals of Nutrition and Metabolism, 71, 886–887.
19. Patel, S. (2018). Structural, institutional and organizational factors associated with successful pay for performance programmes in improving quality of maternal and child health care in low and middle income countries: A systematic literature review. Journal of Global Health, 8(2), 021001.
20. Piper, J. D., Chandna, J., Allen, E., Linkman, K., Cumming, O., Prendergast, A. J., & Gladstone, M. J. (2017). Water, sanitation and hygiene (WASH) interventions: Effects on child development in low- and middle-income countries. Cochrane Database of Systematic Reviews, 2017(3). https://www.cochranelibrary.com/cdsr/doi/10.1002/14651858.CD012613/full
21. Sguassero, Y., Booker, D., Dennis, J. A., Orellano, A., & Abalos, E. (2017). Supplementary feeding with nutritional education for caregivers for promoting growth and development in young children in developing countries. Cochrane Database of Systematic Reviews, 2017. http://as.wiley.com/WileyCDA/Brand/id-6.html
22. Siddiqui, F. J., Gaffey, M., Bhutta, Z. A., Ataullahjan, A., Als, D., Kamali, M., Munyuzangabo, M., Jain, R., Meteke, S., & Shah, S. (2019). Delivery strategies for reproductive, maternal, neonatal, child and adolescent health and nutrition (RMNCAH&N) intervention in conflict settings in low- and middle-income countries. PROSPERO. http://www.crd.york.ac.uk/PROSPERO/display_record.php?ID=CRD42019125221
23. Vollmer, S., Khan, S., Tu, L. T. N., Pasha, A., & Sahoo, S. (2017). Protocol: The effect of interventions for women’s empowerment on children’s health and education: A systematic review of evidence from low- and middle-income countries. Campbell Collaboration. <https://campbellcollaboration.org/library/women-economic-empowerment-effect-on-children.html>
24. Waddington H. Water, sanitation and hygiene (WASH) for reducing mortality in childhood in low- and middle-income countries. PROSPERO [Internet]. 2020; Available from: https://www.crd.york.ac.uk/prospero/display_record.php?RecordID=210694

# The publication is a primary study, a conference proceeding or paper, an abstract, editorial, letter, comment, erratum, survey, note or a doctoral thesis; or does not meet one or more of the three key elements of systematic reviews and evidence syntheses as defined in PICOS; or does not synthesize at least 2 relevant primary studies.

1. Abou-Nader, A. J., Sauer, M. A., Steele, A. D., Tate, J. E., Atherly, D., Parashar, U. D., Santosham, M., & Nelson, E. A. S. (2018). Global rotavirus vaccine introductions and coverage: 2006—2016. Human Vaccines & Immunotherapeutics, 14(9), 2281–2296.
2. Adetokunboh, O. O., & Oluwasanu, M. (2016). Eliminating mother-to-child transmission of the human immunodeficiency virus in sub-Saharan Africa: The journey so far and what remains to be done. Journal of Infection and Public Health, 9(4), 396–407.
3. Ahmed, M., & Won, Y. (2017). Cross-national systematic review of neonatal mortality and postnatal newborn care: Special focus on Pakistan. International Journal of Environmental Research and Public Health, 14(12). <https://www.ncbi.nlm.nih.gov/pmc/articles/PMC5750861/>
4. Alavi, S. M., & Alavi, L. (2016). Toxoplasmosis in Iran: A guide for general physicians working in the Iranian health network setting: A systematic review. Caspian Journal of Internal Medicine, 7(4), 233–241.
5. Amaral, M., Guedes, G., Epifanio, M., Wagner, M., Jones, M., & Mattiello, R. (2017). Network meta-analysis of probiotics to prevent respiratory infections in children and adolescents. Pediatric Pulmonology, 52(6), 833–843.
6. Assefa, Y., Gelaw, Y. A., Hill, P. S., Taye, B. W., & Damme, W. van. (2019). Community health extension program of Ethiopia, 2003-2018: Successes and challenges toward universal coverage for primary healthcare services. Globalization and Health, 15(24).
7. Avila-Aguero, M. L., Beltran, S., Castillo, J. B. D., Castillo Diaz, M. E., Chaparro, L. E., Deseda, C., Debbag, R., Espinal, C., Falleiros-Arlant, L. H., Gonzalez Mata, A. J., Macias Parra, M., Marques-Rosa, F., Catalina Pirez, M., & Vazquez-Rivera, M. (2018). Varicella epidemiology in Latin America and the Caribbean. Expert Review of Vaccines, 17(2), 175–183.
8. Balster, R. L., Levy, S., & Stammer, E. (2014). Evidence acquisition and evaluation for evidence summit on population-level behavior change to enhance child survival and development in low- and middle-income countries. Journal of Health Communication: International Perspectives; 2014, 1. https://www.tandfonline.com/doi/full/10.1080/10810730.2014.918215
9. Baumgartner, J., & Barth-Jaeggi, T. (2015). Iron interventions in children from low-income and middle-income populations: Benefits and risks. Current Opinion in Clinical Nutrition and Metabolic Care, 18(3), 289–294.
10. Berglund, S., & Domellöf, M. (2014). Meeting iron needs for infants and children. Current Opinion in Clinical Nutrition and Metabolic Care, 17(3), 267–272.
11. Bibera, G. L., Chen, J., Pereira, P., & Benninghoff, B. (2020). Dynamics of G2P[4] strain evolution and rotavirus vaccination: A review of evidence for Rotarix. Vaccine, 38(35), 5591–5600.
12. Budhathoki, S. S., Meika, B., Yadav, A. K., Pawan, U., & Pokharel, P. K. (2016). Eco-social and behavioural determinants of diarrhoea in under-five children of Nepal: A framework analysis of the existing literature. Tropical Medicine and Health, 44(7). https://tropmedhealth.biomedcentral.com/track/pdf/10.1186/s41182-016-0006-9
13. Buralli, R. J., Dultra, A. F., & Ribeiro, H. (2020). Respiratory and Allergic Effects in Children Exposed to Pesticides-A Systematic Review. International Journal of Environmental Research and Public Health, 17(8), 16.
14. Burnett, E. P. U. D. T. J. E. (2020). Real-world effectiveness of rotavirus vaccines, 2006-19: A literature review and meta-analysis. The Lancet. Global Health, 8(9), e1195–e1202.
15. Burnett, E., Parashar, U. D., & Tate, J. E. (2020). Global impact of rotavirus vaccination on diarrhea hospitalizations and deaths among children <5 years old: 2006-2019. The Journal of Infectious Diseases, 10.1093/infdis/jiaa081.
16. Chamla, D., Luo, C., Adjorlolo-Johnson, G., Vandelaer, J., Young, M., Costales, M. O., & McClure, C. (2015). Integration of HIV infant testing into immunization programmes: A systematic review. Paediatrics and International Child Health, 35(4), 298–304.
17. Citron, I., Chokotho, L., & Lavy, C. (2016). Prioritisation of Surgery in the National Health Strategic Plans of Africa: A Systematic Review. World Journal of Surgery, 40(4), 779–783.
18. Clark, A., van Zandvoort, K., Flasche, S., Sanderson, C., Bines, J., Tate, J., Parashar, U., & Jit, M. (2019). Efficacy of live oral rotavirus vaccines by duration of follow-up: A meta-regression of randomised controlled trials. The Lancet: Infectious Diseases, 19(7), 717–727.
19. Cleminson, J., & McGuire, W. (2015). Topical emollient for prevention of infection in preterm infants: A systematic review. Lancet, 385, S31.
20. Cohen, R., Cohen, J. F., Chalumeau, M., & Levy, C. (2017). Impact of pneumococcal conjugate vaccines for children in high- and non–high-income countries. Expert Review of Vaccines, 16(6), 625–640.
21. Conklin, L., Loo, J. D., Kirk, J., Fleming-Dutra, K. E., Deloria Knoll, M., Park, D. E., & et al. (2014). Systematic review of the effect of pneumococcal conjugate vaccine dosing schedules on vaccine-type invasive pneumococcal disease among young children. Pediatric Infectious Disease Journal, 33(2), 109–118.
22. Cooper, J. E., Benmarhnia, T., Koski, A., & King, N. B. (2020). Cash transfer programs have differential effects on health: A review of the literature from low and middle-income countries. Social Science and Medicine, 247. https://www.scopus.com/inward/record.uri?eid=2-s2.0-85079405056&doi=10.1016%2fj.socscimed.2020.112806&partnerID=40&md5=4d16be09639125a0ab6cbc665b188773
23. Costello, A., & Dalglish, S. L. (2016). Towards a grand convergence for child survival and health: A strategic review of options for the future building on lessons learnt from IMNCI. World Health Organization. https://www.who.int/maternal_child_adolescent/documents/strategic-review-child-health-imnci/en/
24. Crepaz, N., Tungol-Ashmon, M. V., Vosburgh, H. W., Baack, B. N., & Mullins, M. M. (2015). Are couple-based interventions more effective than interventions delivered to individuals in promoting HIV protective behaviors? A meta-analysis. AIDS Care, 27(11), 1361–1366.
25. Cruz, R. C. de S., Moura, L. B. A. de, & Soares Neto, J. J. (2017). Conditional cash transfers and the creation of equal opportunities of health for children in low and middle-income countries: A literature review. International Journal for Equity in Health, 16(1), 161. https://doi.org/10.1186/s12939-017-0647-2
26. Das, J. K., Hadi, Y. B., Salam, R. A., Hoda, M., Lassi, Z. S., & Bhutta, Z. A. (2018). Fly control to prevent diarrhoea in children. Cochrane Database of Systematic Reviews, 12. http://dx.doi.org/10.1002/14651858.CD011654.pub2
27. De-Regil, L. M., Jefferds, M. E. D., & Peña-Rosas, J. P. (2017). Point-of-use fortification of foods with micronutrient powders containing iron in children of preschool and school-age. Cochrane Database of Systematic Reviews, 2017(11). https://www.scopus.com/inward/record.uri?eid=2-s2.0-85034859686&doi=10.1002%2f14651858.CD009666.pub2&partnerID=40&md5=b57ccf7849640ed17e30e4db9be7dc25
28. Dol, J., Campbell-Yeo, M., Tomblin Murphy, G., Aston, M., McMillan, D., Gahagan, J., & Richardson, B. (2019). Parent-targeted postnatal educational interventions in low and middle-income countries: A scoping review and critical analysis. International Journal of Nursing Studies, 94, 60–73.
29. Dossa, N. I., Philibert, A., & Dumont, A. (2016). Using routine health data and intermittent community surveys to assess the impact of maternal and neonatal health interventions in low-income countries: A systematic review. (Special Issue: Maternal and neonatal health in Africa at the MDG end: Availability of and access to maternal health services and outcomes of intervention strategies.). International Journal of Gynecology & Obstetrics, 135, S64–S71.
30. Dunlap, J., Foderingham, N., Bussell, S., Wester, C. W., Audet, C. M., & Aliyu, M. H. (2014). Male involvement for the prevention of mother-to-child HIV transmission: A brief review of initiatives in East, West, and Central Africa. Current HIV/AIDS Reports, 11(2), 109–118.
31. Elder, J. P., Pequegnat, W., Ahmed, S., Bachman, G., Bullock, M., Carlo, W. A., Chandra-Mouli, V., Fox, N. A., Harkness, S., Huebner, G., Lombardi, J., Murry, V. M., Moran, A., Norton, M., Mulik, J., Parks, W., Raikes, H. H., Smyser, J., Sugg, C., … Ulkuer, N. (2014). Caregiver behavior change for child survival and development in low- and middle-income countries: An examination of the evidence. Journal of Health Communication, 19, 25–66.
32. Ellis, C. M., & Chaffin, J. (2015). Evaluations of outcomes for children and youth from NGO-supported microeconomic interventions: A research synthesis. Enterprise Development and Microfinance, 26(2), 104–121.
33. Emre, I. E., Eroglu, Y., Kara, A., Dinleyici, E. C., & Ozen, M. (2019). The effect of probiotics on prevention of upper respiratory tract infections in the paediatric community—A systematic review. Beneficial Microbes, 11(3), 201–211.
34. Fiorati, R. C., Arcencio, R. A., Segura del Pozo, J., Ramasco-Gutierrez, M., & Serrano-Gallardo, P. (2018). Intersectorality and social participation as coping policies for health inequities-worldwide. Gaceta Sanitaria, 32(3), 304–314.
35. Frew, P., & Lutz, C. (2017). Interventions to increase pediatric vaccine uptake: An overview of recent findings. Human Vaccines & Immunotherapeutics, 13(11), 2503–2511.
36. Frost, L., & Pratt, B. A. (2014). Review of the literature on factors contributing to the reductions of maternal and child mortality in low income and middle income countries: An evidence synthesis for the success factors study [Partnership for Maternal, Newborn & Child Health Technical Paper]. Geneva: World Health Organization; 2014. Global Health Insights.
37. Garnett, G. P., Krishnaratne, S., Rush, S. H., Hallett, T. B., & Hargreaves, J. R. (2016). The cost-effectiveness, affordability and impact of HIV Prevention: Concepts and Reviews. AIDS Research and Human Retroviruses, 32, 299.
38. Goh, A. E. N., Choi, E. H., Chokephaibulkit, K., Choudhury, J., Kuter, B., Lee, P.-I., Marshall, H., Kim, J. O., & Wolfson, L. J. (2019). Burden of varicella in the Asia-Pacific region: A systematic literature review. Expert Review of Vaccines, 18(5), 475–493.
39. Gumede-Moyo, S., Filteau, S., Munthali, T., Todd, J., & Musonda, P. (2017). Implementation effectiveness of revised (post-2010) World Health Organization guidelines on prevention of mother-to-child transmission of HIV using routinely collected data in sub-Saharan Africa: A systematic literature review. Medicine, 96(40), e8055.
40. Halioua, B., & Lobel, B. (2014). Actual controversies about circumcision. [French]. Presse Medicale, 43(11), 1168–1173.
41. Hernandez-Avila, M., Lazcano-Ponce, E., Hernandez-Avila, J. E., Alpuche-Aranda, C. M., Rodriguez-Lopez, M. H., Garcia-Garcia, L., Madrid-Marina, V., Lopez Gatell-Ramirez, H., Lanz-Mendoza, H., Martinez-Barnetche, J., Diaz-Ortega, J. L., Angeles-Llerenas, A., Barrientos-Gutierrez, T., Bautista-Arredondo, S., & Santos-Preciado, J. I. (2016). [Analysis of the evidence on the efficacy and safety of CYD-TDV dengue vaccine and its potential licensing and implementation through Mexico’s Universal Vaccination Program]. Salud Publica de Mexico, 58(1), 71–83.
42. House, S. A., & Ralston, S. L. (2017). Diagnosis, prevention, and management of bronchiolitis in children: Review of current controversies. Minerva Pediatrica, 69(2), 141–155.
43. Hungerford, D., Smith, K., Tucker, A., Iturriza-Gomara, M., Vivancos, R., McLeonard, C., N, A. C., & French, N. (2017). Population effectiveness of the pentavalent and monovalent rotavirus vaccines: A systematic review and meta-analysis of observational studies. BMC Infectious Diseases, 17(1), 569.
44. Hunter, B. M., & Murray, S. F. (2017). Demand-side financing for maternal and newborn health: What do we know about factors that affect implementation of cash transfers and voucher programmes? BMC Pregnancy & Childbirth, 17(1), 262.
45. Hutton, G., & Chase, C. (2017). Water Supply, Sanitation, and Hygiene. The International Bank for Reconstruction and Development / The World Bank. 3rd Chapter, 9(10), 27.
46. John, D., & Issac, A. (2018). Do mass deworming efforts improve the developmental health and well-being of children in low-and middle-income countries? Summary of the evidence and implications for public health programmes. Clinical Epidemiology and Global Health, 6(4), 220–224.
47. Karim, T., Muhit, M., & Khandaker, G. (2017). Interventions to prevent respiratory diseases—Nutrition and the developing world. Paediatric Respiratory Reviews, 22, 31–37.
48. Kim, S. S., Patel, M., & Hinman, A. (2017). Use of m-Health in polio eradication and other immunization activities in developing countries. Vaccine, 35(10), 1373–1379.
49. Kraft, J. M., Wilkins, K. G., Morales, G. J., Widyono, M., & Middlestadt, S. E. (2014). An evidence review of gender-integrated interventions in reproductive and maternal-child health. Journal of Health Communication, 19, 122–141.
50. Kuruvilla, S., Schweitzer, J., Bishai, D., Chowdhury, S., Caramani, D., Frost, L., Cortez, R., Daelmans, B., Francisco, A. de, Adam, T., Cohen, R., Alfonso, Y. N., Franz-Vasdeki, J., Saadat, S., Pratt, B. A., Eugster, B., Bandali, S., Venkatachalam, P., Hinton, R., … Bustreo, F. (2014). Success factors for reducing maternal and child mortality. Bulletin of the World Health Organization, 92, 533–544. https://doi.org/10.2471/BLT.14.138131
51. Lassi, Z. S., Kedzior, S. G. E., & Bhutta, Z. A. (2020). Maternal-newborn educational care packages for neonatal health and survival: A systematic review and meta-analysis of evidence from low and middle-income countries. Journal of Paediatrics and Child Health, 56, 96.
52. Lassi, Z. S., Kurji, J., Oliveira, C. S. D., Moin, A., & Bhutta, Z. A. (2020). Zinc supplementation for the promotion of growth and prevention of infections in infants less than six months of age. Cochrane Database of Systematic Reviews, 4. http://dx.doi.org/10.1002/14651858.CD010205.pub2
53. Liberato, S. C., Singh, G., & Mulholland, K. (2015). Zinc supplementation in young children: A review of the literature focusing on diarrhoea prevention and treatment. Clinical Nutrition, 34(2), 181–188.
54. Lindsey, B. B., Armitage, E. P., Kampmann, B., & de Silva, T. I. (2019). The efficacy, effectiveness, and immunogenicity of influenza vaccines in Africa: A systematic review. The Lancet. Infectious Diseases, 19(4), e110–e119.
55. Linguissi, L. S. G., Ouattara, A. K., Ntambwe, E. K., Mbalawa, C. G., & Nkenfou, C. N. (2018). Mobile applications: Effective tools against HIV in Africa. Health and Technology, 8(4), 215–222.
56. Loevinsohn, M., Mehta, L., Cuming, K., Nicol, A., Cumming, O., & Ensink, J. H. (2015). The cost of a knowledge silo: A systematic re-review of water, sanitation and hygiene interventions. Health Policy & Planning, 30(5), 660–674.
57. Loo, J. D., Conklin, L., Fleming-Dutra, K. E., Deloria Knoll, M., Park, D. E., Kirk, J., Goldblatt, D., O’Brien, K. L., & Whitney, C. G. (2014). Systematic review of the effect of pneumococcal conjugate vaccine dosing schedules on prevention of pneumonia. Pediatric Infectious Disease Journal, 33, S140-51.
58. Loo, J. D., Conklin, L., Fleming-Dutra, K. E., Deloria Knoll, M., Park, D. E., Kirk, J., Goldblatt, D., O’Brien, K. L., & Whitney, C. G. (2014). Systematic review of the effect of pneumococcal conjugate vaccine dosing schedules on prevention of pneumonia. Pediatric Infectious Disease Journal, 33, S140-51.
59. Maguina, C., & Galan-Rodas, E. (2016). The Zika virus: A literature review. Acta Medica Peruana, 33(1), 35–41.
60. Mangtani, P., Abubakar, I., Ariti, C., Beynon, R., Pimpin, L., Fine, P. E. M., Rodrigues, L. C., Smith, P. G., Lipman, M., Whiting, P. F., & Sterne, J. A. (2014). Protection by BCG vaccine against tuberculosis: A systematic review of randomized controlled trials. Clinical Infectious Diseases, 58(4), 470–480.
61. Martinez-Quintana, E., Castillo-Solorzano, C., Torner, N., & Rodriguez-Gonzalez, F. (2015). Congenital rubella syndrome: A matter of concern. Pan American Journal of Public Health, 37(3), 179–186.
62. Masekela, R., & Vanker, A. (2020). Lung health in children in sub-Saharan Africa: Addressing the need for cleaner air. International Journal of Environmental Research and Public Health, 17(17). https://www.mdpi.com/1660-4601/17/17/6178
63. Mazige, F. M., Kalwani, J. D., & Kakoko, D. C. V. (2016). Social determinants of immunization services uptake in developing countries: A systematic review. Pan African Medical Journal, 24. https://www.scopus.com/inward/record.uri?eid=2-s2.0-85007504333&doi=10.11604%2fpamj.2016.24.197.9605&partnerID=40&md5=e787138942cd6de1b809dde3db09b43f
64. Meteke, S., Stefopulos, M., Als, D., Gaffey, M., Kamali, M., Siddiqui, F. J., Munyuzangabo, M., Jain, R. P., Shah, S., Radhakrishnan, A., Ataullahjan, A., & Bhutta, Z. A. (2020). Delivering infectious disease interventions to women and children in conflict settings: A systematic reviefw. BMJ Global Health, 5. http://ovidsp.ovid.com/ovidweb.cgi?T=JS&PAGE=reference&D=prem&NEWS=N&AN=32341087
65. Moreira, M., Cintra, O., Harriague, J., Hausdorff, W. P., & Hoet, B. (2016). Impact of the introduction of the pneumococcal conjugate vaccine in the Brazilian routine childhood national immunization program. Vaccine, 34(25), 2766–2778.
66. Moya-Alvarez, V., Bodeau-Livinec, F., & Cot, M. (2016). Iron and malaria: A dangerous liaison? Nutrition Reviews, 74(10), 612–623.
67. Munn, Z., Tufanaru, C., Lockwood, C., Stern, C., McAneney, H., & Barker, T. H. (2020). Rinse-free hand wash for reducing absenteeism among preschool and school children. The Cochrane Database of Systematic Reviews, 2020(2), CD012566.
68. Musa, T. H., Wei, P., & Pu, Y. (2015). Review: Health literacy intervention and their consequences. Journal of Public Health and Epidemiology, 7(3), 71–75.
69. Nabavi, S. F., Sureda, A., Daglia, M., Izadi, M., Rastrelli, L., & Nabavi, S. M. (2017). Flavonoids and chagas’ disease: The story so far! Current Topics in Medicinal Chemistry, 17(7). https://www.scopus.com/inward/record.uri?eid=2-s2.0-85011958977&doi=10.2174%2f1568026616666160824110141&partnerID=40&md5=fcb4eb8a21334d7e3b8574271eef7d2c
70. Najafi, F., Sayehmiri, K., & Najafi, R. (2018). Efficacy of hepatitis B vaccination in under five-year-old children in Iran: A systematic review and meta-analysis study. Hepatitis Monthly, 18(6). http://hepatmon.com/en/articles/65385.html
71. Ndiaye, S. (2017). The equity fund in Senegal: An analysis of the mechanisms of health insurance for the poor and their prospects for universal health coverage. (Special Issue: Health governance in Africa: Taking stock.) [French]. Africa Development / Afrique et Developpement, 42(1), 9–31.
72. Nour, R. (2019). A Systematic Review of Methods to Improve Attitudes Towards Childhood Vaccinations. Cureus, 11(7), e5067.
73. Oldenburg, C. E., Arzika, A. M., Amza, A., Gebre, T., Kalua, K., Mrango, Z., Cotter, S. Y., West, S. K., Bailey, R. L., Emerson, P. M., O’Brien, K. S., Porco, T. C., Keenan, J. D., & Lietman, T. M. (2019). Mass Azithromycin Distribution to Prevent Childhood Mortality: A Pooled Analysis of Cluster-Randomized Trials. American Journal of Tropical Medicine & Hygiene, 100(3), 691–695.
74. Omoniyi, O. S., & Williams, I. (2020). Realist Synthesis of the International Theory and Evidence on Strategies to Improve Childhood Vaccination in Low- and Middle-Income Countries: Developing Strategies for the Nigerian Healthcare System. International Journal of Health Policy and Management, 9(7), 274–285.
75. Ordonez, J. E., & Orozco, J. J. (2015). Cost-effectiveness analysis of the available pneumococcal conjugated vaccines for children under five years in Colombia. Cost Effectiveness and Resource Allocation, 13(6). http://www.resource-allocation.com/content/13/1/6
76. Parashar, U. D., Nelson, E. A. S., & Gagandeep, K. (2014). Diagnosis, management, and prevention of rotavirus gastroenteritis in children. Bmj, 347. http://www.bmj.com/content/347/bmj.f7204
77. Perry, H. B., Rassekh, B. M., Sundeep, G., Wilhelm, J., & Freeman, P. A. (2017). Comprehensive review of the evidence regarding the effectiveness of community-based primary health care in improving maternal, neonatal and child health: 1. Rationale, methods and database description. (Research Theme: Evidence for community-based PHC in improving MNCH.). Journal of Global Health, 7(1). http://www.jogh.org/documents/issue201701/jogh-07-010901.pdf
78. Pickering, A. J., Null, C., Winch, P. J., Mangwadu, G., Arnold, B. F., Prendergast, A. J., Njenga, S. M., Rahman, M., Ntozini, R., Benjamin-Chung, J., Stewart, C. P., Huda, T. M. N., Moulton, L. H., Colford, J. M., Jr., Luby, S. P., & Humphrey, J. H. (2019). The WASH Benefits and SHINE trials: Interpretation of WASH intervention effects on linear growth and diarrhoea. Lancet Global Health, 7(8), e1139–e1146.
79. Price, A., Verma, A., & Welfare, W. (2015). Are health education interventions effective for the control and prevention of urogenital schistosomiasis in sub-Saharan Africa? A systematic review. Transactions of the Royal Society of Tropical Medicine & Hygiene, 109(4), 239–244.
80. Roelen, K., Devereux, S., Abdulai, A.-G., Martorano, B., Palermo, T., & Ragno, L. P. (2017). How to Make ‘Cash Plus’ Work: Linking Cash Transfers to Services and Sectors (Innocenti Working Papers 2017/10; p. 42). UNICEF Office of Research - Innocenti. https://doi.org/10.18356/0e6268b5-en
81. Rosas-Peralta, M., Holick, M. F., Borrayo-Sanchez, G., Madrid-Miller, A., Ramirez-Arias, E., & Arizmendi-Uribe, E. (2017). Dysfunctional immunometabolic effects of vitamin D deficiency, increased cardiometabolic risk. Potential epidemiological alert in America? Endocrinologia Diabetes y Nutricion, 64(3), 162–173.
82. Rudan, I., Patel, S., Waters, D., Wazny, K., Campbell, I., Sridhar, D., Chopra, M., Campbell, H., Pfaffmann-Zambruni, J., Chandan, U., & Young, M. (2016). Integrated Management of Childhood Illness (IMCI) in the 21st Century: A Review of the Scientific and Programmatic Evidence. UNICEF. https://www.unicef.org/health/files/UNICEF_Review_of_Evidence_Working_Paper_1_FINAL_11Oct2016.pdf
83. Seguin, M., & Nino Zarazua, M. (2015). Non-clinical interventions for acute respiratory infections and diarrhoeal diseases among young children in developing countries. Tropical Medicine & International Health, 20(2), 146–169.
84. Shankar, R., & Hunter, A. (2019). Community-based innovations in maternal health aimed to reduce maternal and neonatal mortality in south east Asia. Journal of Investigative Medicine, 67, 76.
85. Silva, S. M., Rodrigues, I. C. G., Santos, R. da S., & Ternes, Y. M. F. (2020). The direct and indirect effects of the pneumococcal conjugated vaccine on carriage rates in children aged younger than 5 years in Latin America and the Caribbean: A systematic review. Einstein (Sao Paulo, Brazil), 18, eRW4890.
86. Sondaal, S. F., Browne, J. L., Amoakoh-Coleman, M., Borgstein, A., Miltenburg, A. S., Verwijs, M., & Klipstein-Grobusch, K. (2016). Assessing the Effect of mHealth Interventions in Improving Maternal and Neonatal Care in Low- and Middle-Income Countries: A Systematic Review. PLoS ONE [Electronic Resource], 11(5), e0154664.
87. Soriano-Arandes, A., Angheben, A., Serre-Delcor, N., Trevino-Maruri, B., Gomez, I. P. J., & Jackson, Y. (2016). Control and management of congenital Chagas disease in Europe and other non-endemic countries: Current policies and practices. Tropical Medicine & International Health, 21(5), 590–596.
88. Spearman, C. W., Afihene, M., Ally, R., Apica, B., Awuku, Y., Cunha, L., Dusheiko, G., Gogela, N., Kassianides, C., Kew, M., Lam, P., Lesi, O., Lohouès-Kouacou, M. J., Mbaye, P. S., Musabeyezu, E., Musau, B., Ojo, O., Rwegasha, J., Scholz, B., … Sonderup, M. W. (2017). Hepatitis B in sub-Saharan Africa: Strategies to achieve the 2030 elimination targets. The Lancet Gastroenterology and Hepatology, 2(12), 900.
89. Stuurman, A., Marano, C., Bunge, E., De Moerlooze, L., & Shouval, D. (2017). Impact of universal mass vaccination with monovalent inactivated hepatitis A vaccines- A systematic review. Human Vaccines and Immunotherapeutics, 13(3), 724–736.
90. Su, S. B., Chang, H. L., & Chen, K. T. (2020). Current Status of Mumps Virus Infection: Epidemiology, Pathogenesis, and Vaccine. International Journal of Environmental Research and Public Health, 17(5), 15.
91. Thwaites, C. L., & Loan, H. T. (2015). Eradication of tetanus. British Medical Bulletin, 116, 69–77.
92. Tin Tin Htar, M., Jackson, S., Balmer, P., Serra, L. C., Vyse, A., Slack, M., Riera-Montes, M., Swerdlow, D. L., & Findlow, J. (2020). Systematic literature review of the impact and effectiveness of monovalent meningococcal C conjugated vaccines when used in routine immunization programs. BMC Public Health, 20(1), 1890.
93. Tirivayi, N., Richardson, D., Gavrilovic, M., Groppo, V., Kajula, L., Valli, E., & Viola, F. (2020). A Rapid Review of Economic Policy and Social Protection Responses to Health and Economic Crises and Their Effects on Children. Lessons for the COVID-19 pandemic response. Innocenti Working Papers, 2020. https://www.unicef-irc.org/publications/1095-rapid-review-economic-policy-social-protection-responses-to-health-and-economic-crises.html
94. Toska, E., Gittings, L., Hodes, R., Cluver, L. D., Govender, K., Chademana, K. E., & Gutierrez, V. E. (2016). Resourcing resilience: Social protection for HIV prevention amongst children and adolescents in Eastern and Southern Africa. African Journal of AIDS Research, 15(2), 123–140.
95. Vaivada, T., Gaffey, M. F., Das, J. K., & Bhutta, Z. A. (2017). Evidence-based interventions for improvement of maternal and child nutrition in low-income settings: What’s new? Current Opinion in Clinical Nutrition & Metabolic Care, 20(3), 204–210.
96. Vélez, L. F., Sanitato, M., Barry, D., Alilio, M., Apfel, F., Coe, G., Garcia, A., Kaufman, M., Klein, J., Kutlesic, V., Meadowcroft, L., Nilsen, W., O’Sullivan, G., Peterson, S., Raiten, D., & Vorkoper, S. (2014). The Role of Health Systems and Policy in Producing Behavior and Social Change to Enhance Child Survival and Development in Low- and Middle-Income Countries: An Examination of the Evidence. Journal of Health Communication, 19, 89–121.
97. Victora, C. G., Bahl, R., Barros, A. J., Franca, G. V., Horton, S., Krasevec, J., Murch, S., Sankar, M. J., Walker, N., Rollins, N. C., & Lancet Breastfeeding Series, G. (2016). Breastfeeding in the 21st century: Epidemiology, mechanisms, and lifelong effect. Lancet, 387(10017), 475–490.
98. Vrazo, A. C., Firth, J., Amzel, A., Sedillo, R., Ryan, J., & Phelps, B. R. (2018). Interventions to significantly improve service uptake and retention of HIV-positive pregnant women and HIV-exposed infants along the prevention of mother-to-child transmission continuum of care: Systematic review. Tropical Medicine & International Health, 23(2), 136–148.
99. Wahl, B., Lehtimaki, S., Germann, S., & Schwalbe, N. (2020). Expanding the use of community health workers in urban settings: A potential strategy for progress towards universal health coverage. Health Policy and Planning, 35(1), 91–101.
100. Walque, D., Fernald, L., Gertler, P., & Hidrobo, M. (2017). Cash Transfers and Child and Adolescent Development. The International Bank for Reconstruction and Development / The World Bank. 3rd Chapter, 23(11), 20.
101. Wang, C. M., Chen, S. C., & Chen, K. T. (2015). Current status of rotavirus vaccines. World Journal of Pediatrics, 11(4), 300–308.
102. Webb, C., & Cabada, M. M. (2018). A Review on Prevention Interventions to Decrease Diarrheal Diseases’ Burden in Children. Current Tropical Medicine Reports, 5(1), 31–40.
103. Wisniewski, J., Acosta, A., Kolaczinski, J., Koenker, H., & Yukich, J. (2019). Systematic review and meta-analysis of the cost and cost-effectiveness of distributing insecticide-treated nets for the prevention of malaria. Acta Tropica, 202, 105229.
104. Wood, R., & Bekker, L. G. (2014). Isoniazid preventive therapy for tuberculosis in South Africa: An assessment of the local evidence base. South African Medical Journal. Suid-Afrikaanse Tydskrif Vir Geneeskunde, 104(3), 174–177.
105. World Health Organization. (2020). Assessments of sexual, reproductive, maternal, newborn, child and adolescent health in the context of universal health coverage in six countries in the WHO European Region: A synthesis of findings from the country reports (rayyan-131217305).
106. Wrottesley, S. V., Lamper, C., & Pisa, P. T. (2016). Review of the importance of nutrition during the first 1000 days: Maternal nutritional status and its associations with fetal growth and birth, neonatal and infant outcomes among African women. (Special Issue: Developmental origins of health and disease: Importance of research for Africa.). Journal of Developmental Origins of Health and Disease, 7(2), 144–162.
107. Yang Gg, K. D. P. A. P. C. J. (2018). A meta-regression analysis of the effectiveness of mosquito nets for malaria control: The value of long-lasting insecticide nets. International Journal of Environmental Research and Public Health, 15(3), 546.

# The publication is a review of reviews

1. Ashrita, S., White, H., Albright, K., & Adona, J. (2020). Mega-map of systematic reviews and evidence and gap maps on the interventions to improve child well-being in low- and middle-income countries. Campbell Systematic Reviews, 16(4). https://onlinelibrary.wiley.com/doi/10.1002/cl2.1116
2. Coster, S., Watkins, M., & Norman, I. J. (2018). What is the impact of professional nursing on patients’ outcomes globally? An overview of research evidence. International Journal of Nursing Studies, 78, 76–83.
3. Das, J. K., Kumar, R., Salam, R. A., Lassi, Z. S., & Bhutta, Z. A. (2014). Evidence from facility level inputs to improve quality of care for maternal and newborn health: Interventions and findings. Reproductive Health, 11(2), S4.
4. English, R., Peer, N., Honikman, S., Tugendhaft, A., & Hofman, K. J. (2017). “First 1000 days” health interventions in low- and middle-income countries: Alignment of South African policies with high-quality evidence. Glob Health Action, 10(1), 1340396.
5. Ezeh, A., Oyebode, O., Satterthwaite, D., Chen, Y. F., Ndugwa, R., Sartori, J., Mberu, B., Melendez-Torres, G. J., Haregu, T., Watson, S. I., Caiaffa, W., Capon, A., & Lilford, R. J. (2017). The history, geography, and sociology of slums and the health problems of people who live in slums. The Lancet, 389(10068), 547–558.
6. Kappagoda, S., & Ioannidis, J. P. (2014). Prevention and control of neglected tropical diseases: Overview of randomized trials, systematic reviews and meta-analyses. Bulletin of the World Health Organization, 92(5), 356–366.
7. Lassi, Z. S., Das, J. K., Salam, R. A., & Bhutta, Z. A. (2014). Evidence from community level inputs to improve quality of care for maternal and newborn health: Interventions and findings. Reproductive Health, 11(2), S2.
8. Lassi, Z. S., Mallick, D., Das, J. K., Mal, L., Salam, R. A., & Bhutta, Z. A. (2014). Essential interventions for child health. Reproductive Health, 11(Suppl 1), S4. https://doi.org/10.1186/1742-4755-11-S1-S4
9. Lassi, Z., Middleton, P., Crowther, C., & Bhutta, Z. (2015). Interventions to improve neonatal health and later survival: An overview of systematic reviews. EBioMedicine, 2(8), 985–1000.
10. Lazzerini, M. (2014). Micronutrients for the Prevention and Treatment of Diarrhea in Children in Low- and Middle-Income Countries. Current Tropical Medicine Reports, 1(2), 106–110.
11. Magwood, O., Kpadé, V., Thavorn, K., Oliver, S., Mayhew, A. D., & Pottie, K. (2019). Effectiveness of home-based records on maternal, newborn and child health outcomes: A systematic review and meta-analysis. PLoS ONE, 14(1).
12. Martineau, A. R., Jolliffee, D. A., Hooper, R. L., Greenberg, L., Aloia, J. F., Berman, P., & al, et. (2017). Vitamin D supplementation to prevent acute respiratory tract infections: Systematic review and meta-analysis of individual participant data. British Medical Journal, 356, doi: 10.1136/bmj.i6583.
13. Pantoja, T., Opiyo, N., Lewin, S., Paulsen, E., Ciapponi, A., Wiysonge, C. S., Herrera, C. A., Rada, G., Peñaloza, B., Dudley, L., Gagnon, M. P., Marti, S. G., & Oxman, A. D. (2017). Implementation strategies for health systems in low-income countries: An overview of systematic reviews. Cochrane Database of Systematic Reviews, 2017(9).
14. Questa, K., Das, M., King, R., Everitt, M., Rassi, C., Cartwright, C., Ferdous, T., Barua, D., Putnis, N., Snell, A. C., Huque, R., Newell, J., & Elsey, H. (2020). Community engagement interventions for communicable disease control in low- and lower- middle-income countries: Evidence from a review of systematic reviews. International Journal for Equity in Health, 19(1), 51.
15. Salam, R. A., Lassi, Z. S., Das, J. K., & Bhutta, Z. A. (2014). Evidence from district level inputs to improve quality of care for maternal and newborn health: Interventions and findings. (Special Issue: Quality of care in maternal and child health.). Reproductive Health, 11. http://www.reproductive-health-journal.com/content/pdf/1742-4755-11-S2-S3.pdf
16. Salam, R. A., Mansoor, T., Mallick, D., Lassi, Z. S., Das, J. K., & Bhutta, Z. A. (2014). Essential childbirth and postnatal interventions for improved maternal and neonatal health. Reproductive Health, 11, S3.
17. Thomson, K., Hillier-Brown, F., Walton, N., Bilaj, M., Bambra, C., & Todd, A. (2019). The effects of community pharmacy-delivered public health interventions on population health and health inequalities: A review of reviews. Preventive Medicine, 124, 98–109. https://doi.org/10.1016/j.ypmed.2019.04.003
18. Visser, J., McLachlan, M. H., Maayan, N., & Garner, P. (2018). Community-based supplementary feeding for food insecure, vulnerable and malnourished populations—An overview of systematic reviews. Cochrane Database of Systematic Reviews, 11, CD010578.

# The review has been updated

1. Anwar, E., Goldberg, E., Fraser, A., Acosta, C. J., Paul, M., & Leibovici, L. (2014). Vaccines for preventing typhoid fever. Cochrane Database of Systematic Reviews, 1, CD001261.
2. Nurmatov, U. B., Lee, S. H., Nwaru, B. I., Mukherjee, M., Grant, L., & Pagliari, C. (2014). The effectiveness of mHealth interventions for maternal, newborn and child health in low- and middle-income countries: Protocol for a systematic review and meta-analysis. Journal of Global Health, 4(1), 010407.
3. Puchalski Ritchie, L. M., van Lettow, M., Pham, B., Straus, S. E., Hosseinipour, M. C., Rosenberg, N. E., Phiri, S., Landes, M., Cataldo, F., & the, P. consortium. (2019). What interventions are effective in improving uptake and retention of HIV-positive pregnant and breastfeeding women and their infants in prevention of mother to child transmission care programmes in low-income and middle-income countries? A systematic review and meta-analysis. BMJ Open, 9(7), e024907.
4. Puchalski Ritchie, L. M., van Lettow, M., Pham, B., Straus, S. E., Hosseinipour, M. C., Rosenberg, N. E., Phiri, S., Landes, M., Cataldo, F., & the, P. consortium. (2019). Correction: What interventions are effective in improving uptake and retention of HIV-positive pregnant and breastfeeding women and their infants in prevention of mother to child transmission care programmes in low-income and middle-income countries? A systematic review and meta-analysis (BMJ Open (2019) 9 (e024907) DOI: 10.1136/bmjopen-2018-024907). BMJ Open, 9(8), e024907corr1.

# The review captured by the updated search was already included

1. Morita, T., Godfrey, S., & George, C. M. (2016). Systematic review of evidence on the effectiveness of safe child faeces disposal interventions. Tropical Medicine & International Health, 21(11), 1403–1419.
2. Mureed, S., Somronghtong, R., Kumar, R., Ghaffar, A., & Chapman, R. S. (2015). Enhanced Immunization Coverage through Interventions for Childhood Cluster Diseases in Developing Countries. Journal of Ayub Medical College, Abbottabad: JAMC, 27(1), 223–227.
3. Ngocho, J. S., Magoma, B., Olomi, G. A., Mahande, M. J., Msuya, S. E., de Jonge, M. I., & Mmbaga, B. T. (2019). Effectiveness of pneumococcal conjugate vaccines against invasive pneumococcal disease among children under five years of age in Africa: A systematic review. PLoS ONE [Electronic Resource], 14(2), e0212295.
4. Yuan, B., Målqvist, M., Trygg, N., Qian, X., Ng, N., & Thomsen, S. (2014). What interventions are effective on reducing inequalities in maternal and child health in low- and middle-income settings? A systematic review. BMC Public Health, 14(1), 634. https://doi.org/10.1186/1471-2458-14-634

# The publication only includes interventions in country/ies the World Bank historical classification has continuously defined as high-income between 2000 and 2019, (The World Bank, 2019) or does not synthesize or report on low-, lower-middle or upper-middle-income countries separately.

1. Adamu, A. A., Uthman, O. A., Wambiya, E. O., Gadanya, M. A., & Wiysonge, C. S. (2019). Application of quality improvement approaches in health-care settings to reduce missed opportunities for childhood vaccination: A scoping review. Human Vaccines and Immunotherapeutics
2. Berman-Rosa, M., O’Donnell, S., Barker, M., & Quach, C. (2020). Efficacy and effectiveness of the PCV-10 and PCV-13 vaccines against invasive pneumococcal disease. Pediatrics, 145(4), 1–14.
3. Bryant-Lukosius, D., Carter, N., Reid, K., Donald, F., Martin-Misener, R., Kilpatrick, K., Harbman, P., Kaasalainen, S., Marshall, D., Charbonneau-Smith, R., & DiCenso, A. (2015). The clinical effectiveness and cost-effectiveness of clinical nurse specialist-led hospital to home transitional care: A systematic review. Journal of Evaluation in Clinical Practice, 21(5), 763–781.
4. Burns, J., Boogaard, H., Polus, S., Pfadenhauer, L. M., Rohwer, A. C., Erp, A. M. van, Turley, R., & Rehfuess, E. (2019). Interventions to reduce ambient particulate matter air pollution and their effect on health. Cochrane Database of Systematic Reviews, 5, CD010919.
5. Chen, T. Y., Hendrickx, A., Stevenson, D. S., Bird, P., & Walls, T. (2020). No evidence from a systematic review for the use of probiotics to prevent otitis media. Acta Paediatrica (Oslo, Norway : 1992), 10.1111/apa.15368.
6. Ciapponi, A., Bardach, A., Rey Ares, L., Glujovsky, D., Cafferata, M. L., Cesaroni, S., & Bhatti, A. (2019). Sequential inactivated (IPV) and live oral (OPV) poliovirus vaccines for preventing poliomyelitis. The Cochrane Database of Systematic Reviews, 12, CD011260.
7. Cruchet, S., Furnes, R., Maruy, A., Hebel, E., Palacios, J., Medina, F., Ramirez, N., Orsi, M., Rondon, L., & Sdepanian, V. (2015). The use of probiotics in pediatric gastroenterology: A review of the literature and recommendations by Latin-American experts. Pediatric Drugs, 17(3), 199–216.
8. de Cock, C., van Velthoven, M., Milne-Ives, M., Mooney, M., & Meinert, E. (2020). Use of apps to promote childhood vaccination: Systematic review. JMIR: Journal of Medical Internet Research MHealth and UHealth, 8(5), e17371.
9. De Oliveira, L. H., Jauregui, B., Carvalho, A. F., & Giglio, N. (2017). Impact and effectiveness of meningococcal vaccines: A review. Revista Panamericana De Salud Publica-Pan American Journal of Public Health, 41, 19.
10. Ejemot-Nwadiaro, R. I., Ehiri, J. E., Arikpo, D., Meremikwu, M. M., & Critchley, J. A. (2020). Hand‐washing promotion for preventing diarrhoea. Cochrane Database of Systematic Reviews, 12. http://dx.doi.org/10.1002/14651858.CD004265.pub4
11. Fedele, D., Cushing, C., Fritz, A., Amaro, C., & Ortega, A. (2017). Mobile health interventions for improving health outcomes in youth: A meta-analysis. JAMA Pediatrics, 171(5), 461–469.
12. Jacobson Vann, J. C., Jacobson, R. M., Coyne‐Beasley, T., Asafu‐Adjei, J. K., & Szilagyi, P. G. (2018). Patient reminder and recall interventions to improve immunization rates. Cochrane Database of Systematic Reviews, 1. https://doi.org//10.1002/14651858.CD003941.pub3
13. Jefferson, T., Jones, M. A., Doshi, P., Thompson, M. J., Spencer, E. A., Onakpoya, I. J., & et al. (2014). Neuraminidase inhibitors for preventing and treating influenza in healthy adults and children. Cochrane Database of Systematic Reviews, 2014(4), Art. No.: CD008965.
14. Jefferson, T., Rivetti, A., Pietrantonj, C. di, & Demicheli, V. (2018). Vaccines for preventing influenza in healthy children. Cochrane Database of Systematic Reviews, 2. https://www.cochranelibrary.com/cdsr/doi/10.1002/14651858.CD004879.pub5/full
15. Kapoor, V. S., Evans, J. R., & Vedula, S. S. (2020). Interventions for preventing ophthalmia neonatorum. The Cochrane Database of Systematic Reviews, 9, CD001862.
16. Karafillakis, E., Hassounah, S., & Atchison, C. (2015). Effectiveness and impact of rotavirus vaccines in Europe, 2006-2014. Vaccine, 33(18), 2097–2107.
17. King, S., Tancredi, D., Lenoir-Wijnkoop, I., Gould, K., Vann, H., Connors, G., & et al. (2019). Does probiotic consumption reduce antibiotic utilization for common acute infections? A systematic review and meta-analysis. European Journal of Public Health, 29(3), 494–499.
18. Laursen, R. P., & Hojsak, I. (2018). Probiotics for respiratory tract infections in children attending day care centers- A systematic review. European Journal of Pediatrics, 177(7), 979–994.
19. Lohner, S., Kullenberg, D., Antes, G., Decsi, T., & Meerpohl, J. J. (2014). Prebiotics in healthy infants and children for prevention of acute infectious diseases: A systematic review and meta-analysis. Nutrition Reviews, 72(8), 523–531.
20. Macartney, K., Heywood, A., & McIntyre, P. (2014). Vaccines for post-exposure prophylaxis against varicella (chickenpox) in children and adults. Cochrane Database of Systematic Reviews, 2014(6), CD001833.
21. MacIntyre, C. R., & Chughtai, A. A. (2020). A rapid systematic review of the efficacy of face masks and respirators against coronaviruses and other respiratory transmissible viruses for the community, healthcare workers and sick patients. International Journal of Nursing Studies, 108, 103629.
22. Malagon-Rojas, J. N., Mantziari, A., Salminen, S., & Szajewska, H. (2020). Postbiotics for preventing and treating common infectious diseases in children: A systematic review. Nutrients, 12(2). https://www.ncbi.nlm.nih.gov/pmc/articles/PMC7071176/
23. Mayo-Wilson, E., Junior, J. A., Imdad, A., Dean, S., Chan, X. H., Chan, E. S., Jaswal, A., & Bhutta, Z. A. (2014). Zinc supplementation for preventing mortality, morbidity, and growth failure in children aged 6 months to 12 years of age. Cochrane Database of Systematic Reviews, 5, CD009384.
24. Molloy, C., Beatson, R., Harrop, C., Perini, N., & Goldfeld, S. (2021). Systematic review: Effects of sustained nurse home visiting programs for disadvantaged mothers and children. Journal of Advanced Nursing, 77(1), 147–161.
25. Padhani, Z. A., Moazzam, Z., Ashraf, A., Bilal, H., Salam, R. A., Das, J. K., & Bhutta, Z. A. (2020). Vitamin C supplementation for prevention and treatment of pneumonia. The Cochrane Database of Systematic Reviews, 4, CD013134.
26. Palmer, M. J., Henschke, N., Bergman, H., Villanueva, G., Maayan, N., Tamrat, T., Mehl, G. L., Glenton, C., Lewin, S., Fonhus, M. S., & Free, C. (2020). Targeted client communication via mobile devices for improving maternal, neonatal, and child health. The Cochrane Database of Systematic Reviews, 8, CD013679.
27. Prutsky, G. J., Domecq, J. P., Elraiyah, T., Prokop, L. J., & Murad, M. H. (2014). Assessing the evidence: Live attenuated influenza vaccine in children younger than 2 years. A systematic review. Pediatric Infectious Disease Journal, 33(4), E106–E115.
28. Rana, R., McGrath, M., Gupta, P., Thakur, E., & Kerac, M. (2020). Feeding Interventions for Infants with Growth Failure in the First Six Months of Life: A Systematic Review. Nutrients, 12(7).
29. Richardson, B. P., van der Linde, J., Pillay, B., & Swanepoel, D. W. (2020). Do text messages about health and development in young children affect caregiver behaviour and child outcomes? A systematic review. Health Education Journal.
30. Rivero-Santana, A., Cuellar-Pompa, L., Sanchez-Gomez, L. M., Perestelo-Perez, L., & Serrano-Aguilar, P. (2014). Effectiveness and cost-effectiveness of different immunization strategies against whooping cough to reduce child morbidity and mortality. Health Policy, 115(1), 82–91.
31. Rosnah, S., Hoon, C., Suliana Mohamad, S., Siti Nor, M., Lu, Y., Massitah, M., Norazilah, J., Hidayatul Fariha, S., Sharul Rizan, I., Siti Hasmah, I., Mohd Normazlan, H., Syafiq, T., Gunaseelan, G., Humadevi, S., Muhammad Naim, M. S., Hamenudin, H., Norzaher, I., & Ida Dalina, N. (2017). A systematic review on health interventions used in enhancing vaccination uptake. Clinics in Mother and Child Health, 14(2). https://www.omicsonline.org/open-access/a-systematic-review-on-health-interventions-used-in-enhancing-vaccinationuptake-2090-7214-1000264.php?aid=90669
32. Rostami, S. S., Moradi-Lakeh, M., Esteghamati, A., Mobinizadeh, M., Shokraneh, F., Babashahi, S., & Yaghoubi, M. (2014). Efficacy and safety of rotavirus vaccine in children under five year; systematic review and meta-analysis. Journal of Isfahan Medical School, 32(303), 1605–1622.
33. Skorka, A., Piescik-Lech, M., Kolodziej, M., & Szajewska, H. (2017). To add or not to add probiotics to infant formulae? An updated systematic review. Beneficial Microbes, 8(5), 717–725.
34. Soares‐Weiser, K., Bergman, H., Henschke, N., Pitan, F., & Cunliffe, N. (2019). Vaccines for preventing rotavirus diarrhoea: Vaccines in use. Cochrane Database of Systematic Reviews, 3. http://dx.doi.org/10.1002/14651858.CD008521.pub4
35. Stocks, M. E., Ogden, S., Haddad, D., Addiss, D. G., McGuire, C., & Freeman, M. C. (2014). Effect of water, sanitation, and hygiene on the prevention of trachoma: A systematic review and meta-analysis. PLoS Medicine / Public Library of Science, 11(2), 1–29.
36. Tol, W., Greene, M. C., Lasater, M. E., Le Roch, K., Bizouerne, C., Purgato, M., Tomlinson, M., & Barbui, C. (2018). Impact of Maternal Mental Health Interventions on Child-Related Outcomes in Low-and Middle-Income Countries: A Systematic Review and Meta-Analysis. Available at SSRN 3208958.
37. Vorilhon, P., Arpajou, B., Vaillant Roussel, H., Merlin, E., Pereira, B., & Cabaillot, A. (2019). Efficacy of vitamin C for the prevention and treatment of upper respiratory tract infection: A meta-analysis in children. European Journal of Clinical Pharmacology, 75(3), 303–311.
38. Wang, Y., Li, X., Ge, T., Xiao, Y., Liao, Y., Cui, Y., Zhang, Y., Ho, W., Yu, G., & Zhang, T. (2016). Probiotics for prevention and treatment of respiratory tract infections in children: A systematic review and meta-analysis of randomized controlled trials. Medicine, 95(31). http://journals.lww.com/md-journal/Fulltext/2016/08020/Polymorphism_in_the_IL4R_gene_and_clinical.90.aspx
39. Wigham, S., Ternent, L., Bryant, A., Robalino, S., Sniehotta, F. F., & Adams, J. (2014). Parental financial incentives for increasing preschool vaccination uptake: Systematic review. Pediatrics, 134(4), E1117–E1128.
40. Willmott, M., Nicholson, A., Busse, H., MacArthur, G. J., Brookes, S., & Campbell, R. (2015). Effectiveness of hand hygiene interventions in reducing illness absence among children in educational settings: A systematic review and meta-analysis. Archives of Disease in Childhood.
41. Yakoob, M. Y., Salam, R. A., Khan, F. R., & Bhutta, Z. A. (2016). Vitamin D supplementation for preventing infections in children under five years of age. Cochrane Database of Systematic Reviews, 11, CD008824.
42. Zhang, G., Hu, H., Liu, C., Shristi, S., & Li, Z. (2016). Probiotics for preventing late-onset sepsis in preterm neonates: A PRISMA-compliant systematic review and meta-analysis of randomized controlled trials. Medicine, 95(8). http://journals.lww.com/md-journal/Fulltext/2016/02230/Probiotics_for_Preventing_Late_Onset_Sepsis_in.14.aspx
43. Zhang, L., Prietsch, S. O. M., Axelsson, I., & Halperin, S. A. (2014). Acellular vaccines for preventing whooping cough in children. Cochrane Database of Systematic Reviews, 9. http://dx.doi.org/10.1002/14651858.CD001478.pub6
44. Zhu, T., Zhang, C., Yu, L., Chen, J., Qiu, H., Lyu, W., & al, et. (2015). The preventive effect of vaccine prophylaxis on severe respiratory syncytial virus infection: A meta-analysis. Virologica Sinica, 30(5), 371–378.

# The publication only includes interventions targeting adults, pregnant women, adolescents or children older than 5 years old; or fail to synthesize primary studies’ results for the under-5 age group separately.

1. Adetokunboh, O. O., Ndwandwe, D., Awotiwon, A., Uthman, O. A., & Wiysonge, C. S. (2019). Vaccination among HIV-infected, HIV-exposed uninfected and HIV-uninfected children: A systematic review and meta-analysis of evidence related to vaccine efficacy and effectiveness. Human Vaccines & Immunotherapeutics, 1–12.
2. Alves Galvão, M. G., Rocha Crispino Santos, M. A., & Alves da Cunha, A. J. L. (2014). Amantadine and rimantadine for influenza A in children and the elderly. Cochrane Database of Systematic Reviews, 2014(11), Art. No.: CD002745.
3. Ambia, J., & Mandala, J. (2016). A systematic review of interventions to improve prevention of mother-to-child HIV transmission service delivery and promote retention. Journal of the International AIDS Society, 19(1), 20309.
4. Andani, A., van Elten, T. M., Bunge, E. M., Marano, C., Salgado, F., & Jacobsen, K. H. (2020). Hepatitis A epidemiology in Latin American countries: A 2020 view from a systematic literature review. Expert Review of Vaccines, 19(9), 795–805.
5. Aya Pastrana, N., Lazo-Porras, M., Miranda, J. J., Beran, D., & Suggs, L. S. (2020). Social marketing interventions for the prevention and control of neglected tropical diseases: A systematic review. Plos Neglected Tropical Diseases, 14(6), 1–31.
6. Bastos, M. L., Menzies, D., Hone, T., Dehghani, K., & Trajman, A. (2017). The impact of the Brazilian family health strategy on selected primary care sensitive conditions: A systematic review. PLoS One, 12(8), e0182336–e0182336.
7. Benjamin-Chung, J., Abedin, J., Berger, D., Clark, A., Jimenez, V., Konagaya, E., Tran, D., Arnold, B. F., Hubbard, A. E., Luby, S. P., Miguel, E., & Colford, J. M. (2017). Spillover effects on health outcomes in low- and middle-income countries: A systematic review. International Journal of Epidemiology, 46(4), 1251–1276.
8. Bi, Q., Ferreras, E., Pezzoli, L., Legros, D., Ivers, L., Date, K., & al, et. (2017). Protection against cholera from killed whole-cell oral cholera vaccines: A systematic review and meta-analysis. The Lancet Infectious Diseases, DOI: 10.1016/S1473-3099(17)30359-6.
9. Carreira, H., Bastos, A., Peleteiro, B., & Lunet, N. (2015). Breast-feeding and Helicobacter pylori infection: Systematic review and meta-analysis. Public Health Nutrition, 18(3), 500–520.
10. Choi, L., Pryce, J., & Garner, P. (2019). Indoor residual spraying for preventing malaria in communities using insecticide‐treated nets. Cochrane Database of Systematic Reviews, 5. http://dx.doi.org/10.1002/14651858.CD012688.pub2
11. Cohen, A., & Colford, J. M. (2017). Effects of Boiling Drinking Water on Diarrhea and Pathogen-Specific Infections in Low- and Middle-Income Countries: A Systematic Review and Meta-Analysis. American Journal of Tropical Medicine and Hygiene, 97(5), 1362–1377.
12. da Silveira, L. T. C., Tura, B., & Santos, M. (2019). Systematic review of dengue vaccine efficacy. BMC: BioMed Central Infectious Diseases, 19(1), 750.
13. De Tomasi, K., & Mouala, C. (2019). Good practices for retention in the circuit of prevention of Mother-Child Transmission of HIV in Sub-Saharan Africa: A systematic review of the literature. Bonnes Pratiques Pour Le Maintien Dans Le Circuit de La Prevention de La Transmission Du VIH de La Mere a l’enfant (PTME) En Afrique Subsaharienne : Une Revue Systematique de La Litterature., 29(3), 279–286.
14. Dol, J., Campbell-Yeo, M., Tomblin Murphy, G., Aston, M., McMillan, D., & Richardson, B. (2019). Impact of mobile health interventions during the perinatal period for mothers in low- and middle-income countries: A systematic review protocol. JBI Database Of Systematic Reviews And Implementation Reports, 17(2), 137–146.
15. Ejemot-Nwadiaro, R. I., Ehiri, J. E., Arikpo, D., Meremikwu, M. M., & Critchley, J. A. (2015). Hand washing promotion for preventing diarrhoea. Cochrane Database of Systematic Reviews, 9, CD004265.
16. Ejere, H. O., Alhassan, M. B., & Rabiu, M. (2015). Face washing promotion for preventing active trachoma. Cochrane Database of Systematic Reviews, 2, CD003659.
17. Faber, T., Kumar, A., Mackenbach, J., Millett, C., Basu, S., Sheikh, A., & al, et. (2017). Effect of tobacco control policies on perinatal and child health: A systematic review and meta-analysis. The Lancet Public Health, 2(9), e420–e437.
18. Firestone, R., Rowe, C. J., Modi, S. N., & Sievers, D. (2017). The effectiveness of social marketing in global health: A systematic review. Health Policy & Planning, 32(1), 110–124.
19. Furnival-Adams, J., Olanga, E. A., Napier, M., & Garner, P. (2020). House modifications for preventing malaria. The Cochrane Database of Systematic Reviews, 10, CD013398.
20. Gera, T., Shah, D., & Sachdev, H. S. (2018). Impact of Water, Sanitation and Hygiene Interventions on Growth, Non-diarrheal Morbidity and Mortality in Children Residing in Low- and Middle-income Countries: A Systematic Review. Indian Pediatrics, 55(5), 381–393.
21. Gonzalez, U., Pinart, M., Sinclair, D., Firooz, A., Enk, C., Velez, I. D., Esterhuizen, T. M., & et al. (2015). Vector and reservoir control for preventing leishmaniasis. Cochrane Database of Systematic Reviews, 8, CD008736.
22. Gopalan, S. S., Mutasa, R., Friedman, J., & Das, A. (2014). Health sector demand-side financial incentives in low- and middle-income countries: A systematic review on demand- and supply-side effects. Social Science & Medicine, 100, 72–83.
23. Heijnen, M., Cumming, O., Peletz, R., Chan, G., Brown, J., Baker, K., & et al. (2014). Shared sanitation versus individual household latrines: A systematic review of health outcomes. PLoS ONE, 9(4), e93300.
24. Heneghan, C. J., Onakpoya, I., Thompson, M., Spencer, E. A., Jones, M., & Jefferson, T. (2014). Zanamivir for influenza in adults and children: Systematic review of clinical study reports and summary of regulatory comments. Bmj, 348, g2547.
25. Henson, R. M., Ortigoza, A., Martinez-Folgar, K., Baeza, F., Caiaffa, W., Vergara, A. V., Roux, A. V. D., & Lovasi, G. (2020). Evaluating the health effects of place-based slum upgrading physical environment interventions: A systematic review (2012-2018). Social Science & Medicine, 261, 13.
26. Imdad, A., Mayo‐Wilson, E., Herzer, K., & Bhutta, Z. A. (2017). Vitamin A supplementation for preventing morbidity and mortality in children from six months to five years of age. Cochrane Database of Systematic Reviews, 3.
27. Jefferson, T., Del Mar, C. B., Dooley, L., Ferroni, E., Al-Ansary, L. A., Bawazeer, G. A., van Driel, M. L., Jones, M. A., Thorning, S., Beller, E. M., Clark, J., Hoffmann, T. C., Glasziou, P. P., & Conly, J. M. (2020). Physical interventions to interrupt or reduce the spread of respiratory viruses. The Cochrane Database of Systematic Reviews, 11, CD006207.
28. Jin, H., Tan, Z., Zhang, X., Zhao, Y., Wang, B., & Liu, P. (2014). Immunization interventions to interrupt hepatitis B virus mother-to-child transmission: A meta-analysis of randomized controlled trials. BMC Pediatrics, 14, 307.
29. Kakuru, A., Staedke, S. G., Dorsey, G., Rogerson, S., & Chandramohan, D. (2019). Impact of Plasmodium falciparum malaria and intermittent preventive treatment of malaria in pregnancy on the risk of malaria in infants: A systematic review. Malaria Journal, 18(1), 304.
30. Lassi, Z. S., Fahad, R., Irfan, O., Rabia, H., Das, J. K., & Bhutta, Z. A. (2020). Impact of infant and young child feeding (ITCF) nutrition interventions on breastfeeding practices, growth and mortality in low- and middle-income countries: Systematic review. Nutrients, 12(3). https://www.mdpi.com/2072-6643/12/3/722
31. Maia Mf, K. M. R. M. L. C. M. S. J. (2018). Mosquito repellents for malaria prevention. Cochrane Database of Systematic Reviews, 2, CD011595.
32. Malisheni, M., Khaiboullina, S. F., Rizvanov, A. A., Takah, N., Murewanhema, G., & Bates, M. (2017). Clinical Efficacy, Safety, and Immunogenicity of a Live Attenuated Tetravalent Dengue Vaccine (CYD-TDV) in Children: A Systematic Review with Meta-analysis. Frontiers in Immunology, 8, 863.
33. Manley, J., & Slavchevska, V. (2019). Are cash transfers the answer for child nutrition in sub-Saharan Africa? A literature review. Themed Issue: Health and Well-Being., 37(2), 204–224.
34. Mbeye, N. M., ter Kuile, F. O., Davies, M. A., Phiri, K. S., Egger, M., Wandeler, G., & Ie, D. E. A. S. A. (2014). Cotrimoxazole prophylactic treatment prevents malaria in children in sub-Saharan Africa: Systematic review and meta-analysis. Tropical Medicine & International Health : TM & IH, 19(9), 1057–1067.
35. Mitra, S., Palmer, M., Pullaro, S., Mont, D., & Groce, N. (2017). Health Insurance and Children in Low- and Middle-income Countries: A Review. Economic Record, 93(302), 484–500.
36. Mohd Faizal, M., Anaanthan, B. P., Nur Ezdiani, M., Azmawati, M. N., Norfazilah, A., Hasanain, F. G., Jeffree, M. S., Syed Abdul Rahim, S. S., & Hassan, M. R. (2020). Efficacy of albendazole against soil-transmitted helminthiasiamong children in asia: Systematic review. Open Access Macedonian Journal of Medical Sciences, 8, 70–77.
37. Morimoto, N., & Takeishi, K. (2018). Change in the efficacy of influenza vaccination after repeated inoculation under antigenic mismatch: A systematic review and meta-analysis. Vaccine, 36(7), 949–957.
38. Oliveira, J., Allert, R., & East, C. (2016). Vitamin A supplementation for postpartum women. The Cochrane Database of Systematic Reviews, 2016(3), CD005944.
39. Orr, J. A., & King, R. J. (2015). Mobile phone SMS messages can enhance healthy behaviour: A meta-analysis of randomised controlled trials. Health Psychology Review, 9(4), 397–416.
40. Owusu-Addo, E., & Owusu-Addo, S. B. (2014). Effectiveness of health education in community-based malaria prevention and control interventions in sub-Saharan Africa: A systematic review. Journal of Biology, Agriculture and Healthcare, 4(3), 22–34.
41. Ozen, M., Sandal, G., & Dinleyici, E. (2015). Probiotics for the prevention of pediatric upper respiratory tract infections: A systematic review. Expert Opinion on Biological Therapy, 15(1), 9–20.
42. Pega, F., Liu, S. Y., Walter, S., Pabayo, R., Saith, R., & Lhachimi, S. K. (2017). Unconditional cash transfers for reducing poverty and vulnerabilities: Effect on use of health services and health outcomes in low‐ and middle‐income countries. Cochrane Database of Systematic Reviews, 11. https://doi.org/10.1002/14651858.CD011135.pub2
43. Peña‐Rosas, J. P., Mithra, P., Unnikrishnan, B., Kumar, N., De‐Regil, L. M., Nair, N. S., Garcia‐Casal, M. N., & Solon, J. A. (2019). Fortification of rice with vitamins and minerals for addressing micronutrient malnutrition. Cochrane Database of Systematic Reviews, 10. http://dx.doi.org/10.1002/14651858.CD009902.pub2
44. Pryce, J., Richardson, M., & Lengeler, C. (2018). Insecticide‐treated nets for preventing malaria. Cochrane Database of Systematic Reviews, 11. http://dx.doi.org/10.1002/14651858.CD000363.pub3
45. Quansah, R., Semple, S., Ochieng, C. A., Juvekar, S., Armah, F. A., Luginaah, I., & Emina, J. (2017). Effectiveness of interventions to reduce household air pollution and/or improve health in homes using solid fuel in low-and-middle income countries: A systematic review and meta-analysis. Environment International, 103, 73–90.
46. Ramesh, A., Blanchet, K., Ensink, J. H., & Roberts, B. (2015). Evidence on the effectiveness of water, sanitation, and hygiene (WASH) interventions on health outcomes in humanitarian crises: A systematic review. PLoS ONE, 10(9).
47. Salam, R. A., Das, J. K., Lassi, Z. S., & Bhutta, Z. A. (2014). Impact of community-based interventions for the prevention and control of malaria on intervention coverage and health outcomes for the prevention and control of malaria. Infectious Diseases of Poverty, 3(25). http://www.ncbi.nlm.nih.gov/pmc/articles/PMC4128612/
48. Salam, R. A., Maredia, H., Das, J. K., Lassi, Z. S., & Bhutta, Z. A. (2014). Community-based interventions for the prevention and control of helmintic neglected tropical diseases. Infectious Diseases of Poverty, 3(23).
49. Schiavo, R., Leung, M. M., & Brown, M. (2014). Communicating risk and promoting disease mitigation measures in epidemics and emerging disease settings. Pathogens and Global Health, 108(2), 76–94.
50. Schwerdtle, P., Onekon, C. K., & Recoche, K. (2018). A quantitative systematic review and meta-analysis of the effectiveness of oral cholera vaccine as a reactive measure in cholera outbreaks. Prehospital & Disaster Medicine, 33(1), 2–6.
51. Scott, A. M., Clark, J., Julien, B., Islam, F., Roos, K., Grimwood, K., & et al. (2019). Probiotics for preventing acute otitis media in children. The Cochrane Database of Systematic Reviews, 2019(1), CD012941.
52. Shiri, T., Datta, S., Madan, J., Tsertsvadze, A., Royle, P., Keeling, M. J., McCarthy, N. D., & Petrou, S. (2017). Indirect effects of childhood pneumococcal conjugate vaccination on invasive pneumococcal disease: A systematic review and meta-analysis. The Lancet Global Health, 5(1), e51–e59.
53. Takah, N. F., Atem, J. A., Aminde, L. N., Malisheni, M., & Murewenhema, G. (2018). Male partner involvement in increasing the uptake of infant antiretroviral prophylaxis/treatment in sub Saharan Africa: A systematic review and meta-analysis. BMC Public Health, 18(1), 249.
54. Taylor, D. L., Kahawita, T. M., Cairncross, S., & Ensink, J. H. (2015). The impact of water, sanitation and hygiene interventions to control cholera: A systematic review. PLoS ONE, 10(8), e0135676.
55. Veroniki, A. A., Antony, J., Straus, S. E., Ashoor, H. M., Finkelstein, Y., Khan, P. A., & al, et. (2018). Comparative safety and effectiveness of perinatal antiretroviral therapies for HIV-infected women and their children: Systematic review and network meta-analysis including different study designs. PloS One, 13(6), e0198447.
56. Welch, Vivian A., Ghogomu, E., Hossain, A., Awasthi, S., Bhutta, Z., Cumberbatch, C., Fletcher, R., McGowan, J., Krishnaratne, S., Kristjansson, E., Sohani, S., Suresh, S., Tugwell, P., White, H., & Wells, G. (2016). Deworming and adjuvant interventions for improving the developmental health and well-being of children in low- and middle-income countries: A systematic review and network meta-analysis. Campbell Systematic Reviews, 12(1), 1–383.
57. Welch, Vivian Andrea, Hossain, A., Ghogomu, E., Riddle, A., Cousens, S., Gaffey, M., Arora, P., Black, R., Bundy, D., Castro, M. C., Chen, L., Dewidar, O., Elliott, A., Friis, H., Hollingsworth, T. D., Horton, S., King, C. H., Thi, H. L., Liu, C., … Wells, G. A. (2019). Deworming children for soil-transmitted helminths in low and middle-income countries: Systematic review and individual participant data network meta-analysis. Journal of Development Effectiveness, 11(3), 288–306.
58. Whitford, K., Liu, B., Micallef, J., Yin, J. K., Macartney, K., Damme, P. van, & Kaldor, J. M. (2018). Long-term impact of infant immunization on hepatitis B prevalence: A systematic review and meta-analysis. Bulletin of the World Health Organization, 96(7), 484–497.
59. Wilson, A. L., Chen-Hussey, V., Logan, J. G., & Lindsay, S. W. (2014). Are topical insect repellents effective against malaria in endemic populations? A systematic review and meta-analysis. Malaria Journal, 13(1), 446–454.
60. Wilson, A., Dhiman, R., Kitron, U., Scott, T., van den Berg, H., & Lindsay, S. (2014). Benefit of insecticide-treated nets, curtains and screening on vector borne diseases, excluding malaria: A systematic review and meta-analysis. PLoS Neglected Tropical Diseases, 8(10), DOI: 10.1371/journal.pntd.0003228.
61. Wiysonge, C. S., Ndze, V. N., Kongnyuy, E. J., & Shey, M. S. (2017). Vitamin A supplements for reducing mother-to-child HIV transmission. Cochrane Database of Systematic Reviews, 9, CD003648.
62. Wolf, J., Hunter, P. R., Freeman, M. C., Cumming, O., Clasen, T., Bartram, J., Higgins, J. P., Johnston, R., Medlicott, K., & Boisson, S. (2018). Impact of drinking water, sanitation and handwashing with soap on childhood diarrhoeal disease: Updated meta‐analysis and meta‐regression. Tropical Medicine & International Health, 23(5), 508–525.
63. Wolfe, M., Kaur, M., Yates, T., Woodin, M., & Lantagne, D. (2018). A Systematic Review and Meta-Analysis of the Association between Water, Sanitation, and Hygiene Exposures and Cholera in Case-Control Studies. American Journal of Tropical Medicine and Hygiene, 99(2), 534–545.
64. Xiao, L., Xing, C., Yang, Z., Xu, S., Wang, M., Du, H., Liu, K., & al, et. (2015). Vitamin D supplementation for the prevention of childhood acute respiratory infections: A systematic review of randomised controlled trials. British Journal of Nutrition, 114(7), 1026–1034.
65. Yakoob, M. Y., Qadir, M., & Hany, O. E. (2018). Vitamin A supplementation for prevention and treatment of malaria during pregnancy and childhood: A systematic review and meta-analysis. Journal of Epidemiology and Global Health, 8(1), 20–28.
66. Yates, T., Vijcic, J., Joseph, M. L., & Lantagne, D. (2017). WASH interventions in disease outbreak response. Oxfam GB. https://oxfamilibrary.openrepository.com/bitstream/handle/10546/620202/rr-wash-interventions-disease-outbreak-280217-en.pdf;jsessionid=E76E4942D92E062E8C0BB0FF403BD31D?sequence=1
67. Zimmermann, P., Finn, A., & Curtis, N. (2018). Does BCG Vaccination Protect Against Nontuberculous Mycobacterial Infection? A Systematic Review and Meta-Analysis. Journal of Infectious Diseases, 218(5), 679–687.

# The publication only includes curative interventions or secondary or tertiary prevention interventions; is not addressing or affecting the burden of infectious diseases or their risk factors; or only report trends in individual behaviors without any actions aiming at changing or influencing them.

1. Adeloye, D., Jacobs, W., Amuta, A. O., Ogundipe, O., Mosaku, O., Gadanya, M. A., & Oni, G. (2017). Coverage and determinants of childhood immunization in Nigeria: A systematic review and meta-analysis. Vaccine, 35(22), 2871–2881.
2. Bwana, V. M., Frimpong, C., Simulundu, E., Mfinanga, S. G., Mboera, L. E. G., & Michelo, C. (2016). Accessibility of services for early infant diagnosis of human immunodeficiency virus in Sub-Saharan Africa: A systematic review. Tanzania Journal of Health Research, 18(3).
3. Garcia-Elorrio, E., Rowe, S. Y., Teijeiro, M. E., Ciapponi, A., & Rowe, A. K. (2019). The effectiveness of the quality improvement collaborative strategy in low- and middle-income countries: A systematic review and meta-analysis. Plos One, 14(10), 23.
4. Jefferson, T., Jones, M., Doshi, P., Spencer, E. A., Onakpoya, I., & Heneghan, C. J. (2014). Oseltamivir for influenza in adults and children: Systematic review of clinical study reports and summary of regulatory comments. BMJ: British Medical Journal, 348, g2545.
5. Jonesteller, C. L., Burnett, E., Yen, C., Tate, J. E., & Parashar, U. D. (2017). Effectiveness of Rotavirus Vaccination: A Systematic Review of the First Decade of Global Postlicensure Data, 2006-2016. Clinical Infectious Diseases, 65(5), 840–850.
6. Kristjansson, E., Francis, D., Liberato, S., Greenhalgh, T., Welch, V., Benkhalti Jandu, M., Batal, M., Rader, T., Noonan, E., Janzen, L., Shea, B., A Wells, G., & Petticrew, M. (2016). Supplementary feeding for improving the health of disadvantaged infants and young children. 3ie Systematic Review, 15. http://3ieimpact.org/evidence-hub/publications/systematic-reviews/supplementary-feeding-improving-health-disadvantaged
7. Lassi, Z. S., & Bhutta, Z. A. (2015). Community‐based intervention packages for reducing maternal and neonatal morbidity and mortality and improving neonatal outcomes. Cochrane Database of Systematic Reviews, 3. http://dx.doi.org/10.1002/14651858.CD007754.pub3
8. Lee, M. K., & Binns, C. (2019). Breastfeeding and the Risk of Infant Illness in Asia: A Review. International Journal of Environmental Research and Public Health, 17(1). http://ovidsp.ovid.com/ovidweb.cgi?T=JS&PAGE=reference&D=medl&NEWS=N&AN=31888064
9. Milligan, R., Paul, M., Richardson, M., & Neuberger, A. (2018). Vaccines for preventing typhoid fever. Cochrane Database of Systematic Reviews, 5, CD001261.
10. Molina, E., Carella, L., Pacheco, A., Cruces, G., & Gasparini, L. (2016). Community monitoring interventions to curb corruption and increase access and quality of service delivery in low- and middle-income countries: A systematic review. Campbell Systematic Reviews, 12(1), 1–204.
11. Pratley, P. (2016). Associations between quantitative measures of women’s empowerment and access to care and health status for mothers and their children: A systematic review of evidence from the developing world. Social Science & Medicine, 169, 119–131. https://doi.org/10.1016/j.socscimed.2016.08.001
12. Richardson, D. (2018). Key Findings on Families, Family Policy and the Sustainable Development Goals: Synthesis Report. UNICEF Innocenti Research Centre. https://www.unicef-irc.org/publications/948-key-findings-on-families-family-policy-and-the-sustainable-development-goals-synthesis.html
13. Sankar, M. J., Sinha, B., Chowdhury, R., Bhandari, N., Taneja, S., Martines, J., & Bahl, R. (2015). Optimal breastfeeding practices and infant and child mortality: A systematic review and meta-analysis. Acta Paediatrica, 104(467), 3–13.
14. Sharma, D., Shastri, S., & Sharma, P. (2017). Role of lactoferrin in neonatal care: A systematic review. Journal of Maternal-Fetal & Neonatal Medicine, 30(16), 1920–1932.
15. Whidden, C., Thwing, J., Gutman, J., Leyrat, C., Kayentao, K., Johnson, A., Greenwood, B., & Chandramohan, D. (2019). A systematic review of proactive case detection by community health workers for the management of common childhood illnesses. American Journal of Tropical Medicine and Hygiene, 101(5), 21.
16. Whidden, Caroline, Thwing, J., Gutman, J., Wohl, E., Leyrat, C., Kayentao, K., Johnson, A. D., Greenwood, B., & Chandramohan, D. (2019). Proactive case detection of common childhood illnesses by community health workers: A systematic review. BMJ Global Health, 4(6), e001799.

# The publication does not include a relevant overall health outcome or disaggregated information by or between population groups.

1. Aaby, P., Ravn, H., Benn, C. S., Rodrigues, A., Samb, B., Ibrahim, S. A., Libman, M. D., & Whittle, H. C. (2016). Randomized Trials Comparing Inactivated Vaccine After Medium-or High-titer Measles Vaccine With Standard Titer Measles Vaccine After Inactivated Vaccine. The Pediatric Infectious Disease Journal, 35(11), 1232–1241.
2. Adegbosin, A. E., Zhou, H., Wang, S., Stantic, B., & Sun, J. (2019). Systematic review and meta-analysis of the association between dimensions of inequality and a selection of indicators of Reproductive, Maternal, Newborn and Child Health (RMNCH). Journal of Global Health, 9(1), 010429.
3. Adinew, Y. M., Feleke, S. A., Mengesha, Z. B., & Workie, S. B. (2017). Childhood mortality: Trends and determinants in Ethiopia from 1990 to 2015—A systematic review. Advances in Public Health, 7479295(42). https://www.hindawi.com/journals/aph/2017/7479295/
4. Arrivillaga, M., & Salcedo, J. P. (2014). A SYSTEMATIC REVIEW OF MICROFINANCE-BASED INTERVENTIONS FOR HIV/AIDS PREVENTION. Aids Education and Prevention, 26(1), 13–27.
5. Athuman, M., Kabanywanyi, A. M., & Rohwer, A. C. (2015). Intermittent preventive antimalarial treatment for children with anaemia. Cochrane Database of Systematic Reviews, 1, CD010767.
6. Austin, A., Langer, A., Salam, R. A., Lassi, Z. S., Das, J. K., & Bhutta, Z. A. (2014). Approaches to improve the quality of maternal and newborn health care: An overview of the evidence. Reproductive Health, 11, S1.
7. Balhara, K. S., Silvestri, D. M., Tyler Winders, W., Selvam, A., Kivlehan, S. M., Becker, T. K., Levine, A. C., & Global Emergency Medicine Literature Review, G. (2017). Impact of nutrition interventions on pediatric mortality and nutrition outcomes in humanitarian emergencies: A systematic review. Tropical Medicine & International Health, 22(12), 1464–1492.
8. Bassey, C., Crooks, H., Paterson, K., Ball, R., Howell, K., Humphries-Cuff, I., Gaffigan, K., Rao, N., Whitty, J. A., & Hooper, L. (2020). Impact of home food production on nutritional blindness, stunting, wasting, underweight and mortality in children: A systematic review and meta-analysis of controlled trials. Critical Reviews in Food Science and Nutrition. https://www.scopus.com/inward/record.uri?eid=2-s2.0-85097096578&doi=10.1080%2f10408398.2020.1848786&partnerID=40&md5=1cbd51a8849cbe84459ef713c7753303
9. Beirne, P. V., Hennessy, S., Cadogan, S. L., Shiely, F., Fitzgerald, T., & MacLeod, F. (2018). Needle size for vaccination procedures in children and adolescents. Cochrane Database of Systematic Reviews, 8, CD010720.
10. Brown, T. W., Van Urk, F. C., Waller, R., & Mayo-Wilson, E. (2014). Centre-based day care for children under five in low- and middle-income countries. Cochrane Database of Systematic Reviews, 2014(9). https://www.cochranelibrary.com/cdsr/doi/10.1002/14651858.CD010543.pub2/full
11. Buckley, B. S., Henschke, N., Bergman, H., Skidmore, B., Klemm, E. J., Villanueva, G., & et al. (2019). Impact of vaccination on antibiotic usage: A systematic review and meta-analysis. Clinical Microbiology and Infection : The Official Publication of the European Society of Clinical Microbiology and Infectious Diseases, 25(10), 1213–1225.
12. Byrne, A., Hodge, A., Jimenez-Soto, E., & Morgan, A. (2014). What works? Strategies to increase reproductive, maternal and child health in difficult to access mountainous locations: A systematic literature review. PLoS ONE [Electronic Resource], 9(2), e87683.
13. Ehiri, J. E., Gunn, J. K., Center, K. E., Li, Y., Rouhani, M., & Ezeanolue, E. E. (2014). Training and deployment of lay refugee/internally displaced persons to provide basic health services in camps: A systematic review. Glob Health Action, 7, 23902.
14. Farnsworth, S. K., Bose, K., Fajobi, O., Souza, P. P., Peniston, A., Davidson, L. L., Griffiths, M., & Hodgins, S. (2014). Community engagement to enhance child survival and early development in low- and middle-income countries: An evidence review. (Special Issue: Population-level behavior change to enhance child survival and development in low- and middle-income countries: A review of the evidence.). Journal of Health Communication: International Perspectives; 2014, 1. https://www.tandfonline.com/doi/full/10.1080/10810730.2014.941519
15. Fortanier, A. C., Venekamp, R. P., Boonacker, C. W., Hak, E., Schilder, A. G., Sanders, E. A., & Damoiseaux, R. A. (2020). Pneumococcal conjugate vaccines for preventing acute otitis media in children. Cochrane Database of Systematic Reviews, 4, Art. No.: CD001480.
16. Godfrey, O., Zhang, W., Amponsem-Boateng, C., Bonney Oppong, T., Zhao, Q., & Li, D. (2020). Evidence of rotavirus vaccine impact in sub-Saharan Africa: Systematic review and meta-analysis. PloS One, 15(4), e0232113.
17. Gogia, S., & Sachdev, H. P. (2016). Home-based neonatal care by community health workers for preventing mortality in neonates in low- and middle-income countries: A systematic review. Journal of Perinatology, 36, S55-73.
18. Hanson, C., Kujala, S., Waiswa, P., Marchant, T., & Schellenberg, J. (2017). Community-based approaches for neonatal survival: Meta-analyses of randomized trial data. (Special Theme: Measuring quality of care.). Bulletin of the World Health Organization, 95(6). http://www.who.int/bulletin/volumes/95/6/16-175844.pdf
19. James, N., Lawson, K., & Acharya, Y. (2020). Evidence on result-based financing in maternal and child health in low- and middle-income countries: A systematic review. Global Health Research and Policy, 5, 31.
20. Kabongo, E., Mukumbang, F., Delobelle, P., & Nicol, E. (2020). Explaining the impact of mHealth on maternal and child health care in low-and middle-income countries: A realist synthesis (rayyan-123018925).
21. Kalan, R., Wiysonge, C. S., Ramafuthole, T., Allie, K., Ebrahim, F., & Engel, M. E. (2014). Mobile phone text messaging for improving the uptake of vaccinations: A systematic review protocol. BMJ Open, 4(8), e005130.
22. Kikuchi, K., Ansah, E. K., Okawa, S., Enuameh, Y., Yasuoka, J., Nanishi, K., Shibanuma, A., Gyapong, M., Owusu-Agyei, S., Oduro, A. R., Asare, G. Q., Hodgson, A., Jimba, M., & Ghana, E. I. R. P. T. (2015). Effective Linkages of Continuum of Care for Improving Neonatal, Perinatal, and Maternal Mortality: A Systematic Review and Meta-Analysis. PLoS ONE [Electronic Resource], 10(9), e0139288.
23. le Roux, S. M., Abrams, E. J., Nguyen, K., & Myer, L. (2016). Clinical outcomes of HIV-exposed, HIV-uninfected children in sub-Saharan Africa. Tropical Medicine & International Health, 21(7), 829–845.
24. Lee, S. H., Nurmatov, U. B., Nwaru, B. I., Mukherjee, M., Grant, L., & Pagliari, C. (2016). Effectiveness of mHealth interventions for maternal, newborn and child health in low- and middle-income countries: Systematic review and meta-analysis. Journal of Global Health, 6(1), 010401.
25. Levy, J. K., Darmstadt, G. L., Ashby, C., Quandt, M., Halsey, E., Nagar, A., & Greene, M. E. (2020). Characteristics of successful programmes targeting gender inequality and restrictive gender norms for the health and wellbeing of children, adolescents, and young adults: A systematic review. The Lancet. Global Health, 8(2), e225–e236.
26. Majamanda, J., Maureen, D., Munkhondia, T. M., & Carrier, J. (2014). The Effectiveness of Community-Based Nutrition Education on the Nutrition Status of Under-five Children in Developing Countries. A Systematic Review. Malawi Medical Journal, 26(4), 115–118.
27. Murunga, N., P Otieno, G., Maia, M., & N Agoti, C. (2020). Effectiveness of Rotarix R vaccine in Africa in the first decade of progressive introduction, 2009-2019: Systematic review and meta-analysis. Wellcome Open Research, 5, 187.
28. Norhayati, M. N., Ho, J. J., & Azman, M. Y. (2017). Influenza vaccines for preventing acute otitis media in infants and children. Cochrane Database of Systematic Reviews, 10, CD010089.
29. Orton, L., Pennington, A., Nayak, S., Sowden, A., White, M., & Whitehead, M. (2016). Group-based microfinance for collective empowerment: A systematic review of health impacts. Bulletin of the World Health Organization, 94(9), 694-704A.
30. Paludan-Muller, A. S., Boesen, K., Klerings, I., Jorgensen, K. J., & Munkholm, K. (2020). Hand cleaning with ash for reducing the spread of viral and bacterial infections: A rapid review. The Cochrane Database of Systematic Reviews, 4, CD013597.
31. Pega, F., Liu, S. Y., Walter, S., & Lhachimi, S. K. (2015). Unconditional cash transfers for assistance in humanitarian disasters: Effect on use of health services and health outcomes in low‐ and middle‐income countries. Cochrane Database of Systematic Reviews, 9. http://dx.doi.org/10.1002/14651858.CD011247.pub2
32. Pollard, S. L., Malpica-Llanos, T., Friberg, I. K., Fischer-Walker, C., Ashraf, S., & Walker, N. (2015). Estimating the herd immunity effect of rotavirus vaccine. Vaccine, 33(32), 3795–3800.
33. Poorman, E., & Gazmararian, J. (2015). Use of text messaging for maternal and infant health: A systematic review of the literature. Maternal and Child Health Journal, 19(5), 969–989.
34. Qin, M., Hone, T., Millett, C., Moreno-Serra, R., McPake, B., Atun, R., & Lee, J. T. (2018). The impact of user charges on health outcomes in low-income and middle-income countries: A systematic review. (Special Issue: The Alma Ata declaration at 40: Reflections on primary health care in a new era.). BMJ Global Health, 3. https://gh.bmj.com/content/3/Suppl_3/e001087
35. Rosettie, K. L., Vos, T., Mokdad, A. H., Flaxman, A. D., Khalil, I., Troeger, C., & Weaver, M. R. (2018). Indirect Rotavirus Vaccine Effectiveness for the Prevention of Rotavirus Hospitalization: A Systematic Review and Meta-Analysis. American Journal of Tropical Medicine & Hygiene, 98(4), 1197–1201.
36. Siddiqi, A., Rajaram, A., & Miller, S. P. (2018). Do cash transfer programmes yield better health in the first year of life? A systematic review linking low-income/middle-income and high-income contexts. Archives of Disease in Childhood, 103(10), 920–926.
37. Soubeiga, D., Gauvin, L., Hatem, M. A., & Johri, M. (2014). Birth Preparedness and Complication Readiness (BPCR) interventions to reduce maternal and neonatal mortality in developing countries: Systematic review and meta-analysis. BMC Pregnancy & Childbirth, 14, 129.
38. Takah, N. F., Atem, J. A., Aminde, L. N., Malisheni, M., & Murewenhema, G. (2018). The impact of approaches in improving male partner involvement in the prevention of mother-to-child transmission of HIV on the uptake of safe infant feeding practices by HIV positive women in sub-Saharan Africa. A systematic review and meta-analysis. PLoS ONE [Electronic Resource], 13(12), e0207060.
39. Taylor‐Robinson, D. C., Maayan, N., Soares‐Weiser, K., Donegan, S., & Garner, P. (2015). Deworming drugs for soil‐transmitted intestinal worms in children: Effects on nutritional indicators, haemoglobin, and school performance. Cochrane Database of Systematic Reviews, 7. http://dx.doi.org/10.1002/14651858.CD000371.pub6
40. Tiruneh, G. T., Shiferaw, C. B., & Worku, A. (2019). Effectiveness and cost-effectiveness of home-based postpartum care on neonatal mortality and exclusive breastfeeding practice in low-and-middle-income countries: A systematic review and meta-analysis. BMC Pregnancy and Childbirth, 19(1), 507.
41. Tokhi, M., Comrie-Thomson, L., Davis, J., Portela, A., Chersich, M., & Luchters, S. (2018). Involving men to improve maternal and newborn health: A systematic review of the effectiveness of interventions. PLoS ONE [Electronic Resource], 13(1), e0191620.
42. Usuf, E., Bottomley, C., Adegbola, R. A., & Hall, A. (2014). Pneumococcal carriage in sub-Saharan Africa—A systematic review. PLoS ONE [Electronic Resource], 9(1), e85001.
43. Warren, E., Post, N., Hossain, M., Blanchet, K., & Roberts, B. (2015). Systematic review of the evidence on the effectiveness of sexual and reproductive health interventions in humanitarian crises. BMJ Open, 5(12), e008226.
44. Watterson, J. L., Walsh, J., & Madeka, I. (2015). Using mHealth to Improve Usage of Antenatal Care, Postnatal Care, and Immunization: A Systematic Review of the Literature. BioMed Research International, 2015, 153402.
45. Wessells, R., Stewart, C., Dewey, K., & Arnold, C. (2019). Effect of preventive lipid-based nutrient supplements provided to infants and young children 6 to 23 months of age on child mortality: A systematic review and meta-analysis. PROSPERO. http://www.crd.york.ac.uk/PROSPERO/display_record.php?ID=CRD42019128718
46. Zakiyah, N., Insani, W. N., Suwantika, A. A., van der Schans, J., & Postma, M. J. (2020). Pneumococcal Vaccination for Children in Asian Countries: A Systematic Review of Economic Evaluation Studies. Vaccines, 8(3). http://ovidsp.ovid.com/ovidweb.cgi?T=JS&PAGE=reference&D=prem6&NEWS=N&AN=32751569
47. Zeng, H., Chow, E. P. F., Zhao, Y., Wang, Y., Tang, M., Li, L., Tang, X., Liu, X., Zhong, Y., Wang, A., Lo, Y., & Zhang, L. (2016). Prevention of mother-to-child HIV transmission cascade in China: A systematic review and meta-analysis. Sexually Transmitted Infections, 92(2), 116–123.
