# Supplementary material for: Which public health interventions are effective in reducing morbidity, mortality and health inequalities from infectious diseases amongst children in low- and middle-income countries (LMICs): An umbrella review
Source: PLoS One. 2021 Jun 10;16(6):e0251905. doi: 10.1371/journal.pone.0251905 (PMC8191901; doi:10.1371/journal.pone.0251905)
Supplement: S9 Appendix — (DOCX) [file pone.0251905.s009.docx]

# S9 Appendix - Description of included reviews

| Review | Number and type of relevant studies /Number of studies covered in the review | Population and context | Control/Comparison groups | Interventions | Results | Funding of the review | AMSTAR2 |
| --- | --- | --- | --- | --- | --- | --- | --- |
| (Ali et al., 2018)  [72] | 2  (before-and-after studies)  /48 | Children aged 1–59 months in South Asia across sentinel surveillance hospitals in cities of Pakistan and Bangladesh | Pre-introduction period | Introduction of new HiB vaccines into national programs | HiB incidence in children aged 1–59 months declined within two years of national implementation of the vaccine.  The studies met two out of three of the quality criteria used in the authors’ quality assessment. | None | Critically Low |
| (Augustincic Polec et al., 2015)  [51] | 3  (RCT, cluster RCT, controlled before/after study)  /10 | Women of reproductive age or heads of households with children under 5 living in rural areas in Ghana, Ethiopia, Zambia | Controls received no malaria education/training or received an alternative education intervention | Education intervention delivered through the community on the use of insecticide treated nets (ITNs) | Educational interventions may be effective in increasing under-5 use of ITNs (low certainty; mixed quality and heterogeneity between included studies). | Knowledge Synthesis Grant, CIHR, Canada | High |
| (Bonner et al., 2015)  [73] | 3 (retrospective study, matched case-control)  /18 | Children between 12–60 months in Brazil and Uruguay | Children in the same age group or areas who did not receive vaccination; children before they received vaccination | Catch-up dose of PCV vaccine after its introduction into national programs | Significant vaccine efficacy in either the most prevalent serotype or across serotypes for disease incidence of Invasive Pneumococcal Disease.  The studies included in the review were of high quality, but the heterogeneity of the outcomes prevented a meta-analysis. | None | Critically Low |
| (Bright et al., 2017)  [65] | 27 (cluster RCT, controlled Before-and-After)  /57 | Infants and children under 5 years, healthcare workers involved in immunization services, caregivers and households with children under 5 living in 18 LMIC | Controls from the same area receiving no intervention, standard care/usual services, or an alternative or partial intervention | Supply-, demand- side or combined interventions to increase access to health services among children aged ≤5 years, including: delivering services at or closer to home and service level improvements for the supply-side interventions ;health promotion/education programs, text messages and financial or other incentives for the demand-side interventions. | For supply-side interventions aiming at improving immunization uptake (9 studies), as many studies found positive effect as those finding no effect.  For demand-side interventions (18 studies), half of the studies found no effect and a third found a positive effect.  There was strong variation in the effectiveness and the quality of evidence available for the different types of interventions.  The quality of the included studies was generally mixed, with the author urging for caution in the interpretation of the results. | CBM | Low |
| (Carroll et al., 2017)  [103] | 2  (cross-sectional study)  /11 | Children of both sexes between 6 and 59 months old living in conflict settings or resettlement or refugee camps in Bhutan, Nepal and Bangladesh | Children of the same age in different refugee or post-conflict populations or the known national or community prevalence | Point-of-use micronutrient powder (MNP) distribution programs: MNP single-dose sachet containing a combination of multiple vitamins and minerals designed to be mixed in the children’s homebased foods just before consumption | The intervention was associated with lower prevalence of diarrhea.  Overall low quality of the included studies. | None | Critically low |
| (Chavers et al., 2018)  [74] | 21 (case-control studies, time series analysis, observational studies)  /51 | Children under 5 in 8 Latin American countries (Colombia; El Salvador; Bolivia; Nicaragua; Brazil; Mexico; Panama; Venezuela) | Pre-introduction period | Introduction of new rotavirus vaccines into national programs | Vaccine is effective in reducing acute gastroenteritis morbidity (7 studies) and mortality (14 studies) in children under 5.  The authors provided limited information on the quality and design of the studies they included.  Children under 1 – the group carrying the greatest burden of rotavirus – benefitting most from vaccine effectiveness  High child mortality countries tended to have a lower vaccine effectiveness than those with lower child mortality. | None | Critically low |
| (Clasen et al., 2015)  [52] | 21  (RCTs, controlled before-after studies)  /55 | Households with children <5 years age in rural and urban settings as well as informal settings and refugee camps in 17 countries | Households or communities receiving no intervention or those receiving a ‘dummy’ intervention. | Improving water quality through water supply infrastructure improvement or point of use improvement such as water treatment and storage | For water supply interventions (4 studies), there was insufficient and mainly very low-quality evidence on what may or may not be effective in different settings.  For point-of-use interventions (18 studies), some interventions appeared to be protective against diarrhea in children across various settings (low to moderate quality evidence). | Liverpool School of Tropical Medicine, UK; Department for International Development (DFID), UK | High |
| (Cleminson & McGuire, 2016)  [53] | 6  (RCTs)  /21 | Preterm infants in hospital settings in Turkey, Bangladesh, India, Pakistan, Brazil | Preterm infants in hospital settings receiving routine skin care or alternative emollient | Topical application of emollients to prevent invasive infection | None of the meta-analyses show a statistically significant risk difference in the incidence of invasive infections (low to moderate quality evidence).  In the meta-analysis comparing either emollient against routine care, the authors also noted moderate to high heterogeneity across studies. | Hull York Medical School and Centre for Reviews and Dissemination, University of York, UK; National Institute of Health Research (NIHR) - Grant (13/89/12);  Eunice Kennedy Shriver National Institute of Child Health and Human Development National Institutes of Health, Department of Health and Human Services, USA -Contract No. HHSN275201100016C | High |
| (Conde-Agudelo & Diaz-Rossello, 2016)  [54] | 7  (RCTs)  /21 | Low-birth-weight infants – regardless of gestational age – in hospital settings in India, Colombia, Ecuador and Madagascar | Infants receiving conventional neonatal care or an alternative intervention (late vs. early-onset of the intervention) | Kangaroo mother care (KMC) provided by the parents and/or healthcare professionals | Positive effect of KMC on the risks of severe illness, nosocomial infection/sepsis and lower respiratory tract diseases but no effect on mild/moderate infection and illness or diarrhea risks at six months (moderate, mixed quality evidence, 6 studies).  No difference in morbidity between early- and late-onset of KMC (1 high quality trial). | Perinatology Research Branch, Eunice Kennedy Shriver National Institute of Child Health and Human Development/National Institutes of Health/Department of Health and Human Services, Bethesda, MD, and Detroit, MI, and Department of Obstetrics and Gynecology, Wayne State University, Detroit, MI, USA; Departamento de Neonatología del Hospital de Clínicas, Universidad de la República, Montevideo, Uruguay; Eunice Kennedy Shriver National Institute of Child Health and Human Development, National Institutes of Health, Department of Health and Human Services, USA - Contract No. HHSN275201100016C; National Institute for Health Research, UK - Cochrane Programme Grant (13/89/12) | High |
| (Crocker-Buque et al., 2017)  [75] | 10  (RCTs, before-after studies, time series analysis, evaluation, cross-sectional studies)  /63 | Children or households with children under 5 living in a low-income urban areas, slums, urban poor communities with slum-like characteristics, in Bangladesh, Pakistan, Guatemala, Uganda, Zambia, India | Target population before the intervention or control population from the same area. | Reminder/recalls systems about immunization; outreach services; and parent education about immunization | All studies show varying levels of success (low to moderate quality of the studies available)  The authors highlight the need for further research to confirm these findings. | Royal  Society of Tropical Medicine and Hygiene | Critically low |
| (Darvesh et al., 2017)  [76] | 44  (RCTs, cluster RCTs, quasi-experimental studies with controls)  /44 | Children under 5 in a community setting in 24 LMICs. Emergency settings, refugee camps or specific sub-populations (e.g. HIV positive people) were excluded | Children under 5 living communities receiving no intervention | Water quality improvement at source and point-of-use, the promotion of handwashing with soap and the safe disposal of excreta | Point-of-use water quality improvement interventions (32 studies) and hand washing promotion with soap (6 studies) reduce the risk of childhood diarrhea.  The evidence on water supply interventions at source (5 studies) and safe disposal of excreta (1 study) is insufficient to conclude on an effect of these intervention on diarrhea risks.  Overall, low to very low quality of evidence and high heterogeneity of results in the meta-analyses. | Bill & Melinda Gates Foundation (JHU Grant 115,621, Award  Number OPP1084423 for the “Development and Use of the Lives Saved  Tool (LiST)”) | Critically low |
| (de Oliveira et al., 2016)  [55] | 5  (controlled before and after studies, interrupted time series studies, case-control study)  /22 | Children under 5 years old in Brazil, Chile and Peru. | Pre-introduction period | Introduction of PCV-10 vaccines in national childhood immunization programs without any distinction of schedule or catch-up period established in the country | PCV introduction has a positive impact on pneumonia (3 studies) and meningitis (2 studies) mortality rates among under 5-year-old children.  PCVs have higher effectiveness on pneumococcal meningitis deaths for younger children (< 12 months of age) – the group with the higher burden of disease - as compared to older children (13 to 23 months old) (2 studies).  The authors flag out concerns related to study design, heterogeneity between studies and potential publication bias | Pan  American Health Organization and the Sabin  Vaccine Institute. | High |
| (DeAntonio et al., 2019)  [77] | 13  (observational and intervention studies)  /23 | Children under 5 living in Brazil, Chile, Uruguay, Argentina, Nicaragua, Panama and Mexico | Pre-introduction å  period | Introduction of Hib, rotavirus and PCV vaccines into national programs, with or without catch-up programs | The introduction of these vaccines reduced both the incidence of disease and mortality in children within the age group targeted for vaccination (quality assessment not available).  Possible herd protection effects in children under 5 not targeted by the vaccination program from routine Hib and rotavirus vaccine (5 studies). The authors assess the evidence as currently insufficient to confirm such an effect. | GlaxoSmithKline GSK Biologicals SA funded this study (GSK Study  identifier: HO-15-16768) | Critically low |
| (Eaton et al., 2019)  [66] | 3  (RCTs and cRCT)  /6 | Healthy children between 5 and 50 months (at enrollment) in Chinese urban pre-schools ; in rural and urban sites in  DRC, Guatemala, Pakistan, and Zambia (multi-sites, multi-country); and in Ecuador | Children receiving no intervention (2 studies) or an alternative intervention - fortified or unfortified cereals (1 study) | Provision of animal‐source foods or foods containing an animal‐source food: yogurt, egg, lyophilized (freeze-dried) beef product (1 study each).  Frequency was study-specific.  Interventions lasted between 6 months and 12 months | The results of the three trials were inconsistent on infectious disease morbidity outcomes (diarrhea, upper respiratory infections, pneumonia, severe pneumonia, and malaria)  There is insufficient evidence to assess the effect of this intervention on morbidity.  Because of the risk of biases in the studies, the evidence was graded as very low-quality.  No information on the health inequality effect of the intervention for the outcomes of interest for this umbrella review. | The Evidence and Programme Guidance Unit , World Health Organization (WHO), Switzerland  Bill & Melinda Gates Foundation, USA | High |
| (Esu et al., 2019)  [71] | 10  (RCTs)  /12 | Healthy infants living in malaria-endemic areas with moderate‐to‐high perennial transmission, regardless of the child's infection status. Infants were selected according to the patient list or the catchment area of individual clinics and health center in  Sub-Saharan Africa (Tanzania, Ghana, Gabon, Kenya, Mozambique, Uganda) | Infants receiving a placebo or no intervention | Intermittent preventive treatment in infant (IPTi) with 7 different combinations of antimalarial drugs delivered at home, or health centers or clinics; on their own or alongside vaccination or iron supplementation | IPTi reduces the incidence of clinical malaria (moderate to high certainty evidence), although its effects varied over time, from one drug combination to another and did not continue in the post-intervention follow-up.  Little to no effect on severe malaria incidence (low to moderate certainty evidence).  Sulfadoxine‐pyrimethamine-based therapies’ effects have reduced over time and so, artemisinin‐based combination therapies offer a good alternative for IPT to reduce clinical malaria.  No information on the health inequality effect of the intervention for the outcomes of interest for this umbrella review. | University Of Calabar, Nigeria; Liverpool School Of Tropical Medicine, UK; Department for International Development, UK. Project number 300342‐104 | High |
| (Flórez et al., 2015)  [78] | 3  (before/after studies, RCT)  /19 | Mothers and families with children under 5 living in India, Pakistan and Uganda | Target population before the interventions and/or population from neighboring communities receiving no intervention | Preventive health education (hygiene, immunization promotion, child health) intervention to households by community health workers (definitions and characteristics varied according to context) | Such interventions can reduce the risk of disease-specific morbidity in children (2 studies; low to moderate quality evidence)  They can improve immunization coverage (one study; high quality evidence).  The effectiveness of using these interventions to improve universal coverage of disadvantaged group has been poorly studied. | University of la Sabana | Critically low |
| (Freeman et al., 2017)  [66] | 33  (cross-sectional studies, non-randomized controlled trials, RCTs, case-control studies, controlled before-and-after studies)  /171 | Communities or households with children under 5 in 24 LMICs | Communities receiving no intervention, having no sanitation or lower levels of sanitation | Sanitation interventions which include provision or improvement of sanitation facilities or services (e.g., provision of household latrines or child potties), promotional activities (e.g., behavior change promotion to reduce open defecation) and community interventions, such as provision of sewage and wells | Sanitation has a protective effect against diarrhea (low quality evidence) and trachoma (moderate to high quality evidence) among children under-five (30 studies) but had no effect on STH and hookworm infections (5 studies, very low and low quality of evidence respectively).  High heterogeneity found in the meta-analyses. | World Health Organization, made possible through contributions from the UK Department for International Development and the Bill & Melinda Gates Foundation. | Low |
| (Gera et al., 2016)  [56] | 4  (cluster RCTs, controlled before and after studies)  /4 | Healthcare professionals, communities, infants and/or children under 5 years old in India, Bangladesh and Tanzania | Children receiving usual health services without the integrated health care package | Implementing the integrated management of childhood illnesses (IMCI) strategy, including improving health practices at health care facilities (e.g. training), at home (e.g. home visits), and in the community | IMCI had little or no effect on immunization coverage for measles or the third dose of diphtheria, pertussis, and tetanus (moderate certainty evidence) | Sitaram Bhartia Institute of Science and Research, India; Department for International Development, UK. | High |
| (Gulani & Sachdev, 2014)  [45] | 6  (RCTs)  /12 | Children under 5 in community or healthcare settings in Bangladesh, South Africa, Burkina Faso, India, Chile and Jamaica | Control placebo groups from the same population | Zinc supplements in different forms and dosage given at least once a week for at least four months | No evidence of a difference between zinc supplements and placebo on otitis media cases in children under 5 in community or healthcare settings (5 trials, good quality, with low risk of bias).  A potential or definite benefit among healthy or malnourished infants in community or healthcare settings, although the authors raised caution about some of the results due to sample size (3 trials, good quality, with low risk of bias).  The review could not assess whether that difference was due to age differences due to lack of comparable, age-stratified data across included studies.  The evidence on the effect of zinc supplemented on otitis media in children is mixed. | Sitaram Bhartia Institute of Science and Research, India | High |
| (Haider et al., 2017)  [57] | 4  (RCTs)  /12 | Term neonates up to 28 days after birth in healthcare or community settings, from both rural and urban areas in Guinea-Bissau, Indonesia, Zimbabwe and India. Pre-term and very low-birth weight babies were excluded but the studies were performed in areas with high health needs such as high prevalence of vitamin A deficiency and high risks of HIV. | Neonates receiving placebos | Synthetic vitamin A supplementation, with or without co-interventions, at the concentrations between 24,000 IU and 50,000 IU | The intervention has no significant effect on cause-specific infant mortality (4studies) and morbidity (3 studies)  (high quality evidence) | Centre for Global Child Health, The Hospital for Sick Children, Canada; Evidence and Programme Guidance, Department of Nutrition for Health and Development, World Health Organization, Switzerland; Eunice Kennedy Shriver National Institute of Child Health and Human Development, National Institutes of Health, Department of Health and Human Services, USA - Contract No. HHSN275201100016C | High |
| (A. Imdad et al., 2016)  [58] | 3  (RCTs)  /12 | Infants under 6 months in urban (2 studies) or undefined (1 study) settings in India, Ghana, Peru, Bangladesh and Indonesia | Infants from the same area or clinic receiving placebo | Synthetic vitamin A supplementation at the concentrations between 25,000 IU and 50,000 IU 3 times within 6 months during vaccination visits | The number of studies in cause-specific mortality and morbidity were small.  Vitamin A supplementation has no effect on disease-specific mortality (1 studies, moderate quality evidence) or morbidity (2 studies, low or high quality evidence) in this age group. | Sitaram Bhartia Institute of Science and Research, India; Max Hospital, India; Department of Nutrition for Health and Development, World Health Organization, Switzerland; Child and Adolescent Health Division, World Health Organization, Switzerland; Eunice Kennedy Shriver National Institute of Child Health and Human Development, National Institutes of Health, Department of Health and Human Services, USA - Contract No. HHSN275201100016C; National Institute for Health Research, UK - Cochrane Programme Grant (13/89/12). | High |
| (Aamer Imdad et al., 2020)  [70] | 21  (RCTs)  /49 | Preterm and/or low-birth-weight neonates in Asia (India, Turkey, Iran, China, Indonesia, Thailand) and Latin America (Mexico, Brazil).  All but two trials took place in hospital settings. | Preterm and/or low-birth-weight neonates receiving placebo or no intervention outside standard care | Oral probiotics/synbiotics supplementation in drops, oil solution or powder mixed with breast milk and/or formula at various dosage and frequency | Probiotics supplementation is effective in reducing the risk of sepsis in neonates (high certainty evidence; high quality trials). | Bill and Melinda Gates Foundation to the Center for Global Child Health at The Hospital for Sick Children, grant no. OPP1137750. | High |
| (Jarrett et al., 2015)  [63] | 7  (cluster RCTs, RCT, controlled trial, cohort studies)  /181 | Parents in low income settings or disadvantaged communities, including parents reluctant to immunization in Pakistan, Nigeria and India. Healthcare workers from primary healthcare centers involved in immunization programs in rural settings in Turkey. | Control groups received either no intervention or routine care. | Strategies addressing vaccine hesitancy (social mobilization; communication tool-based training/ information-based for healthcare workers; non-financial incentives) | All these interventions were found effective at population level, with dialogue-based interventions (e.g. social mobilization, training for health workers) considered most effective.  Two types interventions may be effective in addressing disadvantages when properly targeted: social mobilization (4 studies) and non-financial incentives (1 study). Communication tool-based training for health-care workers may not be as effective across all groups.  The evidence was of moderate to very low quality. | Bill & Melinda Gates Foundation, with additional support from the Center for Strategic and International Studies, EU Innovative Medicines Initiative (IMI), GSK, National Institute for Health Research (UK), Novartis, and WHO | Moderate |
| (Johri et al., 2015)  [59] | 11  (RCTs, cluster RCTs, before and after study)  /11 | Mothers, caregivers, households with children within the age group targeted by immunization; communities living in low- and middle-income settings and communities with lagging health and social indicators in Zimbabwe, Kenya, Pakistan, India, Bangladesh, Honduras and Ghana | Communities or parents from the same area or going to the same health center receiving no intervention, routine services and/or alternative interventions | Strategies to increase demand for vaccination (education or knowledge translation interventions and/or interventions using financial and non-financial incentives at the individual or community level) | Both educational approaches (7 studies) and use of incentives (4 studies) were effective strategies.  Demand-side interventions were associated with significantly higher receipt of vaccines.  Educational or knowledge translation interventions had a greater effect than incentives-based interventions.  There was considerable heterogeneity in the results and in the quality of the studies included (from low to high risks of biases). | Canadian Institutes for  Health Research (299960); Bill & Melinda Gates Foundation  (OPP1067851) | High |
| (Kosova et al., 2020)  [108] | 4  (RCTs)  /7 | Children under three years old in Africa (Ghana, Kenya, Mali) and Southeast Asia (Vietnam, Bangladesh) | Children under three years old receiving a placebo | Introduction of full course of vaccination a pentavalent live oral rotavirus vaccine into routine childhood immunization | Vaccine is effective in reducing incidence of severe rotavirus gastroenteritis.  Moderate risk of bias  The analysis covering countries with high mortality and low incomes showed a high level of homogeneity.  No information on the health inequality effect of the intervention. | Ural State Medical University  Ministry of Health of  the Russian Federation | Critically low |
| (Lamberti et al., 2016)  [79] | 25  (RCTs, observational studies)  /48 | Children under 5 years of age representative of the general population in four world regions | Healthy children from the same area, children with non-diarrheal illness, children with non-Rotavirus diarrhea or diarrhea-free controls | Introduction of rotavirus vaccines | The three studies assessing the effect of rotavirus vaccination programs on child diarrhea mortality found a reduction of diarrhea mortality in Latin American countries (moderate quality; consistent results across studies).  The 22 studies assessing the number of diarrhea episodes of any severity, any cause found a protective efficacy and effectiveness of rotavirus vaccination against rotavirus and all diarrheal outcomes (moderate or high quality).  Rotavirus vaccination was efficacious and effective in all MDG regions, but was highest in East/Southeastern Asia, followed by Latin America and the Caribbean, South Asia and sub-Saharan Africa. | Maternal Child Epidemiology Estimation  (MCEE) grant from the Bill & Melinda Gates Foundation | Critically low |
| (Lassi et al., 2016)  [60] | 6  (RCTs)  /6 | Children aged from two to 59 months in community or healthcare settings. Most studies occurred in disadvantaged urban areas in Bangladesh, India, Peru and South Africa, with two studies including HIV-affected children. | Children receiving a placebo | Daily oral zinc supplementation (with or without other supplements) | Zinc supplementation was significantly associated with reducing the incidence (6 studies) and prevalence (1 study) of pneumonia in children under 5 (low quality evidence).  A specific sub-group (HIV positive children, 1 study) showed similar results (low quality evidence) – suggesting a neutral effect on health inequalities based on these characteristics. | Aga Khan University, Pakistan. | High |
| (Lukusa et al., 2018)  [64] | 6  (RCT, cluster RCT)  /6 | Parents and guardians in both rural and urban areas in India, Pakistan and Nepal | Caregivers receiving no intervention | Educating caregivers in communities or in health facilities about the importance of childhood vaccination | Educating parents substantially improve in childhood vaccination coverage (moderate certainty evidence).  No difference between the effects of community-based (3 studies) and health facility-based (3 studies) education.  Quality of the studies was considered high (3 studies) or moderate (3 studies with high risks of bias). | South African Medical Research Council and the National Research Foundation of South Africa (Grant Numbers: 108571 and 106035). | Moderate |
| (Majorin et al., 2019)  [69] | 16  (RCTs; controlled before after studies; cohorts and cross sectional studies)  /63 | Households with children under 5 years old in rural and urban communities living in  Sub-Saharan Africa (Chad, DRC, Rwanda, Ethiopia, Tanzania, Mali) and Asia (India, Indonesia, Bangladesh) | Communities receiving either no intervention, standard services or an alternative intervention | Intervention to improve the disposal of child feces: education and hygiene promotion interventions, community-led sanitation interventions (with or without adaptations) and WASH infrastructure provision | Apart from WASH infrastructure provision and one type education and hygiene promotion, most of the interventions showed no impact on diarrhea or STH infection.  One study found opposite effects of its education and hygiene promotion intervention between its rural and urban slum sites.  As the child feces disposal component of the intervention was combined with other elements, the evidence on the effectiveness of safe disposal of child feces on diarrhea or STH infection is limited. The quality of the studies was moderate to very low. | Liverpool School of Tropical Medicine, UK  Bill & Melinda Gates Foundation, USA.  Department for International Development, UK. Project number 300342‐104 | High |
| (McGuinness et al., 2018)  [67] | 6  (cluster RCTs)  /14 | Caregivers, parents and households with children under 5 in urban childcare settings or urban or rural domestic settings in China, Colombia, Bangladesh, Kenya, Peru and Pakistan | Controls with similar sociodemographic characteristics as the intervention groups, from another childcare center or geographic area, receiving standard care practice or alternative interventions | Hygiene education interventions alone or with infrastructure interventions including the provision of hygiene products delivered in childcare, school or domestic settings. | Hygiene intervention can lead to a reduction in ARI illness (5 studies, low quality evidence) and pneumonia incidence (1 study, high quality evidence).  The interventions’ impact varied according to setting, intervention target and compliance.  Hygiene interventions reduced child ARI illness in urban childcare settings (2 studies, low-quality evidence) but not in rural domestic settings (3 studies, low-quality evidence). | National Health and Medical Research Council of Australia (Career Development Fellowship APP1068732 to A.C.C and APP1084351 to K.L., Postgraduate Scholarship APP1115196 to S.L.M.). | Low |
| (Mekonnen et al., 2019)  [74] | 5  (RCT)  /10 | Infants and their caregivers recruited at the health facility or in the village in four countries of Central America (Guatemala) and Sub-Saharan Africa (Kenya, Nigeria and Zimbabwe) | Caregivers or children receiving routine/standard care | Mobile phone text messages providing reminders related to vaccinations. The frequency of messages and timing of the text message reminders was different in the included studies. | Statistically significant positive effect of text message reminders on vaccination uptake (definitions varied across studies), with moderate heterogeneity.  Due to the risk of bias and heterogeneity the quality of the body of evidence was low.  While current research showed promising results, more research is needed.  No information on the health inequality effect of the intervention for the outcomes of interest for this umbrella review. | None | Moderate |
| (Morita et al., 2016)  [80] | 4  (pre-/post-evaluation studies)  /8 | Household with children under 5 years old in rural or urban settings in Bangladesh | Target households before the intervention and/or households from the same area receiving no interventions | Hygiene education with the installation of water and sanitation infrastructure or hygiene education alone. | These interventions are effective in reducing diarrhea incidence (2 studies) and ascariasis prevalence (1 study) but have no effect on morbidity cause by respiratory diseases (1 study).  The positive effects found for two of the health outcomes were greater for ambulatory children than for younger ones but the limitations of included studies made it difficult to attribute these effects to the interventions to reduce unsafe disposal of child feces alone (vs. the full WASH intervention).  There is a lack of evidence on the effectiveness of interventions targeting unsafe child feces disposal. | Johns Hopkins University | Critically low |
| (Munk et al., 2019)  [85] | 14  (RCTs; observational studies with or without controls)  /14 | Infants and their caregivers living in community of intervention. Observations were nearly evenly split between rural and urban setting. Two studies specifically targeted unvaccinated children and 1 study targeted urban population at risk of cholera. | Unclear | Interventions aiming to increase infant vaccination coverage: vaccine delivery (mobile teams, outreach, campaigns), immunization demand generation (cash transfers, community discussions, community health workers), health system strengthening (multilevel/multisector strengthening, immunization planning and financing) and new technology introduction (new syringes) | The majority of interventions led to an increase in vaccination coverage.  Risk of biases in several studies and the high heterogeneity in the methodology and interventions covered. | Bill & Melinda Gates Foundation | Low |
| (Mureed et al., 2015)  [81] | 10  (RCTs; Quasi-experimental studies with control groups)  /10 | Mothers and communities with children of vaccination age in community settings in Pakistan, Nepal, Kenya, Ghana, Malawi, Iraq and India | Details not reported | Professional interventions (e.g. health education and community outreach with or without immunization services) to educate about and improve immunization coverage for 7 childhood cluster diseases. | This type of interventions is effective in increasing vaccination coverage for these diseases, especially for DPT and measle vaccines.  It has limited effect for polio or the overall vaccination schedule coverage.  Among the 10 studies, 3 were assessed to be high quality studies, 6 as medium quality and 1 low quality | Rachadapisek Sompote  Fund for Postdoctoral Fellowship, Chulalongkorn  University | Critically low |
| (Naugle & Hornik, 2014)  [82] | 7  (Non-randomized controlled trials; Before and after studies)  /106 | Caregivers in rural or urban communities (studies on immunization) in Bangladesh, Democratic Republic of the Congo, Ecuador, Lesotho, Mexico, Peru and Philippines.  The general public in Cameroun (the evaluation was amongst adult over age 15) | Caregivers in neighboring communities receiving no intervention or target caregivers /public before the intervention | Media campaigns at community level, with or without service outreach, to improve immunization coverage.  Mass media campaign to improve last night bed net use in children under 5 | Mass media campaigns can positively impact selected health behaviors such as bed net use (1 campaign assessed as strong) and immunization (of the campaigns covered, 4 were assessed as strong, 3 moderate, 1 weak) in children under 5 in low- and middle-income countries.  Caution about the sample, evaluation design and publication biases affecting the quality and generalizability of the evidence currently available. | USAID and the Annenberg School for Communication | Critically low |
| (Nelson et al., 2016)  [83] | 14  (RCTs, before and after studies, cross-sectional studies)  /14 | Healthcare professionals providing immunization services and/or caregivers of children under 5 in African, Eastern Mediterranean or South-east Asian countries (Pakistan, Ghana, Mozambique, Bangladesh, Philippines, Zambia, Sudan, Kenya, Brazil and India) | Children before the intervention or children receiving either no intervention or an alternative intervention. One study didn’t have a clear comparison group. | Intervention aiming at improving the availability of routine immunization services by healthcare providers, Increasing their utilization by caregivers, or a combination of both types of interventions | All the interventions included are effective in increasing the uptake and/or coverage of childhood immunization in urban settings, with home visits and parental education having the potential to address the disadvantage of high risk or minority groups.  Health facility-based interventions are more likely to be effective in urban than rural areas in improving immunization update due to distance, thus creating a risk to increase disparities between these areas (one study).  Only five of the studies covered were at low risk of bias. | None | Critically low |
| (Ngocho et al., 2019)  [68] | 8  (before/after studies)  /8 | Children under 5 in African countries (South Africa, Morocco, Gambia, Mozambique, Kenya, and Burkina Faso) | Children before the introduction of the vaccine | Introduction of PCV vaccines in national childhood immunization programs, with or without catch-up doses | Invasive pneumococcal disease declined after the introduction of the vaccine, with the highest decline found in younger children (under 2 years old), the group carrying a higher proportion of pneumococcal infection (studies’ quality good to fair).  Due to the heterogeneity of the studies, a meta-analysis was not possible. | Fogarty International Center of the National Institutes of Health (Award Number D43TW010138); German Academic Exchange Service (Deutscher Akademischer Austauschdienst-DAAD) | Low |
| (Odendaal et al., 2018)  [46] | 2  (cluster RCT; controlled before-after study)  /2 | Rural, under‐resourced communities in Cambodia and Guatemala | Communities receiving no intervention | Contracting out governmental preventive, promotional and/or curative health services to non‐governmental service providers | Contracting out to non-governmental provider has little or no effect in immunization uptake of children under 2 y.o. (2 studies, moderate‐certainty evidence).  Contracting out has little or no effect on the incidence of childhood diarrhea (1 study, low‐certainty evidence). | Alliance for Health Policy and Systems Research | High |
| (Oliver-Williams et al., 2017)  [69] | 21  (RCTs; observational studies with or without control)  /21 | Families or caregivers with children within the vaccination age; healthcare professionals involved in vaccination, in LMICs with large numbers of unvaccinated children (Bangladesh, India, Kenya, Malawi, Nigeria, Pakistan, The Philippines, Zambia, Zimbabwe) | Families receiving routine care or alternative intervention. Households or health facilities receiving no intervention; target households before the implementation of the interventions | mHealth interventions such as reminders and information sent to families or caregivers; apps and mobile technologies for healthcare professionals to keep records or facilitate immunization campaigns. | These interventions may be effective in improving vaccine coverage in these countries.  Research and evidence quality are still limited and several limitations were identified in the studies covered. | Medical Research Council, British Heart Foundation, and Homerton College, Medic Mobile, Polygeia, UK. | Low |
| (Onwuchekwa et al., 2020)  [104] | 6  (case-control studies; interrupted time-series; pre-post studies)  /8 | Infant between 6-week and 9-month old  Sub-Saharan Africa (Gambia, South Africa, Kenya) | Populations before and after the introduction of the vaccine into routine vaccination schedule  Case-control studies included controls in the community and/or in hospitals | Routine administration of 7-valent, 10-valent and 13-valent pneumococcal conjugate vaccines (PCV) after its introduction into routine vaccination schedule. Frequency and schedule are country-specific | Routine administration of the PCV reduced the risk of radiological and pneumococcal pneumonia in children under 5 years old, particularly in the age group between 12 to 23-month.  The population impact of PCV vaccination depends on the definition of pneumonia used.  The quality and risk of biases in the included study varied. | None | Critically low |
| (Owusu-Addo & Cross, 2014)  [70] | 8  (controlled before and after study; cluster RCTs)  /16 | Households from poor and/or disadvantaged groups (e.g. rural population; suffering from malnutrition) from Mexico, Columbia, Honduras, Nicaragua and Zimbabwe | Communities from the same area or neighboring districts receiving no intervention | Conditional cash transfers (CCTs) provided at the community level | CCTs are associated with reduction in morbidities of diarrhea and ARI among under 5-year-old children in Mexico and Colombia (high to moderate quality level evidence, 3 studies).  CCTs are effective in improving vaccination coverage (5 out of 6 studies, moderate to high quality) among under 5-year-old children in Mexico, Colombia, Honduras, and Nicaragua, although three studies noted that no effect was found in children over 2 years old. | Not reported | Low |
| (Oyo-Ita et al., 2016)  [61] | 14  (RCTs; cluster RCTs)  /14 | Healthcare professionals involved in immunization, and caregivers of children under 5 in mainly vulnerable groups or groups and communities with higher health needs in Georgia, Ghana, Honduras, India, Mali, Mexico, Nicaragua, Nepal, Pakistan, Zimbabwe | Controls received routine care or no interventions. One study offered  an alternative intervention. | Recipient-oriented interventions (community-based health education, facility-based health education, household monetary incentives), provider-oriented intervention (health professionals training) and health system-oriented interventions (home visits, outreach activities; integration of services; quality improvement) either as single interventions | Community-based or facility-based health education probably improve immunization coverage (6 studies, moderate- to low-certainty evidence).  Regular immunization outreach, integration of services and home visit to identify and refer unvaccinated children may improve immunization (4 studies, low-certainty evidence).  Household monetary incentives (4 studies, low-certainty evidence) and health professionals training (1 study, low-certainty evidence) may have little or no effect on immunization coverage.  The authors note that due to the low quality of the evidence, the actual effect of the interventions may vary substantially. | Vaccines for Africa Initiative, University of Cape Town (CW), South Africa; Norwegian Agency for Development Cooperation (Norad), Norway; Research Council of Norway, Norway. | High |
| (Puchalski Ritchie et al., 2019)  [71] | 6  (cluster RCTs)  /18 | HIV-positive mothers and their infants in South Africa, Kenya, Mozambique and Nigeria | HIV-positive mothers and their infants receiving with no intervention or usual care | Integration of PMTCT with other healthcare services, service quality improvement and outreach to in improve uptake and retention of HIV-positive mothers and their infants in these services | One outreach intervention increased service HIV prophylaxis uptake in infants (low quality evidence).  The remainder of interventions had no significant effect or decreased infant prophylaxis uptake.  The majority of studies were of moderate to high risk of bias.  Heterogeneity of interventions and outcome reported limited both comparison across studies and intervention categories, as well as opportunities for meta-analysis. | KT Canada Strategic Training Initiative in Health Research Fellowship award; Canada Research Chair in Knowledge Translation and Quality of Care; National Institute of Mental Health (Grant K99 MH104154-01A1); National Institute of Allergy and Infectious Diseases (P30 AI50410 and R01 AI131060-01). | Low |
| (Saeterdal et al., 2014)  [62] | 2  (cluster RCTs)  /2 | Communities in poor resource settings with low immunization rates in India and Pakistan | Communities receiving no interventions | Interventions by trained community members aimed at disseminating vaccination information and build awareness about childhood vaccination such as community meetings; information campaign using printed materials (brochures and pamphlets), electronic media (audio recordings) | Interventions aimed at communities to inform and educate about childhood vaccination may improve the immunization status of children (low certainty evidence).  The interventions were too heterogenous to be combined in a meta-analysis. | Norwegian Research Council for the 'Communicate to vaccinate' (COMMVAC) project | High |
| (Saleh et al., 2020)  [105] | 6  (RCTs and cRCTs)  /14 | Households with children under 5 years old in Latin America (Peru, Guatemala, Mexico), Africa (Malawi, Rwanda) and Asia (Nepal). Three trials taking place in rural communities. | Households with children using their traditional cooking methods | Improved cookstoves (e.g., with more efficient combustion, or chimneys for ventilation, etc.) at household level to reduce exposure to air pollution. | Improved cookstoves have no statistically significant effect on child pneumonia incidence.  The high methodological heterogeneity across studies prevented from providing pooled estimates of the intervention’s impact.  Two trials were at low risks of bias, three presented some risks and one was high risk.  No information on the health inequalities effect of the intervention. | National Institute for Health Research(NIHR; London, UK) Global Health Research Unit on Lung Healthand TB in Africa at the Liverpool School of Tropical Medicine, ‘‘IMPALA’’ (grant number 16/136/35), UK aid from the UK Government.  Wellcome Trust Clinical PhD Fellowship (University of Liverpool, Liverpool, UK, block award [203919/Z/16/Z]). | Critically low |
| (Santos et al., 2016)  [84] | 31  (cross-sectional, case-control study, cohort and ecological studies)  /215 | Children under 5 years old in hospital and/or community settings in Latin American countries (Argentina, Brazil, Mexico, Venezuela, Bolivia, El Salvador, Nicaragua and Panama) | Pre-introduction period | Introduction of rotavirus vaccines in national childhood immunization programs, including universal rotavirus immunization | Decrease in the proportion of rotavirus infections and morbidity of any severity in the postvaccine era compared to the pre-vaccine period.  Universal immunization led to a reduction of rotavirus mortality in infants (12 studies).  The studies’ quality was not reported. | Edital MCTI/CNPq N° 14/2013 (#471747/2013-0) and Edital MEC/MCTI/CAPES/CNPQ/FAPS - PVE 2014 (#400723/2014-0). | Critically low |
| (Smith et al., 2018)  [85] | 6  (cohort studies, before and after studies without a control, controlled observational studies)  /28 | Children under 5 and their caregiver or families in healthcare or community settings in rural and urban areas in Sub-Saharan African countries (Tanzania, Kenya, Zambia, Zimbabwe, South Africa and Swaziland). | Children and families receiving no intervention or routine care | Integrating HIV services with immunization services or post-partum care | Such integration of HIV services has either a positive or a neutral effect on immunization uptake and increased the uptake of HIV treatment prophylaxis (fair to good quality of evidence).  However, in one study immunization uptake increased in urban sites but decreased in rural sites, suggesting a detrimental effect of the intervention on health inequalities (poor quality of evidence). These differences were attributed to clients’ stigma and discrimination concern, amplified in the rural sites. | Global Health Fellows Program-II, USA | Critically low |
| (Soares‐Weiser et al., 2019)  [68] | 12  (RCTs)  /55 | Healthy infants and/or infants with health conditions (e.g. malnutrition or HIV) living in high child mortality countries (Bangladesh, India, South Africa, Malawi, Brazil, Mexico, Venezuela, Ghana, Kenya, Mali, Botswana, Tanzania, Zambia, Zimbabwe, Vietnam | Infants receiving placebo or no intervention | Three rotavirus vaccines prequalified by the WHO (RV1, RV5, and Rotavac) introduced into routine childhood vaccination, given alongside other routine vaccinations. Doses and frequency varied from trial to trial. | Vaccines are efficacious in preventing rotavirus diarrhea in children under 5 years old in high mortality countries (moderate‐ to high‐certainty evidence).  Vaccine efficacy against all‐cause diarrhea was lower (moderate‐ to high‐certainty evidence).  Vaccines’ efficacy was lower in children 12 to 24 months old than in infant.  RV1 is efficacious in preventing rotavirus diarrhea in malnourished children but neither RV1 or RV5 have a statistically significant effect amongst HIV-exposed or infected children. | Liverpool School of Tropical Medicine, UK.  Department for International Development (DFID), UK. Project number 300342‐104  Initiative for Vaccine Research (IVR), World Health Organization (WHO), Switzerland. | High |
| (Soboksa et al., 2020)  [77] | 7  (RCT and cRCT)  /10 | Households with children under 3 or 5 years old in both rural and urban settings in Ethiopia; Kenya; South Africa; Bolivia; Cambodia; India and Peru | Households with children receiving no intervention | Water quality improvement: Solar disinfection water treatment (SODIS) | Using SODIS water treatment reduced the risk of diarrhea by 41% in children under 5 years old, although there are heterogeneity in the analysis and variations based on the length of follow-up.  Some studies presented some risks of bias but the sensitivity analysis suggests these studies do not affect the results significantly. The risk reduction in children under 5 varied between world regions, although they all showed a reduction.  Although the meta-analysis showed a positive results of SODIS water treatment on diarrhea incidence, further high-quality studies were needed to confirm these findings. | None | Moderate |
| (Suchdev et al., 2020)  [67] | 9  (RCTs and cRCTs)  /29 | Infants and children under two years old living in rural, slum and urban settings, including areas with low socio-economic indicators in Asia (Laos, India, Bangladesh, Pakistan, Cambodia) and Sub-Saharan Africa (Ghana, Mali, Burkina Faso)  Three studies in malaria-endemic areas. | Infant and children from the same or neighboring communities receiving placebo, no intervention or an alternative intervention | Point‐of‐use food fortification with multiple micronutrient powders (MNP) sachets, mixed with food before consumption daily or every two days at home. | No effect of MNP food fortification on diarrhea, upper respiratory infections, malarial infection.  Four trials assessed as high quality and two as low quality.  Two of the analyses showed moderate to high heterogeneity. Information is insufficient to assess effects of MNP on these outcomes. No information on the health inequality effect of the intervention for the outcomes of interest for this umbrella review. | Emory University, USA  Nutrition International, Canada  Evidence and Programme Guidance Unit, Department of Nutrition for Health and Development, World Health Organization (WHO), Switzerland | High |
| (Tam et al., 2020)  [107] | 25  (RCTs)  /197 | Healthy infants and children under five years old living  in Latin America (Mexico, Ecuador, Honduras, Guatemala), Sub-Saharan Africa (Guinea Bissau, Tanzania, South Africa, Mali) and South Asia (Indonesia, Bangladesh, India, Nepal, Vietnam, Pakistan) | Infants and children receiving placebos, no interventions or an alternative nutrition intervention | Micronutrient supplementation interventions: Vitamin A, zinc, iron (with or without folic acid) or multi-nutrients supplementation in the form of capsules, tablets, drops, syrup, fortified flour or sachets. Dosage, form and frequency varied between studies. | Zinc supplementation is effective in reducing the risk of diarrhea (11 studies) but not the risk of lower respiratory tract infection (LRTI).  Vitamin A, iron and/or folic acid and multi-nutrient supplement in tablets/capsules have no effect on either diarrhea or LRTI incidence. Fortified food or multi-nutrient powder sachets at point-of-use increase the risk of diarrhea (4 studies - more research needed on this finding).  Moderate to high heterogeneity in three of the meta-analyses.  Results of the risk of bias assessment not provided.  No information on the health inequality effect of the intervention for the outcomes of interest for this umbrella review | Bill and Melinda Gates Foundation (grant number OPP1137750) | Critically low |
| (Thakur et al., 2018)  [86] | 4  (RCTs, cohort study)  /53 | Households with children under 5 in rural settings in Kenya, Peru, Malawi and Guatemala | Households from the same or neighboring community using traditional stoves | Improved cookstoves (biomass fuel cookstove improvements) to reduce morbidity due to household air pollution | There was no significant change in pediatric acute respiratory infections and severe pneumonia (strong evidence). | Intermediate Fellowship by the Wellcome Trust DBT India Alliance (Clinical and Public Health Research Fellowships); Netherlands Lung Foundation (4.2.14.063JO) and the Erasmus MC. | Low |
| (Vardanjani et al., 2019)  [110] | 3  (matched case-control studies)  /10 | HIV infected and uninfected infants in South Africa | Unvaccinated children, including HIV uninfected children | Introduction into routine immunization of the pneumococcal conjugate vaccines PCV7 or PCV13 injected at 6 weeks, 14 weeks and 9 months | PCV were effective at population level in reducing invasive pneumococcal disease morbidity in children under 5 years old and 2.5 years old  PCV effectiveness was significantly lower amongst HIV-infected children.  PCV effectiveness was lower amongst younger children (under 2.5 years old) than in older children (under 5 years old).  PCV 7 effectiveness was higher than that of PCV13, especially amongst HIV infected children.  The included studies were assessed as low risk of bias and the meta-analyses had moderate to low heterogeneity | None | Critically low |
| (Velazquez et al., 2017)  [87] | 4  (case-control studies)  /13 | Infants in Latin American countries (Brazil, El Salvador and Nicaragua) | Pre-introduction period | Introduction of rotavirus vaccines (RV5 and RV1) in national childhood immunization programs | Both vaccines were found effective against more severe rotavirus gastroenteritis in children under 5 presenting at a health facility.  The author did not provide information on the quality of the case-control studies. | GlaxoSmithKline Biologicals SA | Critically low |
| (Wang et al., 2016)  [109] | 4  (cluster RCTs)  /19 | Infants living in remote and/or rural townships in China | Infants receiving alternative intervention (standard or alternative Hepatitis B immunization strategies) | Out-of-cold chain strategies for the Hepatitis B vaccine delivered in the community or at health facilities | Out-of-cold chain strategies increase in Hepatitis B vaccine coverage amongst children, especially of the first dose given to newborns.  Coverage increases are higher amongst disadvantaged groups such as babies born at home.  Quality of the studies and evidence is moderate. | China-UK Global Health Support Pro-gramme (Grant No. GHSP-CS-OP1-02) | Critically low |
| (Willame et al., 2018)  [88] | 17  (case-control studies)  /29 | Infants in Ghana, Malawi, Zambia, Brazil, Columbia, El Salvador, Guatemala, Botswana, Moldova, South Africa, Bolivia, Armenia, Mexico | Pre-introduction period | Introduction of a specific brand of rotavirus vaccine (Rotarix©) in immunization programs | The vaccine was effective in reducing gastroenteritis morbidity in children younger than 5 across various geographic and economic settings (strong evidence)  There are differences between country income groups (4 studies) and a higher vaccine effectiveness in children under 1 year old compared to children between 1 and 5 (5 studies).  The review excluded low quality studies. | GlaxoSmithKline Biologicals S.A. | Critically low |
| (Win Han et al., 2019)  [76] | 3  (cluster RCT and controlled quasi-experimental study)  /28 | Households with children under 5 years old living in malaria endemic areas in Cameroon, Nigeria, Uganda and India | Communities not receiving community health services | Two different community-delivered models: the Home Management of Malaria model (a household member provides malaria services to the household and their neighbors) and the traditional community health worker model (the health authorities train and equip a member of the community to provide malaria services) | Two out of three studies found that community-delivered models increased the use of bednets in children under 5 years old.  Overall, community-delivered models are effective in increasing coverage of malaria interventions, such as bed nets, in population living in malaria-endemic areas. Heterogeneity and moderate to high risks of bias affecting these results. | Deakin-Burnet PhD scholarship (to WHO), Australian Research Council (Future Fellowship to FJIF)  Australian National Health and Medical Research Council (Career Development Fellowship and Australian Centre for Research Excellence in Malaria Elimination to FJIF).  Burnet Institute is funded (Victorian State Government Operational Infrastructure Support grant) | Moderate |
| (White et al., 2014)  [75] | 4  (RCTs)  /7 | Babies of HIV-infected, breastfeeding mothers recruited in health facilities, primarily in urban settings in Sub-Saharan African countries (Malawi, Ethiopia, Uganda, South Africa, Tanzania, Uganda, Zimbabwe) and India | Infants receiving ARV at birth and for up to one week | Extended ARVs prophylactic treatment to reduce mother‐to‐child HIV transmission through breastfeeding: extended regimen of one or two ARVs for up to 6 months, with or without ARVs being provided to the mother | Moderate- to high-quality evidence that extended ARV infant prophylaxis during breastfeeding is associated with lower risks of HIV infection.  Heterogeneity in the dosage, regimen and duration prevented having summary effect size on the risk of infant HIV infection. | Global Health Sciences, University of California, San Francisco, USA.  World Health Organization, Switzerland. The World Health Organization (WHO) commissioned the first iteration of this review in 2009 to inform WHO’s 2010 PMTCT guidelines | Moderate |
| (Yuan et al., 2014)  [47] | 3  (cohort studies and repeated cross-sectional study)  /22 | among infants living in households with different socio-economic or demographic characteristics in Bangladesh, South Africa, Zambia | Target population before the intervention, infants from the same cohort with different characteristics or non-vaccinated controls. | Outreach immunization services to increase measles immunization rates | Immunization outreach campaigns can reduce inequalities in immunization rates across different levels of education, living area and distance to health facilities - suggesting a positive effect on health inequalities. | e Swedish International  Development Cooperation Agency (Sida) | Low |
| (Yunusa et al., 2021)  [106] | 9  (RCTs)  /10 | Caregivers of children under 2 years old (recruited when the child was a newborn or an infant) in Pakistan, Guatemala, Ethiopia, Nigeria, Zimbabwe, Kenya, Ivory Coast | Caregivers of children under 2 years old receiving to intervention | Use of mobile phone reminders to enhance routine childhood immunization: SMS, phone calls or both to reminding of upcoming immunization (BCG, OPV, DPT3, penta 1, penta 2, penta 3, measles and yellow fever vaccines) | Mobile phone reminders improved immunization coverage (moderate quality evidence)  The use of phone calls or calls combined with SMS has a higher effect on immunization coverage. Effect on immunization uptake is unclear (low certainty evidence). High heterogeneity of the results and risks of bias in the included studies.  No information on the health inequalities effect of the intervention. | Tertiary Education Trust Fund (TETFund), Nigeria (BUK/R/T-EPIC/SB5/N16). European Respiratory Society Fellowship in Guidelines Methodology (MTF-2019-01). | Critically low |
