# Supplementary material for: Which public health interventions are effective in reducing morbidity, mortality and health inequalities from infectious diseases amongst children in low- and middle-income countries (LMICs): An umbrella review
Source: PLoS One. 2021 Jun 10;16(6):e0251905. doi: 10.1371/journal.pone.0251905 (PMC8191901; doi:10.1371/journal.pone.0251905)
Supplement: Attachment — Submitted filename: PLOS-Review .docx [file pone.0251905.s010.docx]

Which public health interventions are effective in reducing morbidity, mortality and health inequalities from infectious diseases amongst children in low- and middle- income countries (LMICs): an umbrella review.

PONE-D-20-17157

The authors sought to investigate the effectiveness of public health interventions in reducing morbidity, mortality and health inequalities in children younger than 5 years with infectious diseases by using an umbrella review.

This is a well written manuscript. The narrative of the methods flows smooth and the reader is confident of the results because of the detailed description of the methods.

Page 21 line 258 Quality of the evidence: because low and critically low quality studies were included in several analyses, the manuscript will be enhanced if at the end of each section the authors will include only high and moderate quality studies (resembling a sensitivity analysis). I see a summary in some of the sessions at the end of the manuscript but not at the beginning.

Page 33 line 420 Protecting children from infectious diseases. The authors did not find any review that protected specifically children from infectious diseases and approached population and health inequalities. I am wondering if the reviewers included all interventions guided to promote vaccination. Clarifying this point will be helpful.
